# Deep Proteome Analysis Identifies Age-Related Processes in *C. elegans*

**DOI:** 10.1016/j.cels.2016.06.011

**Published:** 2016-08-24

**Authors:** Vikram Narayan, Tony Ly, Ehsan Pourkarimi, Alejandro Brenes Murillo, Anton Gartner, Angus I. Lamond, Cynthia Kenyon

**Affiliations:** 1 Centre for Gene Regulation and Expression, School of Life Sciences, University of Dundee, Dundee DD1 5EH, UK; 2 Department of Biochemistry and Biophysics, University of California, San Francisco, San Francisco, CA 94158-2517, USA; 3 Calico Life Sciences, 1170 Veterans Boulevard, South San Francisco, CA 94080, USA

## Abstract

Effective network analysis of protein data requires high-quality proteomic datasets. Here, we report a near doubling in coverage of the *C. elegans* adult proteome, identifying >11,000 proteins in total with ∼9,400 proteins reproducibly detected in three biological replicates. Using quantitative mass spectrometry, we identify proteins whose abundances vary with age, revealing a concerted downregulation of proteins involved in specific metabolic pathways and upregulation of cellular stress responses with advancing age. Among these are ∼30 peroxisomal proteins, including the PRX-5/PEX5 import protein. Functional experiments confirm that protein import into the peroxisome is compromised in vivo in old animals. We also studied the behavior of the set of age-variant proteins in chronologically age-matched, long-lived *daf*-*2* insulin/IGF-1-pathway mutants. Unexpectedly, the levels of many of these age-variant proteins did not scale with extended lifespan. This indicates that, despite their youthful appearance and extended lifespans, not all aspects of aging are reset in these long-lived mutants.

## Highlights

• Near doubling in *C. elegans* proteome coverage with age-dependent protein measurements
• Putative sub-cellular localization assignments for >6,000 nematode proteins
• Peroxisome protein import impaired during aging
• Most age-variant proteins do not scale with biological age in insulin/IGF-1 mutants

## Graphical Abstract

**Figure:**
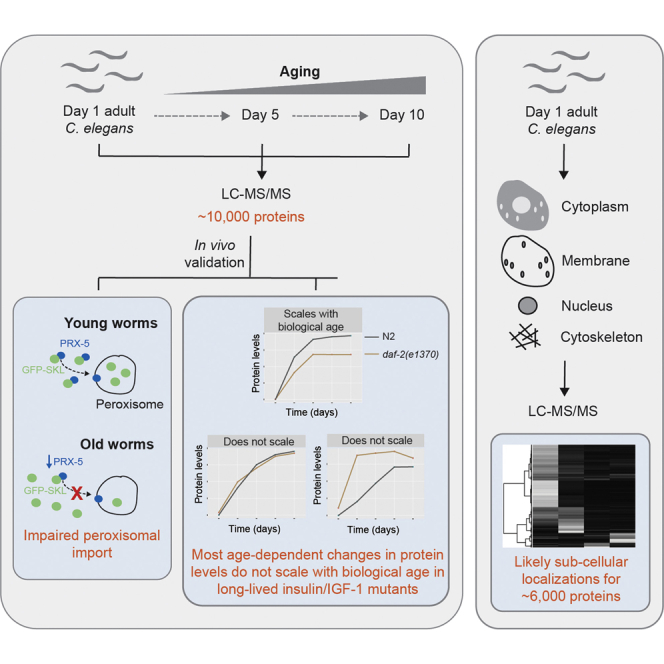

## Teaser

Deep proteome analysis of adult *C. elegans* provides a valuable resource, reveals a failure in peroxisome protein import during aging, and, surprisingly, shows that the rates of change in the levels of the majority of age-variant proteins do not scale with the reduced rate of biological aging in long-lived insulin/IGF-1 mutants.

## Introduction

The nematode *C. elegans* has been used widely to study aging due to its short lifespan and hermaphroditic reproductive cycle, as a result of which large numbers of isogenic animals can be cultured easily. Much of what we know about how aging is regulated was discovered through genetic perturbations in *C. elegans*. For example, lifespan extension through downregulation of insulin/IGF-1 signaling, and later TOR, was first described in these animals (Kenyon et al., 1993, Vellai et al., 2003, Kimura et al., 1997). Since then, dozens of genes, primarily affecting the sensation of, or response to, stress and nutrient sensing, have been found to affect lifespan. Many of these genes also are associated with aging in other organisms, including mammals (Kapahi et al., 2010, Kenyon, 2010).

In comparison with detailed studies on the genetic control of aging, relatively less is known about how the proteome changes with age. There is evidence for increased protein aggregation and a gradual failure of the ubiquitin-proteasome machinery with advancing age, leading to a disruption of proteostasis (David et al., 2010, Taylor and Dillin, 2011, Morley and Morimoto, 2004). Only a handful of proteomic-based aging studies have been reported to date, so far with limited global protein coverage and resolution. Recently, the opportunity to apply quantitative proteomic approaches in *C. elegans* has been aided by the development of a stable-isotope labeling with amino acids in cell culture (SILAC)-based methodology for nematodes (Larance et al., 2011, Fredens et al., 2011). A database of mRNA and protein-abundance changes during nematode development also has been compiled (Grün et al., 2014) and age-dependent changes measured (Walther et al., 2015, Liang et al., 2014, Dong et al., 2007, Zimmerman et al., 2015, Copes et al., 2015).

Consistent with the measurement of transcript and protein levels during development (Grün et al., 2014), recent findings in worms have reported an unexpectedly weak correlation between mRNA-level changes and protein-level changes during aging, suggesting that a significant proportion of age-related changes in protein levels are regulated at the post-translational level (Walther et al., 2015). However, current reports quantify only about 25% of the theoretical proteome, i.e., 5,000 proteins of ∼20,000 protein-coding genes, although not every protein is likely to be expressed at all times. Together, these findings highlight the need to measure protein-level changes, and they underscore the importance of expanding the depth of protein coverage to provide a better understanding of aging. Having in-depth, high-quality proteomic datasets also will expand the number of validated protein-coding genes, and it will aid in the development of novel network analysis tools based on proteins, rather than mRNAs.

In this study, we expand the coverage and quantification of the *C. elegans* proteome, identifying >9,300 protein groups in each of three biological replicates. We characterize how organismal aging affects the levels of a large subset of these proteins (7,380), providing a proteomic fingerprint of the aging process. This expanded analysis of the proteome identifies a large set of proteins whose abundances change with age. These data, coupled with in vivo biological experiments, also identify the concerted downregulation of specific cellular metabolic networks during aging and uncover an age-dependent dysregulation of the peroxisome. In addition, combining our new findings with a detailed analysis of previously published datasets shows that only a subset of proteins whose abundances change with age in wild-type animals scale with the rate of biological aging in long- and short-lived insulin/IGF-1 pathway mutants.

## Results

### Deep Proteome Analysis in *C. elegans* Reproducibly Identifies 9,398 Proteins

**Figure 1:**
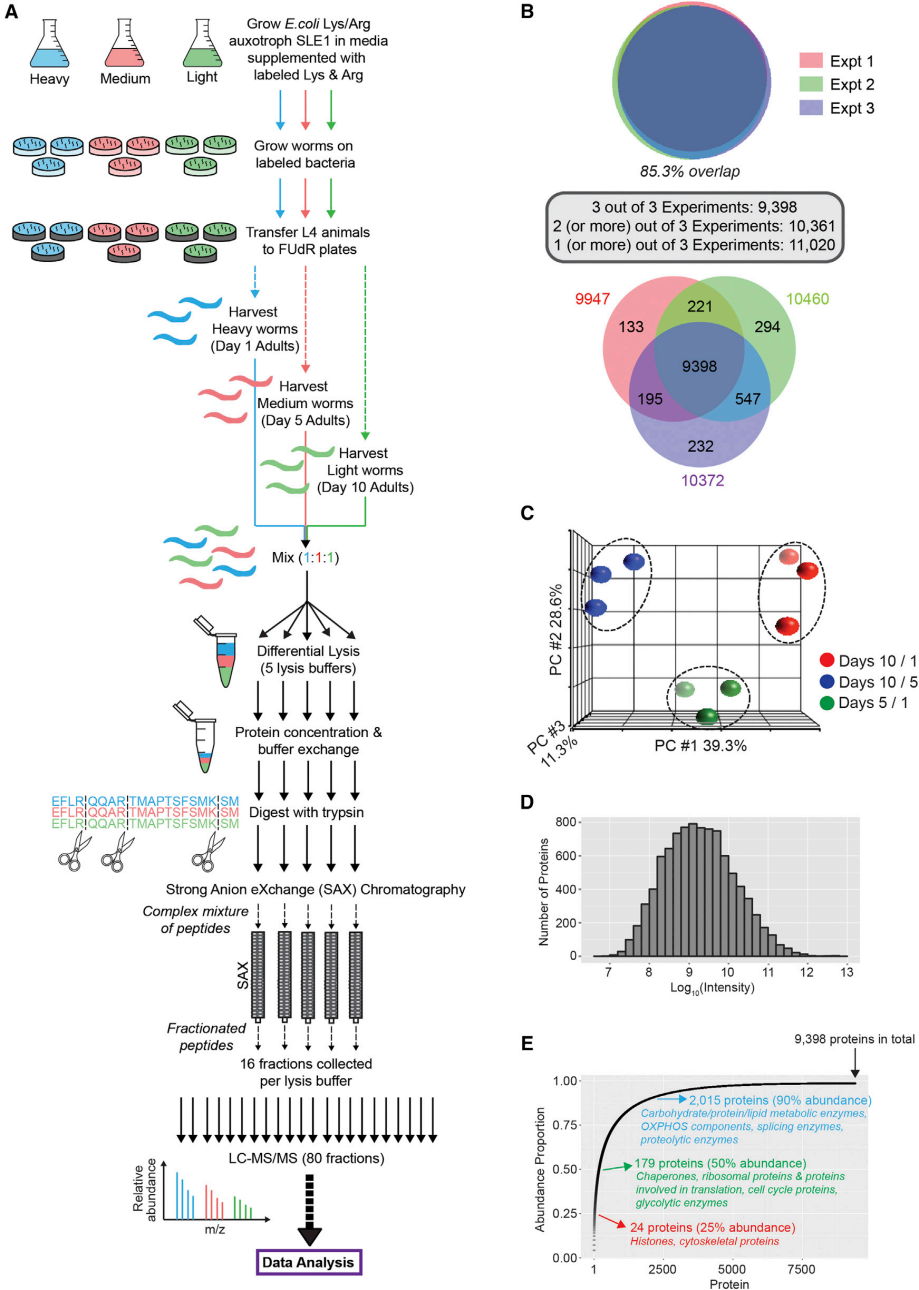
SILAC-Based Deep Proteome Analysis Reproducibly Identifies 9,398 Proteins in *C. elegans* (A) Schematic overview shows the methodology used in this study. (B) Area-proportionate Venn diagram (top) shows reproducibility in protein identifications across three biological replicates, with the number of proteins in each region of the Venn diagram indicated on a schematic (bottom). (C) Principal-component analysis (PCA) plot shows reproducibility in protein quantification across the three biological replicates. (D) Histogram shows log_10_-transformed protein abundance (MaxQuant intensity) for day 1 measurements. (E) A cumulative plot of protein abundance, as estimated using median protein intensity measurements based on three biological replicates. 9,398 proteins were identified in total with at least one peptide per protein. 90% of the bulk protein mass is made up of only 2,015 proteins (21% of measured proteins). The remaining protein identifications (7,383 or 79%) comprise less than 10% of the bulk protein mass. See also Figures S1–S4 and S8.

To make global measurements of age-related changes in protein abundance, we first developed a workflow to improve proteome coverage in *C. elegans*, while ensuring accurate quantification. We used the recently described SILAC-for-nematodes strategy, in which worms are fed bacteria whose proteins are labeled with arginine and lysine containing either light, medium, or heavy isotopes, resulting in near-complete labeling of the worm proteome after two generations (Larance et al., 2011, Fredens et al., 2011). This technique also exploits an RNAi-via-feeding strategy (Timmons and Fire, 1998) to knock down *orn*-*1*, thus preventing ORN-1-catalyzed arginine-to-proline conversion (Larance et al., 2011). We confirmed that *orn*-*1* RNAi had no significant effect on *C. elegans* lifespan (Figure S1A). Stable-isotope-labeled nematodes were harvested at days 1 (fertile young adults), 5 (early aging/post-reproductive), and 10 (aging) of adulthood (Figures 1A and S1B). Time points after day 10 of adulthood were not considered because *C. elegans* aging has a stochastic component that becomes particularly evident after this time (Herndon et al., 2002; Figure S1C). Importantly, although wild-type worms can live for up to 4 weeks, many phenotypes associated with aging, such as decreased rates of pharyngeal pumping, mitochondrial fission, and muscle deterioration, are visible at day 10 (Jiang et al., 2015, Croll et al., 1977, Huang et al., 2004, Garigan et al., 2002; Figure S1D).

As this study includes time points at which worms are fertile, in order to eliminate progeny from our assay, labeled worms were treated with 50 μM 5-Fluoro-2′-deoxyuridine (FUdR) at the mid-late L4 larval stage. We found that 100% of the progeny from worms treated with 50 μM FUdR were unviable, and that treated worms also had fewer eggs, thus decreasing egg-protein contamination in the assay (Figure S1E). Notably, unlike animals treated with higher doses of FUdR, at the chosen dose, animals were healthy, not prone to bursting, and, importantly, had a lifespan similar to that of untreated control animals (Figure S1F). The FUdR-treated labeled worms that were harvested at days 1, 5, and 10 of adulthood were next mixed and lysed in different buffers to increase proteome coverage (Figure 1A). Following digestion with trypsin, the resulting peptides were fractionated by hydrophilic strong anion exchange (SAX) chromatography to further reduce sample complexity, as described previously (Ly et al., 2014), prior to analysis by liquid chromatography-tandem mass spectrometry (LC-MS/MS). Three independent biological replicates were analyzed.

We used a differential buffer extraction methodology (QProteome Cell Compartment Kit, QIAGEN), which allowed us to sample a wider pool of proteins with different physicochemical properties (Figure S2A; Pourkarimi et al., 2012). This method also increased the number of unique peptides we identified from a single protein when the data were aggregated, thereby improving sequence coverage (Figure S2B). Additionally, we found that, although the QProteome kit was designed to work primarily with cells grown in culture, with our optimizations, the kit could be used successfully to isolate proteins from different sub-cellular compartments in day 1 adult worms, in spite of the presence of a cuticle and syncytium (Figures S2C–S2E). The validity of our method was supported by the predicted fractionation of proteins that previously have been shown to localize to specific cellular locations (Figures S2D and S2E).

Using this method, we compiled a resource with putative sub-cellular localization profiles for ∼6,300 *C. elegans* proteins, based on our proteomic analysis of day 1 adult nematodes treated with FUdR (Table S1). The relative proportions of these proteins in four sub-cellular compartments—i.e., cytoplasm, membrane (plasma membrane and membrane-bound organelles, excluding the nucleus), nucleus, and cytoskeleton/insoluble fractions—are presented, together with lists of proteins that were predominantly localized to any of these fractions based on our proteomic analysis (Table S1). This is a significant improvement over the 4,954 protein-coding nematode genes (of ∼20,000) that currently have a manually curated gene ontology (GO) term cellular component annotation, of which only a small subset (∼2,200 genes) is based on experimental evidence (Figures S2F–S2H; GO annotations and associated data were obtained from WormBase). As worm tissue integrity is compromised during aging (Garigan et al., 2002) and we were unable to confirm the efficacy of the kit in isolating sub-cellular fractions from old nematodes, we were hesitant to make any conclusions about changes in protein localization occurring during aging based on these data.

Using this combination of protein-level and peptide-level fractionation, >125,000 sequence-unique peptides were identified with a false discovery rate (FDR) of 1%. These were assembled into >9,300 protein groups that were detected in all three biological replicates (see the Supplemental Experimental Procedures; Table S2; Figure S3). Identifications were highly reproducible, with 10,361 protein groups identified in at least two of three biological replicates and 9,398 protein groups identified across all three biological replicates (Figure 1B). Relative protein quantification among the three biological replicates also was very reproducible, as shown in the principal-component analysis (PCA) plot (Figure 1C) and using Pearson’s correlation (median value for Pearson’s r across the various pairwise comparisons was 0.85; Figure S4A). The measured protein intensities spanned approximately seven orders of magnitude (Figure 1D), and mean protein sequence coverage was ∼30%.

Interestingly, the abundance composition of the *C. elegans* proteome is dominated by a relatively small proportion of proteins (Figure 1E; 24 proteins make up 25% of the measured abundance or total protein content), with apparent saturation at ∼4,000 proteins. Protein- and peptide-level fractionation enabled access to a much deeper proportion of the proteome, i.e., ∼7,383 proteins that cumulatively represent only 10% of the measured abundance. The total of 11,020 protein groups identified in at least one of the three biological replicates (see Figure 1B) corresponds to ∼54% of the predicted *C. elegans* proteome as defined by WormBase (Release WS243, March 28, 2014, containing 20,480 protein-coding genes; Figure S4B). Of the 11,020 protein groups, 6,480 proteins were reproducibly identified with sequence-unique peptides (Figure S4B). Where peptides could not be assigned to a single protein unambiguously (e.g., alternatively spliced isoforms and proteins encoded by genes with high sequence similarity), the lead member of the assembled protein group, as determined by Occam’s razor rule using MaxQuant (Cox and Mann, 2008), was used for all downstream data analysis.

Our dataset corresponds to ∼22% of the theoretical *C. elegans* MS-flyable peptide digest of the predicted proteome (Figure S4B). However, both the 54% overlap of our dataset with the theoretical proteome and 22% overlap with the theoretical peptide-digest are likely to be underestimated, as our study only used adult *C. elegans*. Indeed, a recent study identified only ∼3,000 proteins in young-adult nematodes (compared to ∼10,000 in our study) but ∼9,000 when developmental stages (embryo, L1, L2, L3, early L4, late L4, and young adult) were included (Grün et al., 2014). Similarly, ∼11,000 proteins were identified from a mixed-developmental stage worm population in an independent study (Schrimpf et al., 2009), although the study additionally reported a very high FDR (∼7%), a large number of single-peptide-based protein identifications (∼23% of the dataset), and an absence of biological replicates.

### Of the Adult *C. elegans* Proteome, ∼8% Changes Strikingly during Aging

**Figure 2:**
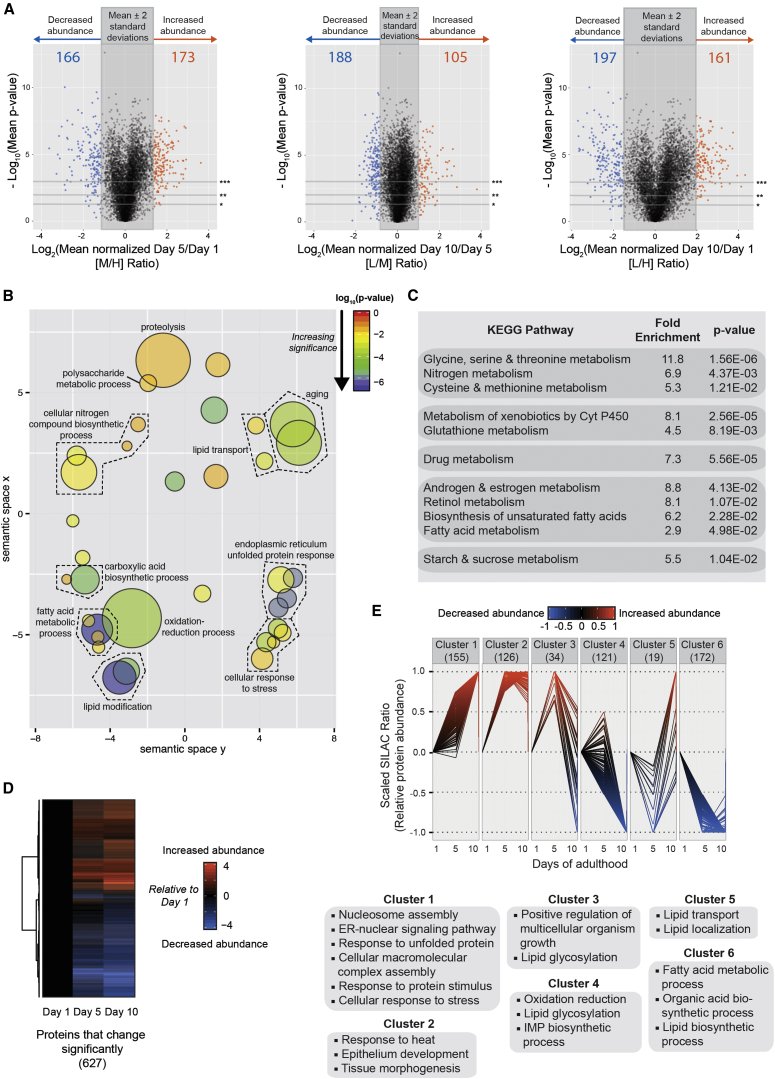
Of the Detected *C. elegans* Proteome, 8.5% Changes by a Large Magnitude during Aging (A) Volcano plot of log_2_-transformed SILAC ratios (fold change) against the negative log_10_ of the FDR-corrected p value calculated using ANOVA. Shown are the graphs for day 5 versus day 1 fold changes (left), day 10 versus day 5 (center), and day 10 versus day 1 (right). The shaded dark gray box represents the mean ± 1.96 SD for each plot. Horizontal gray lines denote p value cutoffs of 0.05 (^∗^), 0.01 (^∗∗^), and 0.001 (^∗∗∗^). The number of proteins that increase (orange) or decrease (blue) significantly (p value < 0.05) in each graph also is indicated. (B) GO term enrichment (biological process) of the 627 age-variant proteins (blue and orange dots from the three volcano plots in A with p values < 0.05) performed using DAVID and plotted using REVIGO. The size of the bubbles is indicative of the number of proteins annotated with that GO term; bubbles are color coded according to significance. (C) KEGG pathway enrichment of the 627 age-variant proteins calculated using DAVID is shown. (D) Heatmap shows age-dependent changes in abundance of the above proteins generated using R. (E) Hierarchical clustering of the 627 proteins into six groups based on trend profiles. Results from GO term enrichment analysis (p < 0.05) of each cluster using DAVID also are depicted. Only GO terms in clusters 1, 4, 5, and 6 remained significant (p < 0.05) after multiple hypothesis correction (Benjamini). See also Figure S4.

Of the 9,398 protein groups identified in all three biological replicates, those with at least two sequence-unique peptides in each replicate (7,380) were quantified across the day 1, 5, and 10 time points (Figure S3; Table S3). The 95% confidence intervals were defined for each SILAC pair (i.e., day 5/day 1 [M/H] ratio, day 10/day 5 [L/M] ratio, and day 10/day 1 [L/H] ratio) by calculating mean ratio ±2 SDs across the dataset. To compute a statistic for data reproducibility and significance, p values were calculated using ANOVA. Age-invariant control proteins with log_2_ SILAC pair ratios close to 0 were randomly sampled from the dataset for the ANOVA calculation. Additionally, as measurement error correlates inversely with intensity, the random sampling of controls was controlled for intensity (see the Supplemental Experimental Procedures and Figure S3 for details). Volcano plots show the FDR-corrected p value from ANOVA for each protein group against the respective log_2_(fold change) for each SILAC pair (Figure 2A). The p values less than 0.05 (95% cutoff) were considered significant, and 627 proteins were found to be significantly altered in abundance across the three time points considered (Table S4). The vast majority of these age-altered proteins had p values < 0.001.

### Specific Metabolic Processes and Stress Responses Are Preferentially Affected during Aging

GO term analysis for biological process was performed on the 627 proteins showing the most significant change in abundance, during the three time points measured, to profile nematode aging, using the Database for Annotation, Visualization and Integrated Discovery (DAVID; Huang et al., 2009) and ReviGo (Supek et al., 2011). This analysis revealed an enrichment of GO terms including aging itself, fatty acid metabolic processes, oxidation-reduction processes, unfolded protein response, and cellular response to stress (Figure 2B). Additionally, a highly significant enrichment of the GO term lipid modification also was observed, together with lipid transport and metabolic processes such as polysaccharide metabolism and cellular nitrogen compound biosynthesis (Figure 2B). Similar results were obtained upon analyzing the same 627 proteins for Kyoto Encyclopedia of Genes and Genomes (KEGG) pathway enrichment (Kanehisa and Goto, 2000, Kanehisa et al., 2014) using DAVID. Thus, enrichment of cellular metabolic pathways, including amino acid metabolism, fatty acid metabolism, and carbohydrate metabolism, was observed (Figure 2C), along with cellular stress responses. As GO term enrichment analysis does not take into account the direction of changes (i.e., whether a network is up- or downregulated), we further probed our dataset using gene set enrichment analysis (GSEA) as described below.

We generated a heatmap of the 627 proteins whose levels showed the most significant change across the three time points examined to assess whether groups of proteins show similar expression profiles during aging (Figure 2D). The heatmap groups proteins that showed similar abundance changes during adulthood, which were then further clustered using an unbiased hierarchical clustering approach. To minimize redundancy in clustering due to large differences in the magnitude of the abundance changes, the SILAC ratios were scaled to range between −1 (maximum decrease) and +1 (maximum increase), with ratios near 0 indicating no change in abundance with age. Using hierarchical clustering based on Ward’s minimum variance method (Ward, 2012), we grouped the age-variant proteins into clusters based on their abundance trend profiles.

Although two clusters (proteins that increase with age and proteins that decrease with age; Figures S4C and S4D) may be used to categorize this set of 627 proteins, we found that grouping the proteins into six clusters could be more biologically meaningful, although one must be cautious in interpreting these data as only three time points were considered in this study (Figures 2E, S4C, and S4D). For example, cluster 3 in the six-cluster model, which includes 34 proteins that increase sharply in abundance between days 1 and 5 of adulthood and then decrease sharply between days 5 and 10, is enriched for the GO term lipid glycosylation (Figure 2E). Thus, it will be interesting to carry out follow-up biological experiments to examine whether lipid glycosylation or other lipid modifications decrease during aging and to study the functional consequences of this decrease. In the two-cluster model, this small group of proteins is grouped together with a larger group of proteins whose levels increase during days 1–5 and then continue to increase between days 5 and 10, and the GO term lipid glycosylation is not significantly enriched (Figure S4D).

We next extended our analysis to include our entire aging dataset, rather than just the subset of 627 proteins that showed the largest fold change during the three time points, using GSEA (Subramanian et al., 2005). The advantage of this type of analysis is that, in addition to genes (or proteins) associated with gross changes in abundance that are usually defined by arbitrary fold-change cutoffs, GSEA also detects small but reproducible changes in levels of RNAs or proteins, provided a similar trend is observed in multiple constituents of a defined gene set or pathway. Because GSEA, in common with the majority of pathway analysis tools, was designed to work primarily with human genes rather than nematode genes, we first probed the dataset using two nematode gene sets that we compiled, using information from WormBook, WormBase, and the literature (Kenyon, 2010, Murphy and Hu, 2013, Lapierre and Hansen, 2012), for changes in networks known to be associated with aging in *C. elegans*. We could reproducibly detect and quantify known *C. elegans* components of the TORC1 and TORC2 complexes and 17 components of the insulin pathway involved in aging, notable exceptions being IST-1, SKN-1, and HSB-1.

**Figure 3:**
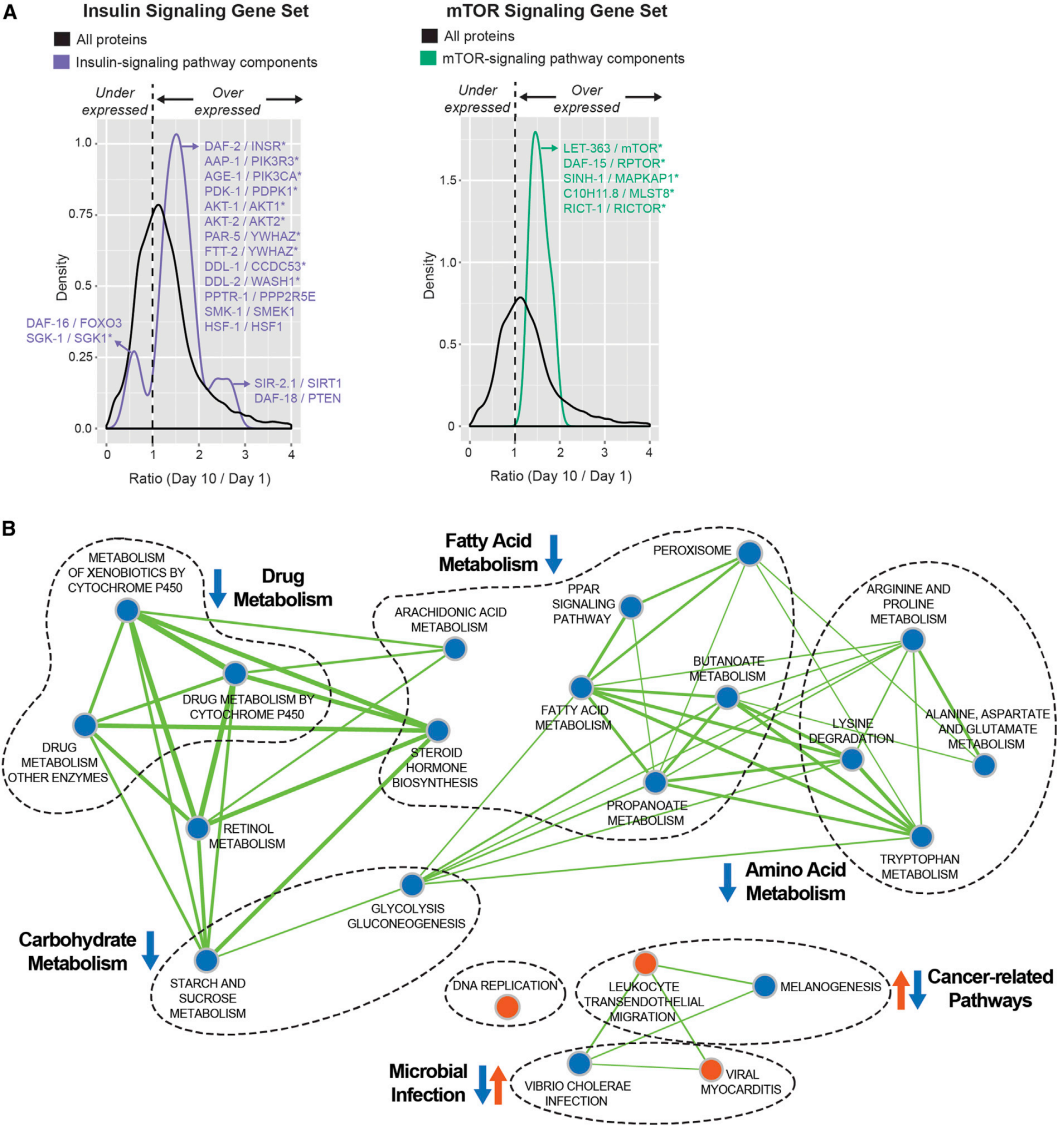
GSEA Uncovers a Downregulation of Cellular Metabolic Pathways with Age (A) Density plots depicting the protein abundance of self-defined gene sets (left, insulin-IGF signaling; right, mTOR) relative to the entire dataset collected in this study. *C. elegans* proteins and their human orthologs are indicated in the figure. Negative regulators, in the context of extended longevity, are annotated with an asterisk (^∗^). (B) GSEA (KEGG pathways) output of the day 10 versus day 5 changes in protein abundance depicted schematically using Enrichment Map (Cytoscape). The thickness of the lines connecting nodes (i.e., KEGG pathways) indicates the overlap of quantified proteins common to both nodes. See also Figure S5.

As shown by density plots (Figure 3A, left panel), nearly all proteins in the insulin-signaling gene set showed a consistent, albeit modest, increase in abundance between days 1 and 10 of adulthood when compared to the total quantified proteome, suggesting the hypothesis that the activity of this pathway late in life accelerates aging. The exceptions were the longevity-promoting transcription factor DAF-16 (FOXO3 in humans) and the serine/threonine protein kinase SGK-1 (SGK1), which decreased in abundance during aging. Since DAF-16 is negatively regulated by insulin/IGF-1 signaling, its downregulation makes sense; however, SGK-1, which was identified with at least ten razor/unique peptides in each of the three biological replicates (∼31% sequence coverage), seems to be a true biological exception. The increase in abundance of the proteins SIR-2.1 (SIRT1) and DAF-18 (PTEN) was more striking than other components of the insulin-signaling gene set. These appear to be biological exceptions, as both proteins are positive regulators of longevity (Kaeberlein et al., 1999, Masse et al., 2005, Schmeisser et al., 2013). At least 9 razor/unique peptides from SIR-2.1 were detected in each biological replicate, and the sequence coverage was ∼29%, while >26 razor/unique peptides from DAF-18 were detected with sequence coverage of ∼49%. A similar trend was observed when the mTOR gene set we compiled was examined, consistent with the literature (Chen et al., 2009, Perkey et al., 2013), with an increase in TORC1 and TORC2 components observed between days 1 and 10 of adulthood (Figure 3A, right panel).

Having extracted information about known aging pathways from our dataset, we next performed GSEA for all pathways in the KEGG database using human homologs of the *C. elegans* proteins quantified in this study. Of the *C. elegans* protein-coding genes from our proteomic dataset, 4,394 had known human homologs (see the Supplemental Experimental Procedures), with multiple worm genes mapping to a single human homolog in many cases. Consequently, this list comprises 3,067 unique human homologs. To help exclude effects contributing to reproduction rather than organismal aging (Figure S1B), we focused on post-reproductive changes in protein abundance occurring between days 5 and 10 of adulthood. GSEA comparing changes in protein levels in day 5 versus day 10 animals confirmed an enrichment of pathways that also were enriched in the analysis of the subset of 627 proteins whose levels showed the largest change during aging (Figures 2B and 2C). As GSEA also takes into account the direction of the change in abundance, we observed that there is a marked downregulation of proteins involved in cellular metabolic pathways during aging. For example, enzymes involved in carbohydrate, amino acid, fatty acid, as well as drug and retinol metabolism are all decreased between days 5 and 10 of adulthood (Figures 3B and S5A).

### GSEA Uncovers an Age-Dependent Decline in Peroxisomal Protein Import

**Figure 4:**
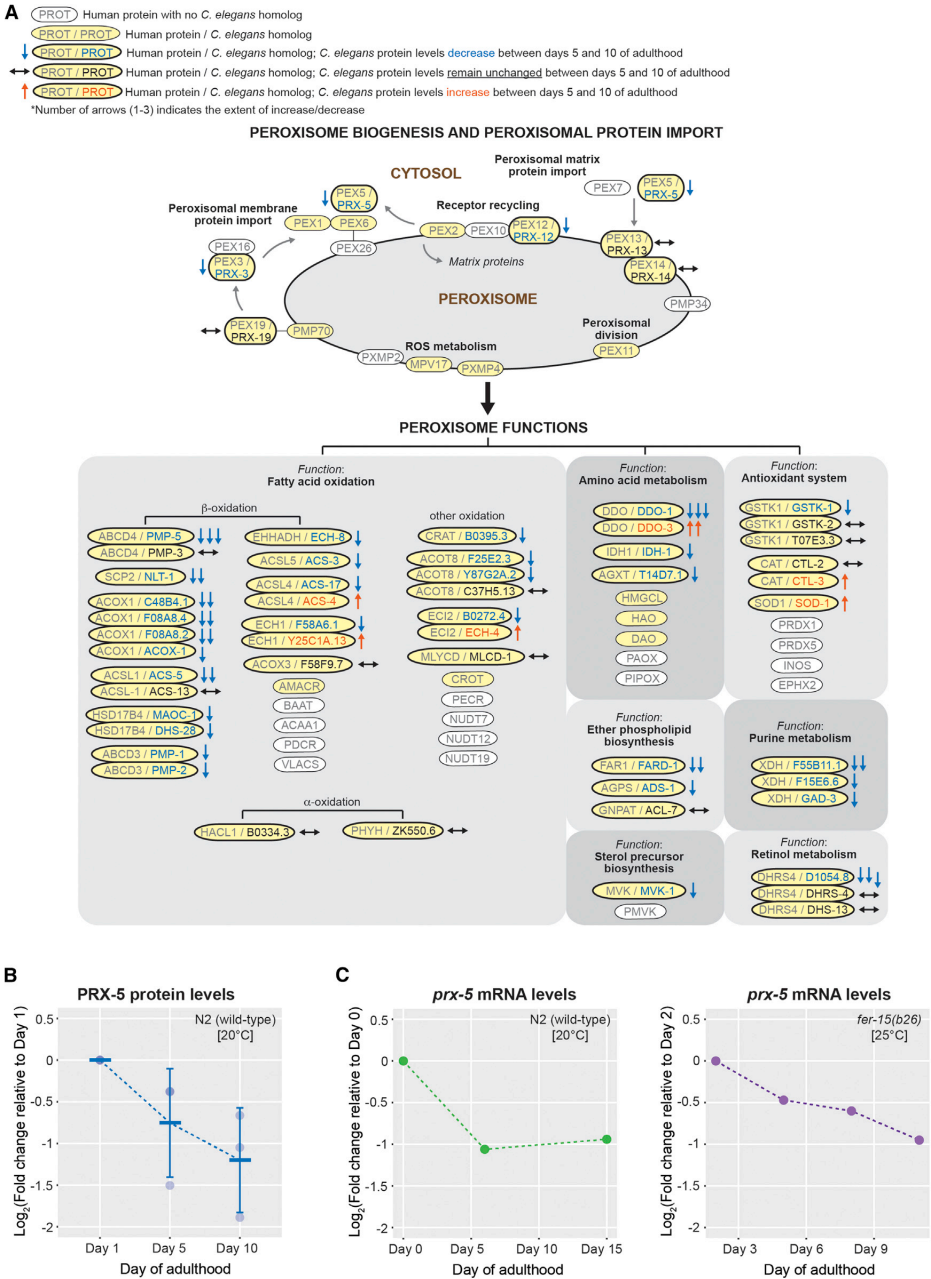
Levels of the Peroxisome Import Protein PRX-5 Decrease during Aging (A) Schematic depicting an adaptation of the KEGG pathway peroxisome in humans (adapted from www.genome.jp/kegg/). *C. elegans* homologs of the human proteins are shaded yellow. Also indicated are the proteins quantified in this dataset, with orange, blue, or black arrows to denote whether the proteins were found to increase or decrease in abundance or to remain relatively unchanged between days 5 and 10 of adulthood, as measured in our study. (B) Graph shows the decrease in levels of the peroxisomal import protein PRX-5 during aging, at the indicated time points. (C) Graph shows the decrease in mRNA levels of *prx*-*5* during aging observed in previously published datasets in wild-type nematodes (left; Youngman et al., 2011) and temperature-sensitive sterile mutants (right; Budovskaya et al., 2008). See also Figure S5.

In addition to age-dependent decreases in cellular metabolism, GSEA also showed a downregulation of proteins functioning in the KEGG pathway peroxisome (Figure 3B). A closer look revealed a decrease in abundance of ∼30 proteins involved in peroxisomal protein import and function. For example, PEX3 (human ortholog)/PRX-3 (*C. elegans*), involved in the import of peroxisome membrane proteins, decreased in abundance during aging (Figure 4A), as did levels of PEX5/PRX-5, a protein known to be involved in the import of peroxisomal-matrix enzymes (Figures 4A and 4B; Petriv et al., 2002). Additionally, peroxisomal proteins involved in fatty acid oxidation, ether phospholipid biosynthesis, sterol precursor biosynthesis, amino acid metabolism, purine metabolism, and retinol metabolism decreased in abundance between days 5 and 10 of adulthood (Figure 4A).

The peroxisomal import protein PRX-5 has not been identified previously in proteomic studies on aging in wild-type nematodes, probably because of a limited depth of proteome coverage. When we mined existing microarray data of *C. elegans* aging (Youngman et al., 2011, Budovskaya et al., 2008), we found that *prx*-*5* mRNA levels decreased during aging in two independent studies using wild-type nematodes as well as temperature-sensitive sterile mutants (Figure 4C, left and right panels, respectively). Interestingly, analysis of mRNA transcript levels from published data (Youngman et al., 2011) for all the peroxisome proteins identified in our proteomic study revealed that, unlike *prx*-*5* transcript levels, the majority of mRNAs encoding peroxisome proteins were present at similar levels to the overall mRNA population, i.e., the entire dataset (Figure S5B, upper panel). This is in contrast with peroxisome protein levels measured in our proteomic study, the majority of which decreased in abundance when compared with the overall protein population (Figure S5B, lower panel). Examination of global mRNA levels (not just for the subset of peroxisome transcripts; Youngman et al., 2011) and our protein data showed a poor correlation (r = 0.25 [Pearson]; Figure S5C), consistent with previous findings (Walther et al., 2015).

**Figure 5:**
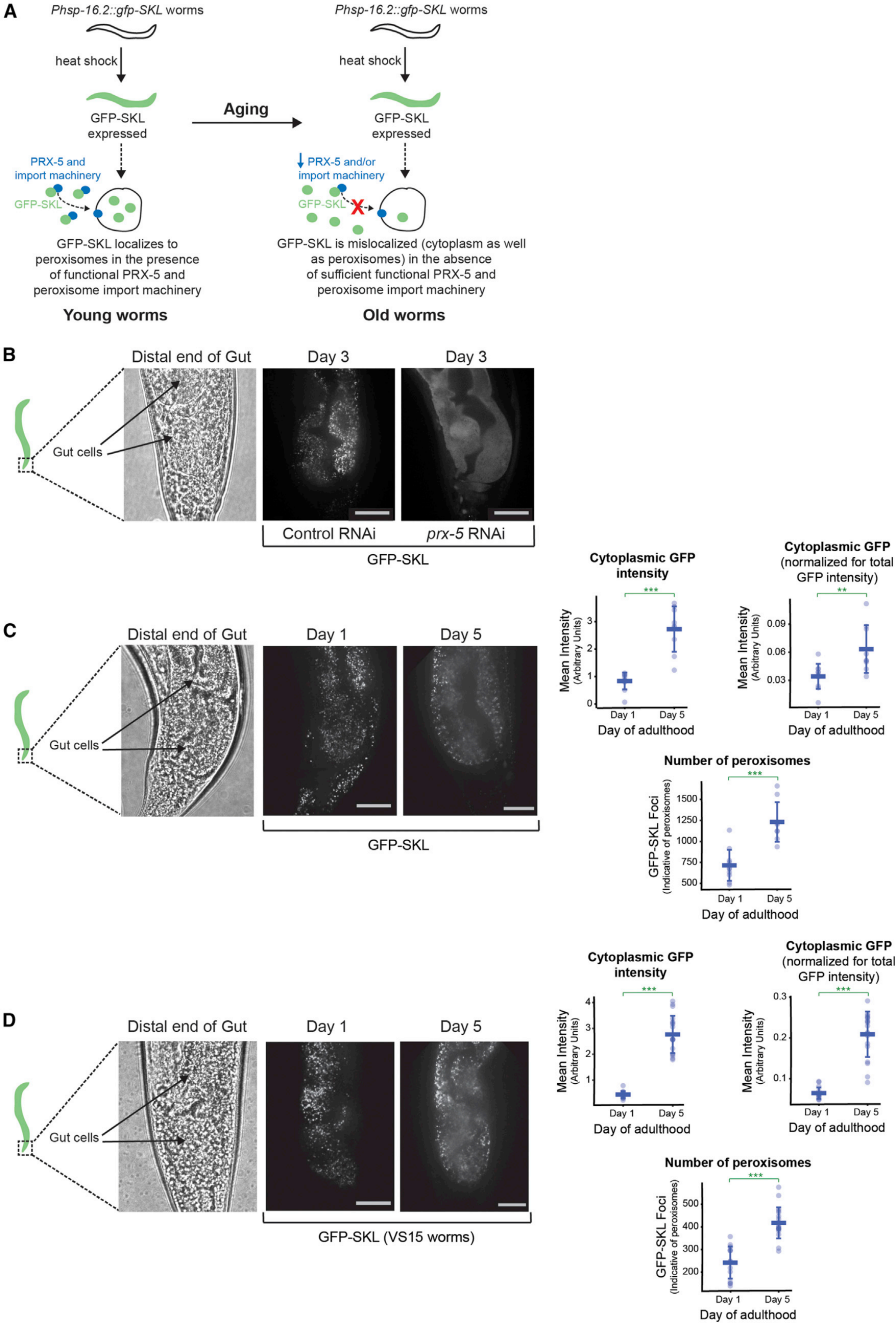
Peroxisomal Protein Import Is Impaired during Aging (A) Schematic depicting the correct peroxisomal localization of GFP targeted to the peroxisome (GFP-SKL) in young animals (left panel). If, as predicted by our MS analysis, the peroxisome import machinery is compromised during aging, GFP-SKL import into the peroxisome will be impaired (right panel). (B–D) Scale bar, 10 μm. (B) GFP-SKL is correctly localized to the peroxisome in the intestine of control animals, but mislocalized to the cytosol under conditions where *prx*-*5* is knocked down with RNAi (n = 7). (C) GFP-SKL begins to accumulate in the cytoplasm of aging animals (day 5 adults) when compared to day 1 adults. Shown are representative images from the distal intestinal cells. Cytoplasmic (diffuse) GFP intensity was quantified, summed, and plotted from 81 individual z-sections at 0.5-μm intervals for each worm to minimize masking of the cytoplasmic signal by intense GFP foci (peroxisomes) as well as to minimize bleed through. The cytoplasmic signal was found to increase significantly with age (upper left graph; p value calculated using an unpaired t test = 7.68 × 10^−5^; n = 11 individuals for day 1 adults and n = 9 for day 5 animals). As the total *hsp*-*16.2*-driven GFP-SKL signal also was found to increase with age, cytoplasmic GFP measurements were corrected for total intensity in each image and age-dependent changes remained significant (upper right graph; p value from unpaired t test = 0.009). Also indicated is a count of the number of dots resolved (peroxisomes) at days 1 and 5 from a single z-section. This was found to increase significantly by day 5 (lower graph; p value from unpaired t test = 7.86 × 10^−5^). (D) As above, but we used worms that express GFP-SKL under the intestine-specific *ges*-*1* promoter (strain VS15). Cytoplasmic GFP-SKL intensity increased significantly with age (p value from unpaired t test = 1.56 × 10^−11^ for raw cytosolic intensities [left graph] and 4.54 × 10^−10^ for cytosolic GFP intensity normalized to total GFP intensity [right graph]; n = 15 for day 1 adults and n = 19 for day 5 adults). As in (C), the number of dots resolved also increased significantly with age (lower graph; p value from unpaired t test = 4.46 × 10^−8^). See also Figure S6.

To validate our proteomic data and assess whether a gradual failure of the peroxisomal import machinery occurs during aging (Figure 5A), we made use of a *C. elegans* peroxisome reporter in which GFP is linked to the peroxisome-targeting sequence (PTS1; GFP-SKL) and expressed using an *hsp*-*16.2* heat shock promoter (Thieringer et al., 2003). We verified that GFP-SKL localized correctly to foci indicative of peroxisomes (Thieringer et al., 2003) in the presence of functional import machinery (Figure 5B, middle panel). As predicted, when animals were treated with *prx*-*5* RNAi during adulthood, GFP failed to localize to the peroxisome and instead showed a diffuse cytoplasmic distribution (Figure 5B, right panel; Thieringer et al., 2003). To assess whether the foci were artifacts due to heat stress, we tested and confirmed that GFP localization without the PTS1 target sequence in *Phsp*-*16.2*::*gfp* animals was diffuse and devoid of distinct foci (Figure S6A).

Next we asked whether GFP-SKL was similarly mislocalized to the cytoplasm (see Figure 5B) during aging, as predicted from our MS analysis. Day 5 adult worms showed a mixed distribution of GFP-SKL between peroxisomes, indicated by intense spots/foci, and the cytoplasm (diffuse signal), whereas young day 1 animals showed GFP-SKL localized predominantly in peroxisomes (Figure 5C). Quantification of the cytoplasmic GFP signal confirmed an increase by day 5 relative to day 1 (Figure 5C, top left graph). However, as total GFP levels produced using the *hsp*-*16.2* promoter increased by day 5 (Figures S6B and S6C), we normalized the cytoplasmic GFP signal to total GFP intensity. After normalization, cytoplasmic GFP levels were still found to be significantly higher in day 5 adults compared with day 1 adults (Figure 5C, top right graph). Time points beyond day 5 were not considered, as GFP-SKL expression driven by the *hsp*-*16.2* promoter was markedly reduced by day 10 (Figures S6B and S6C).

Interestingly, the number of resolved foci (peroxisomes) increased significantly between days 1 and 5 of adulthood (Figures 5C, lower graph, and S6D). A similar observation has been made in senescent human fibroblasts (Legakis et al., 2002), where the authors speculate that this could be due to an age-dependent impairment in the signals that lead to peroxisome growth and division or, alternately, a failure to remove these structures by autophagy. The cytoplasmic mislocalization of GFP-SKL in aging animals was verified using a second strain, in which expression was controlled by the intestine-specific *ges*-*1* promoter (Figure 5D). Thus, we conclude that post-reproductive *C. elegans* hermaphrodites mislocalize proteins that are normally targeted to the peroxisome in young adults, likely due to a dysfunction of the peroxisome import machinery.

### Reducing Insulin/IGF-1 Signaling Slows the Rate of Biological Aging without Slowing the Rate of Change of Much of the Age-Dependent Proteome

**Figure 6:**
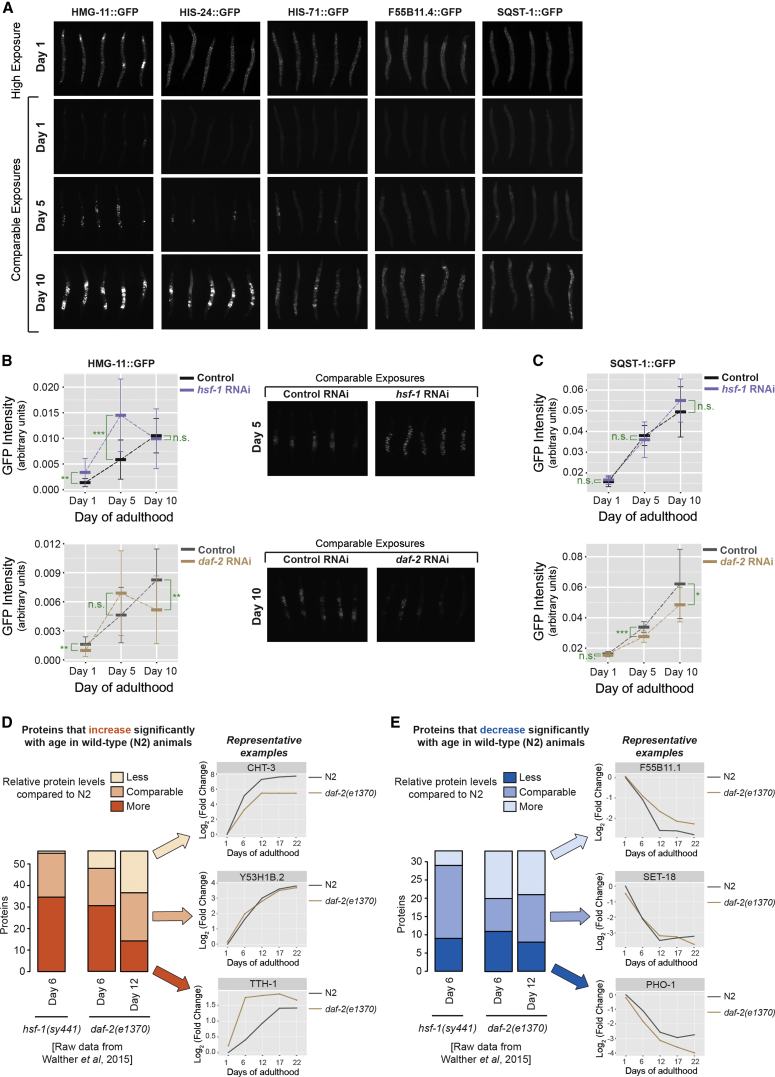
Most Age-Variant Proteins Do Not Scale with Biological Age in Long-Lived Insulin/IGF-1 Mutants (A) Snapshots of GFP fluorescence at days 1, 5, and 10 of adulthood for the indicated GFP::protein fusions. Shown are high exposures at day 1 (upper panels) and comparable exposures at days 1, 5, and 10 (lower panels) (n = 20 per experiment). (B) HMG-11::GFP-expressing worms were grown on the indicated RNAi-bacteria (initiated at L1 stage) and GFP fluorescence was quantified on days 1, 5, and 10 of adulthood. Graphs show mean ± SD (n = 20 worms). Significance was calculated using an unpaired t test (^∗^p < 0.05, ^∗∗^p < 0.01, ^∗∗∗^p < 0.001; n.s., not significant). The *daf*-*2* and *hsf*-*1* dsRNA constructs are in different vector backbones, hence the corresponding empty vector control for each was used. Snapshots of images taken at day 5 (*hsf*-*1* RNAi) and day 10 (*daf*-*2* RNAi) also are shown. (C) As in (B), except we used SQST-1::GFP-expressing hermaphrodites (n = 20 except for day 10 *hsf*-*1* RNAi where n = 11). (D and E) 88 proteins from the 627 age-variant proteins identified in this study were detected in a previous study by Walther et al. (2015) in wild-type, *daf*-*2*(*e1370*), and *hsf*-*1*(*sy441*) animals, and they were found to show similar abundance trends in wild-type animals when compared to our dataset. Of these, those that increased with age in wild-type animals also were quantified in *daf*-*2*(*e1370*) and *hsf*-*1*(*sy441*) worms (D). The stacked bar plot shows how the levels of these proteins vary in the different strains relative to wild-type animals at day 6 (*hsf*-*1*) or days 6 and 12 (*daf*-*2*) of adulthood, using raw data from Walther et al. (2015). Also indicated are representative examples of proteins present at lower, comparable, or higher levels in day 12 *daf*-*2* mutants compared to wild-type controls. (E) As in (D), except that proteins whose levels were found to decrease in wild-type animals were analyzed. As in (D), raw data from Walther et al. (2015) were used to generate the bar plots. See also Figure S7.

Long-lived insulin/IGF-1-pathway mutants exhibit reduced rates of tissue and behavioral aging. This raises the question of whether proteins that change in abundance with age in wild-type animals (Figure 2) show the expected slower rate of change when lifespan is extended. To test this, we first confirmed, in vivo, the age-dependent change in levels of several proteins whose abundance increased with age in our MS analysis, using GFP::protein-fusion worm strains (Figures 6A and S7A–S7G). Specifically, levels of GFP::fusions to either the DNA-binding protein HMG-11, the linker histone HIS-24, the histone H3 variant HIS-71, a nematode-specific protein (F55B11.4), or the p62/sequestosome homolog SQST-1, an indicator of autophagic flux, all increased during the course of aging in vivo (Figure 6A).

Next we asked how these proteins behaved in lifespan mutants. When HMG-11::GFP worms were raised under life-extending conditions (*daf*-*2* RNAi), HMG-11::GFP protein levels were significantly lower (younger pattern) on day 10 of adulthood, relative to control. Conversely, under conditions that accelerate aging and shorten lifespan (*hsf*-*1* RNAi) (Garigan et al., 2002), HMG-11::GFP protein levels were higher (older pattern) on day 5 relative to control. Thus, HMG-11 protein accumulation scales with the overall apparent rate of aging in these cases (Figure 6B). However, surprisingly, this was not the case for any of the remaining proteins. SQST-1::GFP levels were significantly lower in *daf*-*2*(*RNAi*) animals, as predicted, but they were not higher in *hsf*-*1*(*RNAi*) animals (Figure 6C). HIS-24::GFP, HIS-71::GFP, and F55B11.4::GFP protein levels were not lowered in *daf*-*2*(*RNAi*) worms; in fact, the levels of F55B11.4::GFP were higher (older pattern) in *daf*-*2*(*RNAi*) worms when compared with wild-type (Figure S7H). In *hsf*-*1*(*RNAi*) animals, in some cases (day 10 for HIS-24 and day 5 for F55B11.4), protein levels were lower (younger pattern) than in wild-type animals (Figure S7H).

These unexpected results prompted us to extend this analysis to include other proteins from the list of 627 proteins whose abundance changed most strikingly during aging. Of these 627 proteins, we concentrated on the 88 that also were identified in a recent study that analyzed the proteomes of wild-type (N2 Bristol), long-lived *daf*-*2*(*e1370*) mutants, and short-lived progeric *hsf*-*1*(*sy441*) mutant worms (Walther et al., 2015). Of the 88 proteins, 55 increased with age in wild-type animals in both the Walther et al. (2015) study and in our study, and 33 decreased with age. A closer look at the 55 proteins that increased with age revealed that 35% (19 proteins) accumulated to a lesser extent (more youthful pattern) in day 12 *daf*-*2*(*e1370*) adults relative to wild-type animals (Figure 6D). These included peptidases (F48E3.4 and T28H10.3), fatty acid-binding proteins (FAR-6 and LBP-2), the tumor necrosis factor-induced protein homolog T05E12.3, the carbohydrate-binding C-type lectin CLEC-146, transthyretin-related proteins (TTR-51, TTR-6, and TTR-30), the galectin LEC-2, which is believed to play a role in programmed cell death, the UDP glycosyltransferase UGT-31, and some nematode-specific proteins of unknown function.

Interestingly, a large proportion of the 55 age-increasing proteins (40%) did not show an appreciable change in levels between age-matched N2 and *daf*-*2* mutant animals. Further, 25% (14/55) of them were found to increase to a much greater extent (older pattern) in *daf*-*2*(*e1370*) mutants than in wild-type worms. In principle, these 14 proteins could function to delay aging, consistent with the findings of our lab and other labs that *daf*-*2* mutants express cell-protective mRNA species at high levels even in young adults (Murphy et al., 2003). In support of this hypothesis, we found that this subset of proteins contained the small heat shock protein HSP-12.3. However, this subset also included the aurora-related kinase AIR-2, which could be associated with the longer period of egg laying observed in *daf*-*2*(*e1370*) mutants (Gems et al., 1998). Other proteins in this subset included cytoskeletal and cuticle components (TBB-6, CHT-1, and TTH-1), the fatty acid-binding protein FAR-3, the nucleotide-binding protein CATP-3, and the enzymes HEX-1 (hexosaminidase) and F13H8.11 (phospholipase B1).

When the levels of the 55 proteins that increase with age in wild-type animals were measured in *hsf*-*1*(*sy441*) adults at day 6 (*hsf*-*1* mutants are mostly dead by day 12; hence, proteomic measurements during this period are likely to be biased), it was found that 62% (34/55) scaled with biological age, accumulating to a greater extent in these animals when compared with day 6 wild-type control animals (Figure 6D). However, 36% (20/55) of the proteins were unchanged between *hsf*-*1*(*sy441*) and control animals, and one protein of unknown function, T12D8.5, accumulated to a lesser extent in *hsf*-*1*(*sy441*) animals.

Similarly, we examined the 33 proteins from the pool of 627 age-variant proteins that were found to decrease during aging in wild-type animals in our study as well as in the Walther et al. (2015) study. Of these, 36% (12/33) scaled with biological age, decreasing to a lesser extent (i.e., the protein was more abundant, younger pattern) in long-lived *daf*-*2*(*e1370*) worms compared to wild-type worms at day 12 of adulthood (Figure 6E); 39% of the proteins remained unchanged between the two populations, and 24% (8/33) were present at lower levels (older pattern) in *daf*-*2*(*e1370*) mutants when compared to wild-type controls. In *hsf*-*1*(*sy441*) mutants at day 6 of adulthood, the levels of 27% (9/33) scaled with biological age, i.e., they were present at lower levels in these animals compared to age-matched controls (Figure 6E). However, the vast majority (61%) were unchanged between wild-type and *hsf*-*1*(*sy441*) worms, and 12% (4/33) were present at higher levels (younger pattern) in *hsf*-*1*(*sy441*) animals. Thus, the aging rate of much of the age-variant proteome, at least in terms of protein abundance, is unchanged under conditions where the rate of biological aging is altered by manipulating insulin/IGF signaling.

## Discussion

In this study, we present an in-depth measurement of age-related changes in protein abundance in *C. elegans*, facilitated by deep MS-based quantitative proteomic analysis that provides a major increase—almost a doubling—in coverage of the adult *C. elegans* proteome. Specifically, we have reproducibly identified >9,300 proteins in each of three biological replicates (and >11,000 in at least one of the three experiments), and we have monitored how these vary in abundance at three time points. The depth of proteome coverage obtained, together with the high degree of reproducibility among biological replicates, allowed the definition of strict statistical thresholds for assessing the significance of age-related protein abundance changes, rather than relying simply on arbitrary fold-change cutoffs. Using these stringent thresholds, we identified, in addition to factors previously shown to vary in abundance during aging in *C. elegans* (Liang et al., 2014, Walther et al., 2015, Zimmerman et al., 2015; see Figures S8A–S8F for comparisons between our study and previous proteomic studies), examples of proteins whose abundance variation and function have not previously been linked to aging.

Our dataset provides a valuable resource that can be used to generate hypotheses that will help to further our understanding of aging, which can then be tested in vivo. We provide two examples of this. First, our deep proteome analysis unveiled a decrease in the abundance of ∼30 peroxisomal proteins with age (Figure 4A). In particular, we observed a decrease in abundance of the peroxisomal import protein PRX-5 (PEX5 in humans; Figure 4B) during aging, and we were curious to examine whether this results in impaired peroxisomal protein import during aging. There are reports of an age-dependent mislocalization of catalase to the cytoplasm, and studies in middle- and late-passage human fibroblasts speculate that catalase mislocalization during replicative senescence may be attributed to impaired peroxisome import (Legakis et al., 2002). Whether this phenomenon also is observed during organismal aging and applies to all PTS1-containing peroxisomal proteins or only to catalase, which has a non-canonical PTS1 (Lys-Ala-Asn-Leu rather than Ser-Lys-Leu; Purdue et al., 1996), was still unclear. In this study, we used a peroxisome-targeted GFP-PTS1 reporter and showed, in vivo, that this protein was mislocalized during aging in two independent worm strains (Figures 5C and 5D). Thus, our data support a model wherein impaired peroxisome protein import caused by decreased PRX-5 levels leads to peroxisome dysfunction with age.

Like mitochondria, peroxisomes generate reactive oxygen species (ROS), and, although mitochondria are responsible for the majority of the ROS produced within a cell, ROS generated in peroxisomes appear to have a profound impact on mitochondria. Peroxisomal catalase deficiency disrupts mitochondrial redox balance (Hwang et al., 2012), and the induction of ROS production in the peroxisome results in increased mitochondrial fragmentation (a hallmark of aging) and an altered redox potential (Ivashchenko et al., 2011). Peroxisomes and mitochondria therefore appear to be intimately linked, and the interplay between the two organelles could be important in the context of aging. Although *prx*-*5* is essential for nematode larval development (Thieringer et al., 2003), surprisingly, when knocked down in adult nematodes, lifespan was not shortened but, if anything, increased slightly (Zhou et al., 2012, Curran and Ruvkun, 2007). In our hands, adult-only *prx*-*5* RNAi treatment resulted in a significant decrease in brood size (p = 0.00019; Figure S6E), raising the possibility that lifespan is increased in nematodes via the pathway that extends lifespan in response to germline depletion or potentially by a cell-protective response triggered by, for example, an altered redox potential. Interestingly, in yeast, although *Δpex5* cells show minimal peroxisome biogenesis defects, they have a dramatically shorter chronological lifespan when grown on 0.5% glucose (mean lifespan ∼7 versus ∼16 days in wild-type cells and maximum lifespan ∼10 versus ∼23 days), underscoring the importance of correctly localized peroxisome matrix proteins imported via functional Pex5p (PEX5/PRX-5) in maintaining normal lifespan (Lefevre et al., 2013).

A large compendium of proteins whose levels change with age is a powerful tool to better understand how single-gene mutations, such as *daf*-*2* insulin/IGF-1-receptor mutations, can slow aging and extend lifespan. *daf*-*2* mutants are known to express many cell-protective proteins at elevated levels, even as young adults (e.g., proteasome subunits and molecular chaperones [Depuydt et al., 2013, Walther et al., 2015, Walker et al., 2001] as well as some autophagy components [Hashimoto et al., 2009, Hansen et al., 2008]). To test whether abundances of age-variant proteins scale with the rate of biological aging, we examined the levels of some of the age-variant proteins identified in our study in vivo in *daf*-*2*(*RNAi*) worms. As expected, the accumulation rates of some proteins were slower in *daf*-*2*(*RNAi*) animals than in wild-type. However, surprisingly, this was not the case for all the proteins we examined. When this analysis was expanded to include proteomic data from other studies (Walther et al., 2015), we confirmed that only a minor fraction of the age-variant proteins identified in wild-type animals showed reduced rates of change in long-lived *daf*-*2* mutants. The majority of proteins accumulated at similar rates in chronologically age-matched wild-type animals versus *daf*-*2*(*e1370*) mutants. Stated simply, these proteins didn’t seem to know that they were in a long-lived mutant.

These results agree with our lab’s previous study of mRNA changes during early adulthood (days 1–6) in *daf*-*2*(−) animals (Murphy et al., 2003), where again, the age-specific abundances of many mRNAs changed at a wild-type rate. However, the significance of this was not clear because these mRNAs might have been linked to reproduction, which takes place during this period, involves two-thirds of the animal’s cells, and was known to be fairly normal in these mutants. Our finding that this observation also holds true for protein levels, and extends well beyond the reproductive phase of adulthood, is thus significant. It suggests that the aging rate of much of the age-variant proteome, at least in terms of protein abundance, is unchanged in these long-lived animals. More fundamentally, it implies that an important biological clock still ticks at a normal rate in these animals, in spite of the change in their rates of morphological decline. These mutants probably age more slowly at the organismal level and live long because the relatively small set of cell-protective proteins and proteostasis regulators, known to be expressed constitutively at high (or low) levels even in young *daf*-*2* adults (Murphy et al., 2003, Dong et al., 2007), alter the extent to which normal time-dependent processes affect biological aging.

In summary, here we describe a highly reproducible proteomic dataset containing >11,000 proteins identified in the adult worm (>9,300 reproducibly in three biological replicates), and we show how these proteins change during aging. Network analysis of our data, together with follow-up in vivo studies to test hypotheses generated using our dataset, has identified impairments in peroxisomal protein import during aging and, surprisingly, the finding that the majority of age-variant proteins do not scale with the rate of biological aging in long-lived insulin/IGF-1-receptor mutants. Additionally, this deep proteomic analysis contributes candidate determinants for biological aging (organismal and tissue decline) and a large set of proteins whose abundances, to our knowledge, were not previously shown to change with age, including proteins that may serve as biomarkers of aging and can be tested genetically for a causal role. To maximize the value of this resource to the community, all of these data are provided in a convenient, searchable online database (https://www.peptracker.com/epd).

## Experimental Procedures

Detailed procedures for SILAC labeling, sub-cellular fractionation, LC-MS/MS and data analysis, microscopy, RNAi treatment, and in vivo assays are included in the Supplemental Experimental Procedures.

### *C. elegans* Strains and Maintenance

The N2 (Bristol) strain was used as wild-type. HZ859 was a gift from Hong Zhang and Malene Hansen, GFP-SKL was from Monica Driscoll, and TH184 and TH237 were from Mihail Sarov. All other strains were from the Caenorhabditis Genetics Center (CGC). The strains were grown and maintained at 20°C as previously described (Brenner, 1974) with sufficient food (*E. coli* OP50 unless otherwise indicated) for at least three generations prior to use.

### Data Sharing

The data have been assembled into a searchable, online resource with a user-friendly graphical interface, maintained by the A.I.L. lab, to provide convenient and open access to the community (https://www.peptracker.com/epd/). Our data will also shortly be incorporated into WormBase, a resource that is used extensively by the worm community.

## Author Contributions

V.N. conceived and performed the experiments, with input from T.L., A.G., A.I.L., and C.K. V.N., A.I.L., A.G., and C.K. secured funding. V.N. and T.L. wrote R scripts for data analysis. E.P. and V.N. optimized SILAC protocols for worms. A.B.M. integrated the proteomic data into the EPD (https://www.peptracker.com/epd/). V.N., C.K., A.I.L., T.L., and A.G. wrote the manuscript.

## Accession Numbers

The accession numbers for all of the raw MS data, as well as MaxQuant output files (containing protein identification/matching and quantification data), reported in this paper are ProteomeXchange Consortium (http://proteomecentral.proteomexchange.org): PXD004584 and MassIVE (http://massive.ucsd.edu/ProteoSAFe/): MSV000079263.

## Supplemental Information

Document S1. Supplemental Experimental Procedures and Figures S1–S8

Table S1. Sub-cellular Localization Data, Related to Figure 1

Table S2. Identified Proteins, Related to Figure 1

Table S3. Quantified Proteins, Related to Figures 1 and 2

Table S4. Age-Variant Proteins, Related to Figure 2

Document S2. Article plus Supplemental Information
