# Supplementary material for: Deep Proteome Analysis Identifies Age-Related Processes in C. elegans
Source: Cell Syst. 2016 Aug 24;3(2):144–59. doi: 10.1016/j.cels.2016.06.011 (PMC5003814; doi:10.1016/j.cels.2016.06.011)
Supplement: Document S1. Supplemental Experimental Procedures and Figures S1–S8 [file mmc1.pdf]

Cell Systems, Volume *3*

Supplemental Information

Deep Proteome Analysis Identifies Age-Related Processes in *C. elegans*

Vikram Narayan, Tony Ly, Ehsan Pourkarimi, Alejandro Brenes Murillo, Anton Gartner, Angus I. Lamond, and Cynthia Kenyon

Figure S1

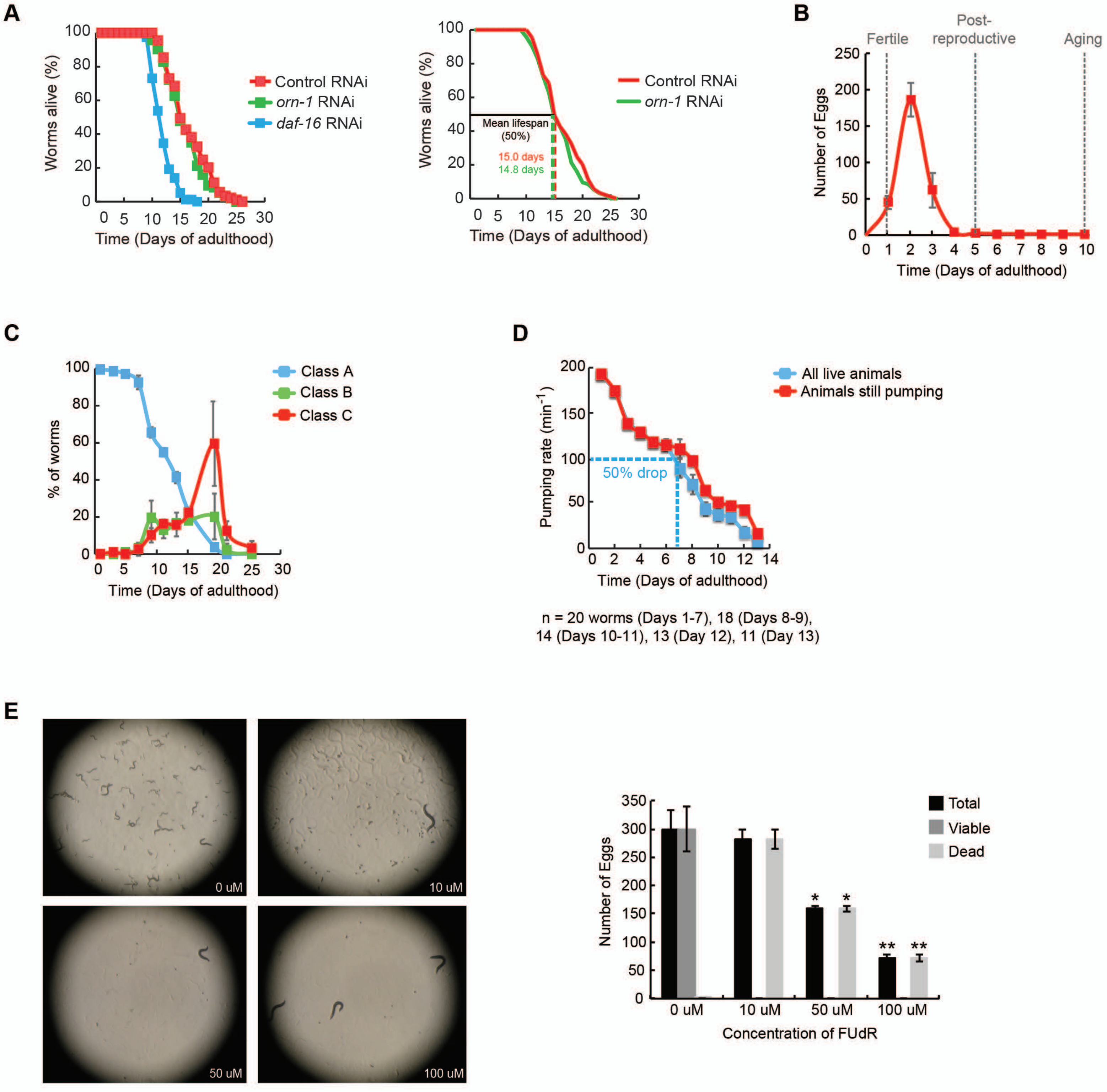

F

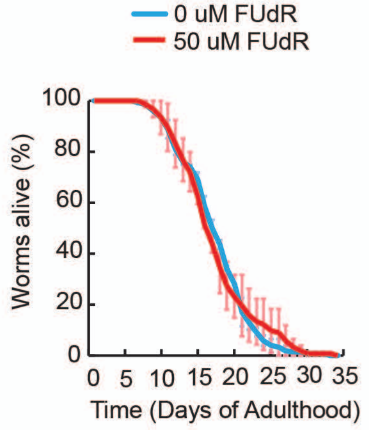

Background	Experiment	Treatment	#Total worms	#Censored	#Scored	Mean lifespan (days of adulthood)
N2	1	0 uM FUdR	100	21	79	16.4
		50 uM FUdR	100	6	94	16.2
N2	2	0 uM FUdR	100	25	75	17.2
		50 uM FUdR	100	24	76	16

Figure S2

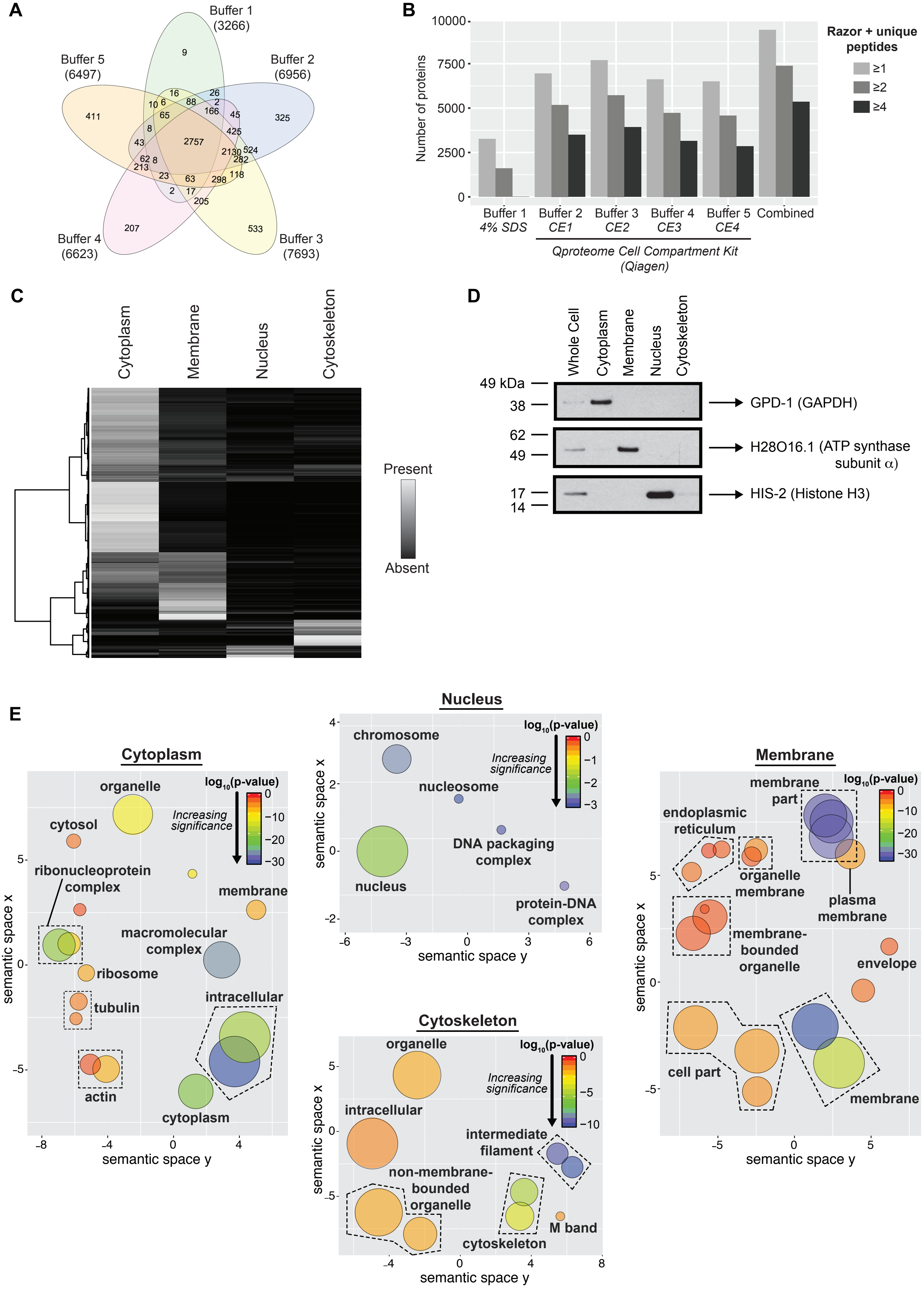
A
Buffer 1
(3266)
Buffer 2
(6956)
Buffer 3
(7693)
Buffer 4
(6623)
Buffer 5
(6497)
9
16
26
10
6
88
2
166
411
65
45
325
8
425
43
2757
2130
524
62
8
282
213
23
63
298
118
2
17
205
207
533
B
Number of proteins
10000
7500
5000
2500
0
Razor + unique peptides
≥1
≥2
≥4
Buffer 1
4% SDS
Buffer 2
CE1
Buffer 3
CE2
Buffer 4
CE3
Buffer 5
CE4
Combined
Qproteome Cell Compartment Kit
(Qiagen)
C
Cytoplasm
Membrane
Nucleus
Cytoskeleton
Present
Absent
D
Whole Cell
Cytoplasm
Membrane
Nucleus
Cytoskeleton
49 kDa
38
62
49
17
14
GPD-1 (GAPDH)
H28O16.1 (ATP synthase subunit α)
HIS-2 (Histone H3)
E
Cytoplasm
organelle
cytosol
ribonucleoprotein complex
membrane
macromolecular complex
ribosome
tubulin
intracellular
actin
cytoplasm
log10(p-value)
Increasing significance
semantic space x
semantic space y
Nucleus
chromosome
nucleosome
DNA packaging complex
nucleus
protein-DNA complex
Cytoskeleton
organelle
intracellular
intermediate filament
non-membrane-bounded organelle
cytoskeleton
M band
Membrane
membrane part
endoplasmic reticulum
organelle membrane
plasma membrane
membrane-bounded organelle
envelope
cell part
membrane

Figure S2

F

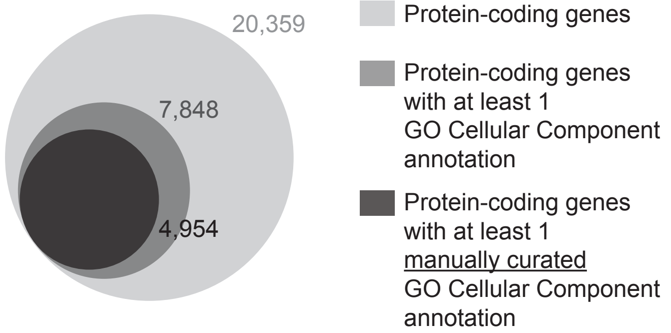

G

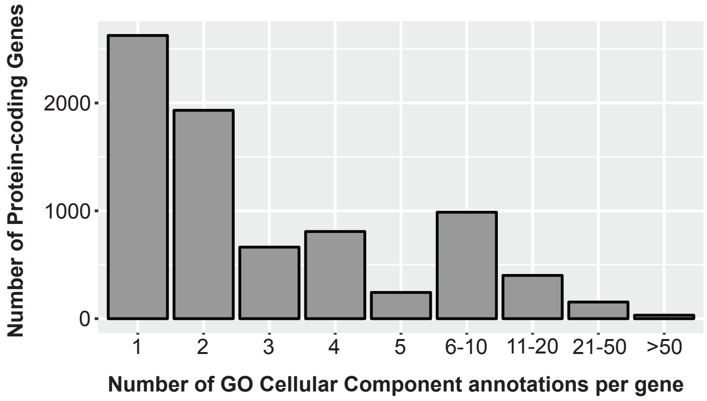

H

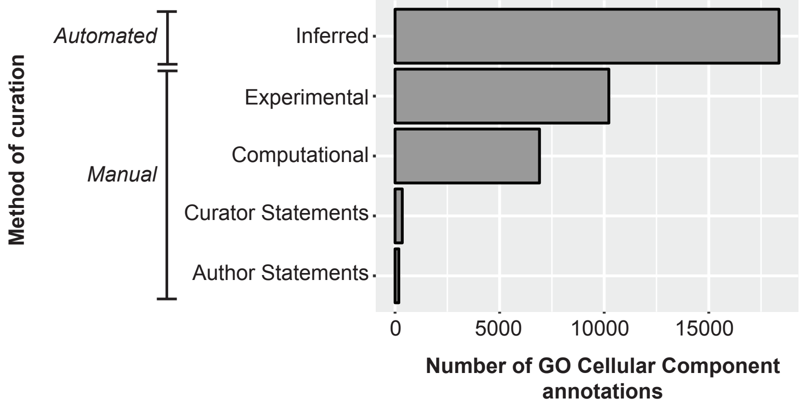

Figure S3

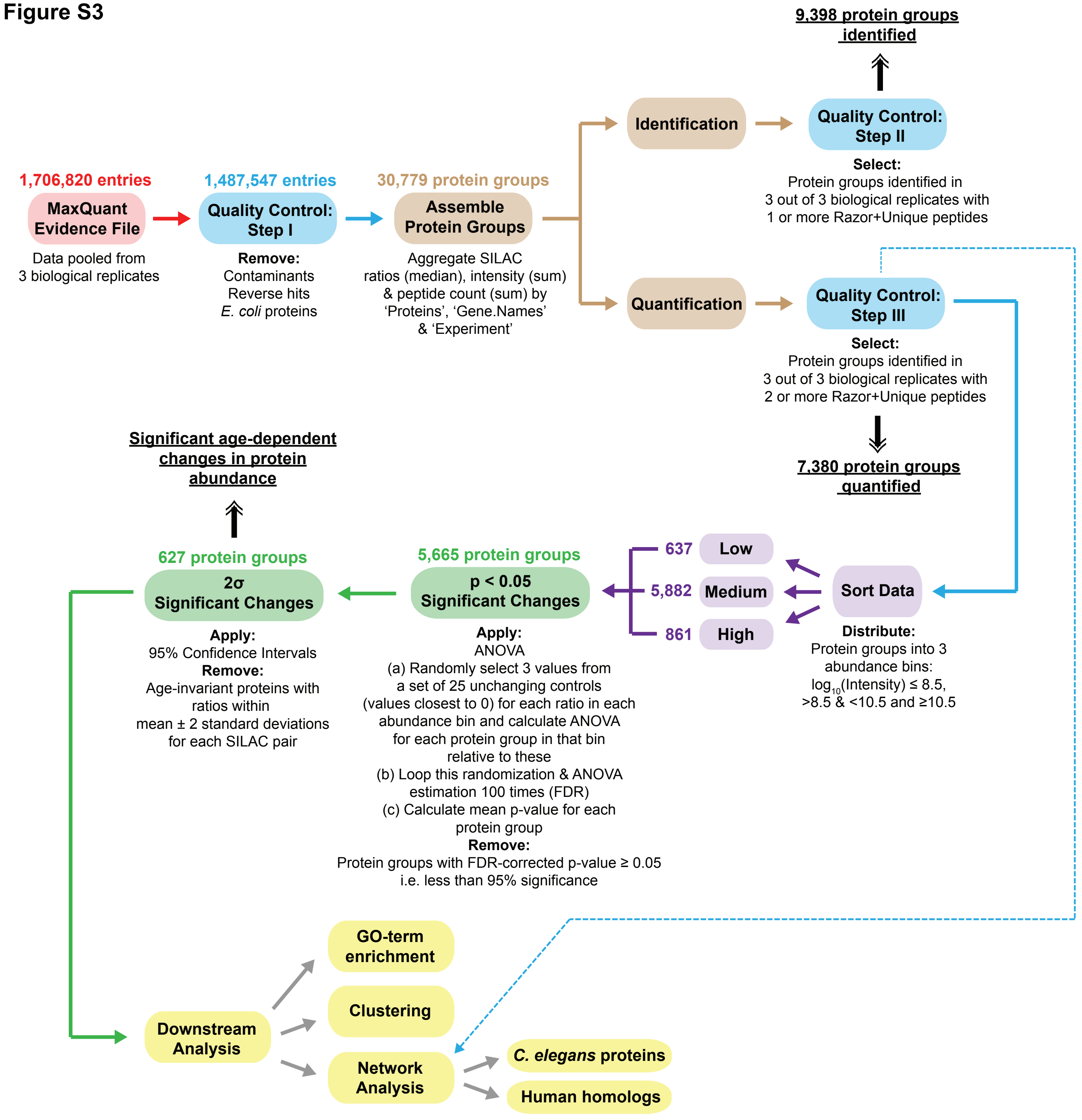
9,398 protein groups identified
1,706,820 entries
MaxQuant Evidence File
Data pooled from 3 biological replicates
1,487,547 entries
Quality Control: Step I
Remove:
Contaminants
Reverse hits
E. coli proteins
30,779 protein groups
Assemble Protein Groups
Aggregate SILAC ratios (median), intensity (sum) & peptide count (sum) by 'Proteins', 'Gene.Names' & 'Experiment'
Identification
Quality Control: Step II
Select:
Protein groups identified in 3 out of 3 biological replicates with 1 or more Razor+Unique peptides
Quantification
Quality Control: Step III
Select:
Protein groups identified in 3 out of 3 biological replicates with 2 or more Razor+Unique peptides
7,380 protein groups quantified
Significant age-dependent changes in protein abundance
627 protein groups
2σ Significant Changes
Apply:
95% Confidence Intervals
Remove:
Age-invariant proteins with ratios within mean ± 2 standard deviations for each SILAC pair
5,665 protein groups
p < 0.05 Significant Changes
Apply:
ANOVA
(a) Randomly select 3 values from a set of 25 unchanging controls (values closest to 0) for each ratio in each abundance bin and calculate ANOVA for each protein group in that bin relative to these
(b) Loop this randomization & ANOVA estimation 100 times (FDR)
(c) Calculate mean p-value for each protein group
Remove:
Protein groups with FDR-corrected p-value ≥ 0.05 i.e. less than 95% significance
637
Low
5,882
Medium
861
High
Sort Data
Distribute:
Protein groups into 3 abundance bins:
log10(Intensity) ≤ 8.5, >8.5 & <10.5 and ≥10.5
Downstream Analysis
GO-term enrichment
Clustering
Network Analysis
C. elegans proteins
Human homologs

Figure S4

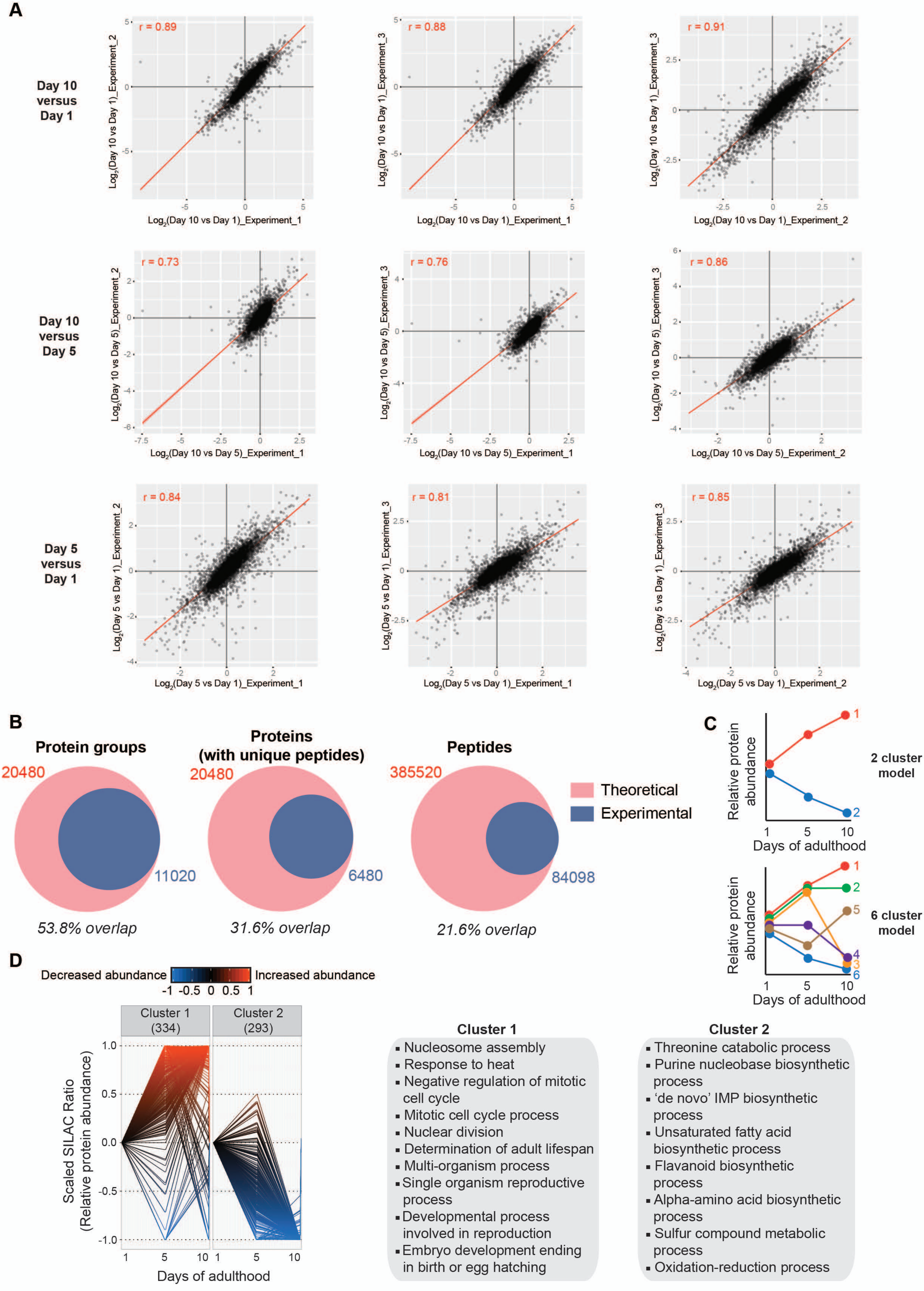

A
Day 10 versus Day 1
r = 0.89
r = 0.88
r = 0.91
Log2(Day 10 vs Day 1)_Experiment_1
Log2(Day 10 vs Day 1)_Experiment_2
Log2(Day 10 vs Day 1)_Experiment_3
Day 10 versus Day 5
r = 0.73
r = 0.76
r = 0.86
Log2(Day 10 vs Day 5)_Experiment_1
Log2(Day 10 vs Day 5)_Experiment_2
Log2(Day 10 vs Day 5)_Experiment_3
Day 5 versus Day 1
r = 0.84
r = 0.81
r = 0.85
Log2(Day 5 vs Day 1)_Experiment_1
Log2(Day 5 vs Day 1)_Experiment_2
Log2(Day 5 vs Day 1)_Experiment_3
B
Protein groups
20480
11020
53.8% overlap
Proteins (with unique peptides)
20480
6480
31.6% overlap
Peptides
385520
84098
21.6% overlap
Theoretical
Experimental
C
Relative protein abundance
2 cluster model
6 cluster model
Days of adulthood
D
Decreased abundance
Increased abundance
-1 -0.5 0 0.5 1
Cluster 1 (334)
Cluster 2 (293)
Scaled SILAC Ratio (Relative protein abundance)
Days of adulthood
Cluster 1
Nucleosome assembly
Response to heat
Negative regulation of mitotic cell cycle
Mitotic cell cycle process
Nuclear division
Determination of adult lifespan
Multi-organism process
Single organism reproductive process
Developmental process involved in reproduction
Embryo development ending in birth or egg hatching
Cluster 2
Threonine catabolic process
Purine nucleobase biosynthetic process
'de novo' IMP biosynthetic process
Unsaturated fatty acid biosynthetic process
Flavanoid biosynthetic process
Alpha-amino acid biosynthetic process
Sulfur compound metabolic process
Oxidation-reduction process

Figure S5

A

Day 10 vs Day 5: Upregulated KEGG pathways

NAME	NES	NOM p-val	FDR q-val
KEGG_VIRAL_MYOCARDITIS	2.1533	0.0000	0.0450
KEGG_DNA_REPLICATION	1.5842	0.0000	0.2558
KEGG_COLORECTAL_CANCER	1.5057	0.0000	0.3044
KEGG_LEUKOCYTE_TRANSENDOTHELIAL_MIGRATION	1.4806	0.0000	0.2396
KEGG_NUCLEOTIDE_EXCISION_REPAIR	1.3546	0.0000	0.5439
KEGG_RNA_DEGRADATION	1.3420	0.0000	0.4912
KEGG_CELL_CYCLE	1.3398	0.0000	0.4274
KEGG_PROGESTERONE_MEDIATED_OOCYTE_MATURATION	1.1921	0.2894	1.0000
KEGG_AMINOACYL_TRNA_BIOSYNTHESIS	1.1787	0.0000	0.9867
KEGG_PATHWAYS_IN_CANCER	1.1783	0.2702	0.8925
KEGG_WNT_SIGNALING_PATHWAY	1.1773	0.2702	0.8155
KEGG_AXON_GUIDANCE	1.1168	0.3110	1.0000
KEGG_SPLICEOSOME	1.0939	0.2903	1.0000
KEGG_BASAL_TRANSCRIPTION_FACTORS	1.0655	0.6190	1.0000
KEGG_ADHERENS_JUNCTION	1.0628	0.0000	0.9950
KEGG_PROSTATE_CANCER	1.0476	0.3488	0.9911
KEGG_VEGF_SIGNALING_PATHWAY	1.0370	0.3295	0.9383
KEGG_TIGHT_JUNCTION	1.0344	0.2702	0.9081
KEGG_PROTEASOME	1.0266	0.2903	0.8652
KEGG_FC_GAMMA_R_MEDIATED_PHAGOCYTOSIS	1.0200	0.3488	0.8333
KEGG_MTOR_SIGNALING_PATHWAY	1.0087	0.3359	0.8329
KEGG_ENDOCYTOSIS	0.9947	0.2903	0.8111
KEGG_UBIQUITIN_MEDIATED_PROTEOLYSIS	0.9890	0.3488	0.7989
KEGG_RENAL_CELL_CARCINOMA	0.9864	0.6391	0.7675
KEGG_MAPK_SIGNALING_PATHWAY	0.9787	0.2602	0.7532

Day 10 vs Day 5: Downregulated KEGG pathways

NAME	NES	NOM p-val	FDR q-val
KEGG_TRYPTOPHAN_METABOLISM	-1.6700	0.0000	0.4179
KEGG_BUTANOATE_METABOLISM	-1.5473	0.0000	0.3489
KEGG_RIBOSOME	-1.5399	0.2780	0.2476
KEGG_PPAR_SIGNALING_PATHWAY	-1.5263	0.0000	0.2384
KEGG_STARCH_AND_SUCROSE_METABOLISM	-1.4929	0.0000	0.2132
KEGG_PEROXISOME	-1.4351	0.0000	0.2485
KEGG_PROPANOATE_METABOLISM	-1.4291	0.0000	0.2617
KEGG_MELANOGENESIS	-1.3842	0.0000	0.2784
KEGG_ARACHIDONIC_ACID_METABOLISM	-1.3644	0.0000	0.2886
KEGG_STEROID_HORMONE_BIOSYNTHESIS	-1.3551	0.0000	0.2974
KEGG_GLYCOLYSIS_GLUCONEOGENESIS	-1.3481	0.0000	0.2744
KEGG_LYSINE_DEGRADATION	-1.3304	0.0000	0.3178
KEGG_ASCORBATE_AND_ALDARATE_METABOLISM	-1.3163	0.0000	0.3209
KEGG_FATTY_ACID_METABOLISM	-1.2962	0.0000	0.3700
KEGG_PYRUVATE_METABOLISM	-1.2432	0.3165	0.4800
KEGG_RETINOL_METABOLISM	-1.2389	0.0000	0.4736
KEGG_DRUG_METABOLISM_CYTOCHROME_P450	-1.2354	0.0000	0.4583
KEGG_METABOLISM_OF_XENOBIOTICS_BY_CYTOCHROME_P450	-1.2303	0.0000	0.4445
KEGG_EPITHELIAL_CELL_SIGNALING_IN_H_PYLORI_INFECTION	-1.2273	0.3419	0.4323
KEGG_CITRATE_CYCLE_TCA_CYCLE	-1.2267	0.2960	0.4129
KEGG_DRUG_METABOLISM_OTHER_ENZYMES	-1.2266	0.0000	0.3954
KEGG_ARGININE_AND_PROLINE_METABOLISM	-1.2201	0.2702	0.3867
KEGG_GLUTATHIONE_METABOLISM	-1.2200	0.3488	0.3718
KEGG_PENTOSE_AND_GLUCURONATE_INTERCONVERSIONS	-1.2043	0.0000	0.3893
KEGG_ALANINE_ASPARTATE_AND_GLUTAMATE_METABOLISM	-1.1700	0.0000	0.4330

B

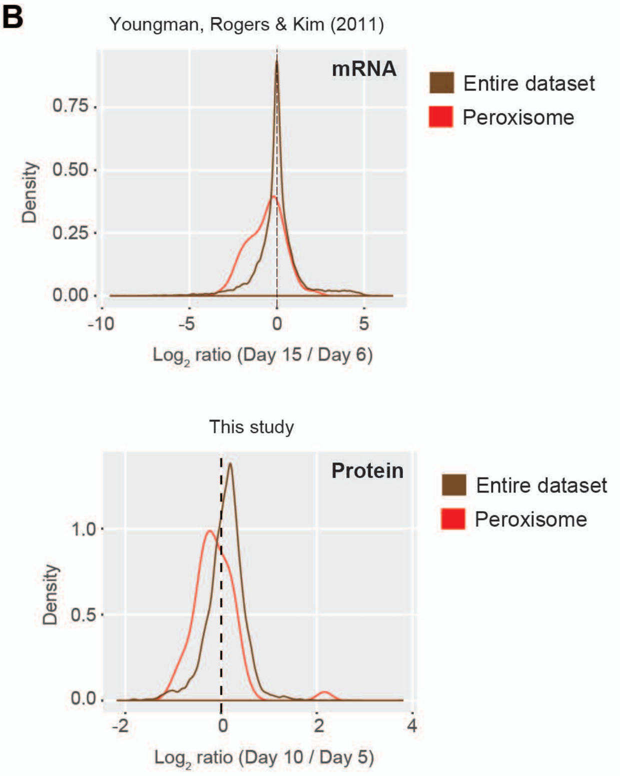

C

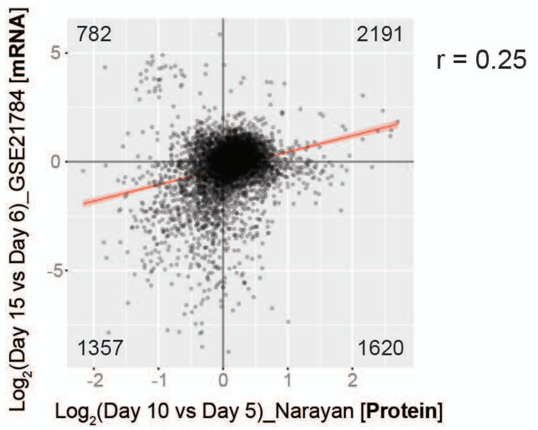

Figure S6

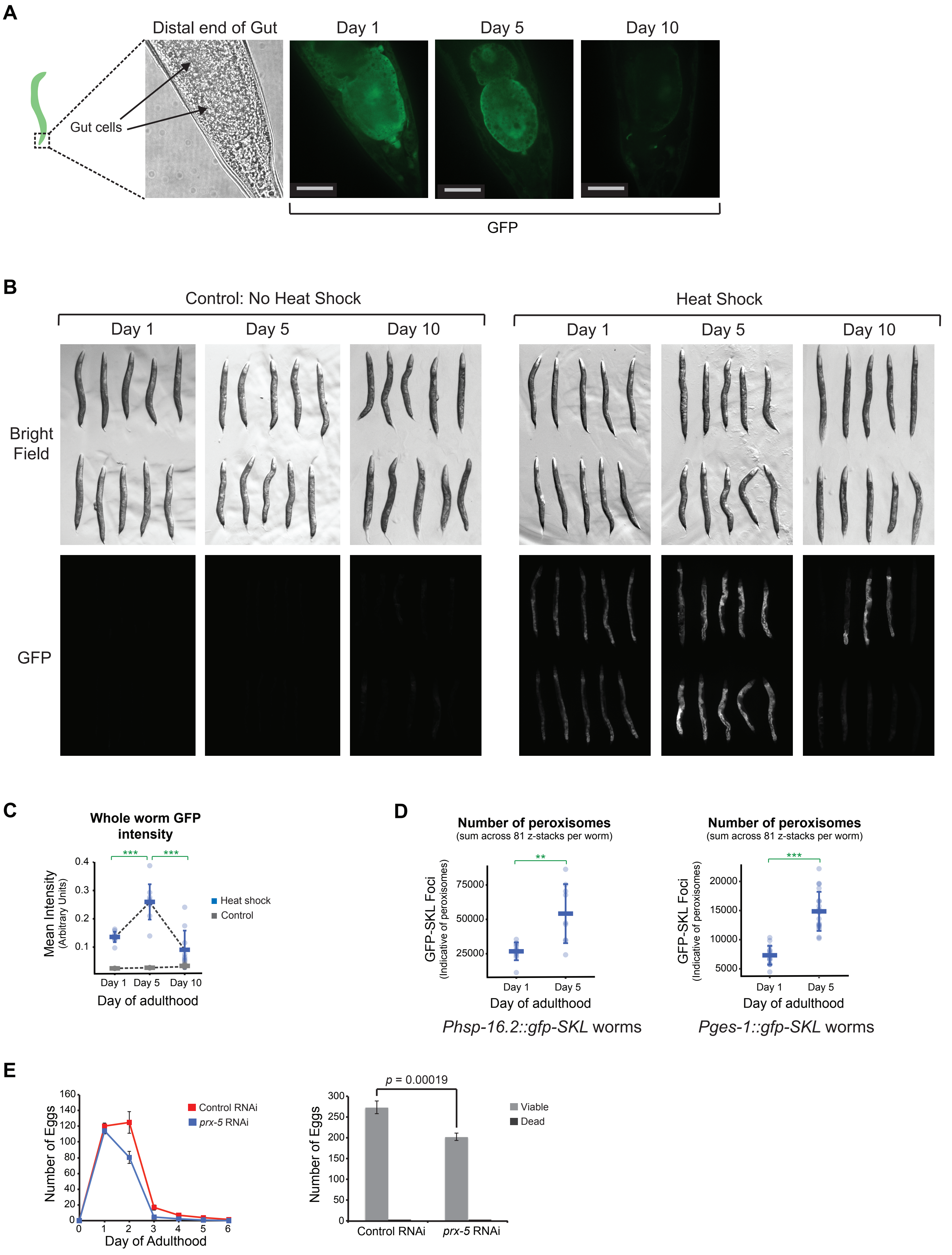
A
Distal end of Gut
Day 1
Day 5
Day 10
Gut cells
GFP
B
Control: No Heat Shock
Heat Shock
Day 1
Day 5
Day 10
Bright Field
GFP
C
Whole worm GFP intensity
Mean Intensity (Arbitrary Units)
Heat shock
Control
Day of adulthood
D
Number of peroxisomes
(sum across 81 z-stacks per worm)
GFP-SKL Foci (Indicative of peroxisomes)
Day of adulthood
Phsp-16.2::gfp-SKL worms
Pges-1::gfp-SKL worms
E
Number of Eggs
Control RNAi
prx-5 RNAi
Day of Adulthood
p = 0.00019
Viable
Dead

Figure S7

Microscopy data (n = 20 individual worms except for SQST-1 Day 10 males, where n = 5 individual worms)
Mass spectrometry data (n = 3 biological replicates)
Graphs show sample mean ± standard deviation

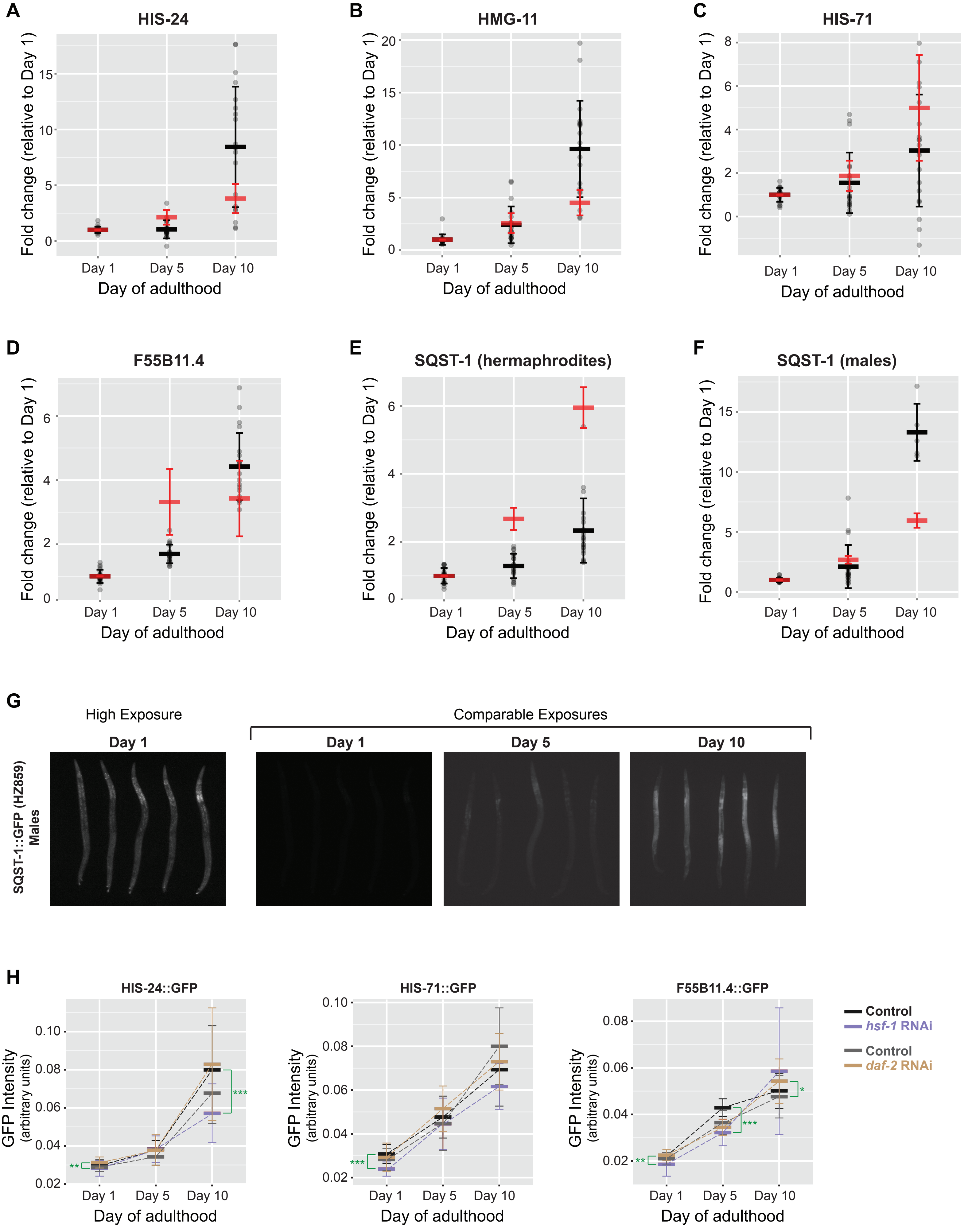

Figure S8

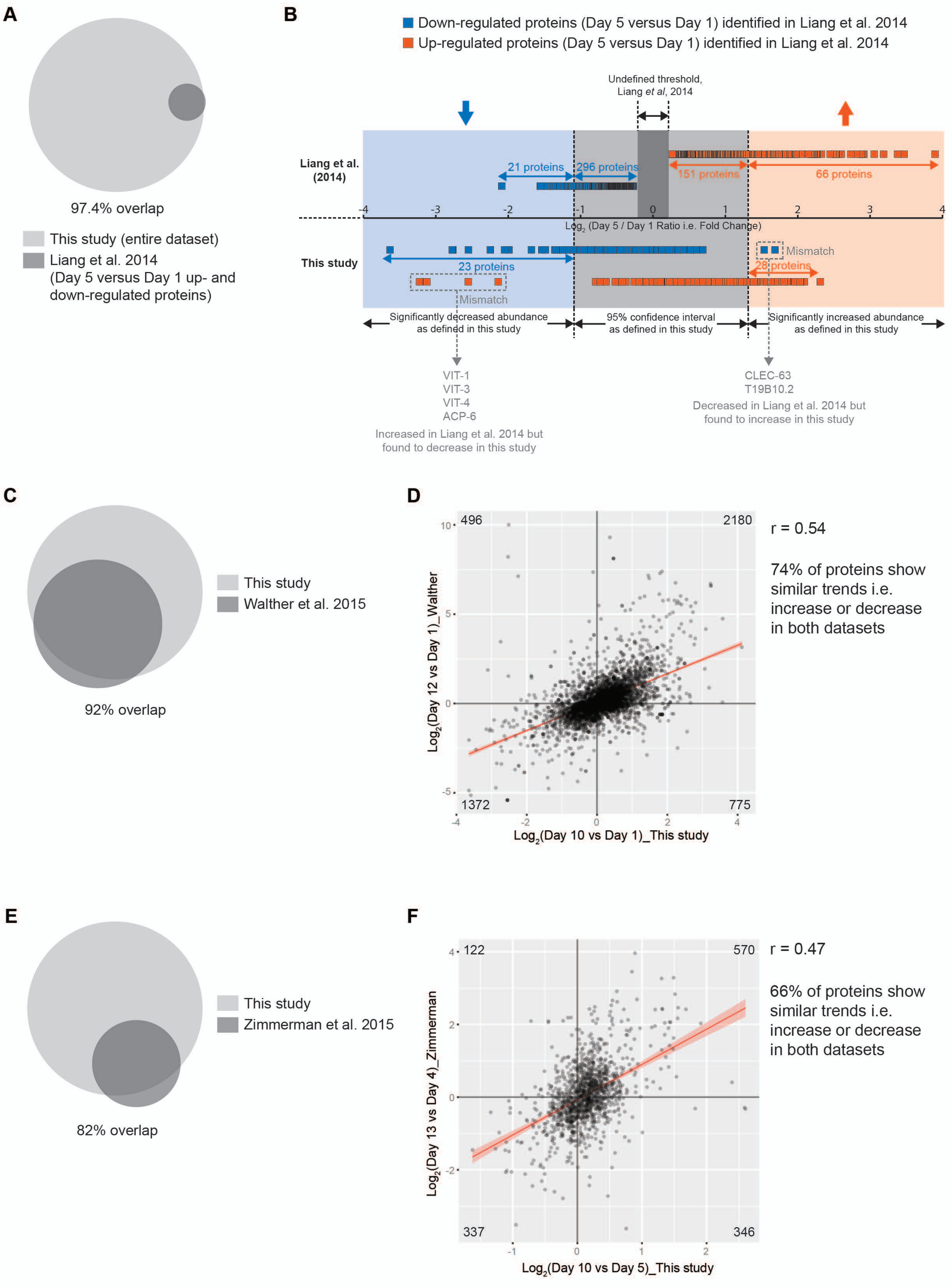
A
97.4% overlap
This study (entire dataset)
Liang et al. 2014
(Day 5 versus Day 1 up- and down-regulated proteins)
B
Down-regulated proteins (Day 5 versus Day 1) identified in Liang et al. 2014
Up-regulated proteins (Day 5 versus Day 1) identified in Liang et al. 2014
Undefined threshold, Liang et al, 2014
Liang et al. (2014)
21 proteins
296 proteins
151 proteins
66 proteins
-4 -3 -2 -1 0 1 2 3 4
Log2 (Day 5 / Day 1 Ratio i.e. Fold Change)
This study
23 proteins
Mismatch
28 proteins
Mismatch
Significantly decreased abundance as defined in this study
95% confidence interval as defined in this study
Significantly increased abundance as defined in this study
VIT-1
VIT-3
VIT-4
ACP-6
Increased in Liang et al. 2014 but found to decrease in this study
CLEC-63
T19B10.2
Decreased in Liang et al. 2014 but found to increase in this study
C
This study
Walther et al. 2015
92% overlap
D
496
2180
1372
775
Log2(Day 12 vs Day 1)_Walther
Log2(Day 10 vs Day 1)_This study
r = 0.54
74% of proteins show similar trends i.e. increase or decrease in both datasets
E
This study
Zimmerman et al. 2015
82% overlap
F
122
570
337
346
Log2(Day 13 vs Day 4)_Zimmerman
Log2(Day 10 vs Day 5)_This study
r = 0.47
66% of proteins show similar trends i.e. increase or decrease in both datasets

SUPPLEMENTAL FIGURE LEGENDS

Figure S1. FUdR treatment does not affect adult *C. elegans* lifespan [Related to Figure 1]
(**A**) Wild-type N2 (Bristol) animals were grown on HT115 *E. coli* at 20°C from hatching and transferred to the indicated RNAi bacteria at mid-late L4. Animals were scored as alive or dead on each day of adulthood until the entire population was dead; missing, bagged or exploded animals were censored. n = 89 (L4440 empty vector RNAi control), n = 94 (*orn-1* RNAi) and n = 78 (*daf-16* RNAi); n does not include censored animals; n = 100 per condition prior to censoring. The graph on the right was plotted without depicting any data points, for clarity, and shows the mean lifespan for wild-type and *orn-1(RNAi)* animals. (**B**) Graph of number of eggs laid in wild-type N2 (Bristol) worms at 20°C between days 1 to 10 of adulthood. Day 1 was considered to be 3 days after hatching (24 h after L4). 20 adult animals were scored; mean eggs laid ± standard error of mean is indicated on the graph. The time points used in this study (days 1, 5 and 10 of adulthood) are highlighted. (**C**) The lifespan of wild-type N2 (Bristol) worms (n = 100 per experiment) at 20°C was measured; missing, bagged or exploded animals were censored. On each day of adulthood, the number of Class A, B and C animals, designated based on motility as described in Herndon et al. 2002, was scored. Shown is the mean ± standard deviation from two independent experiments. (**D**) Graph of the rate of pharyngeal pumping per minute plotted against the age of the animals. Pharyngeal pumping decreases markedly early in adulthood and is virtually undetectable by day 13. Animals (n = 20; see figure for details) were scored for 3 x 30 s intervals every 24 h and the mean pumping rate per minute for each animal calculated. The graph shows the average of the mean pumping rate per minute per worm ± standard error of mean. Pumping could not be detected in some animals after day 7. (**E**) N2 (Bristol) animals were treated with the indicated concentrations of FUdR beginning from the mid-late L4 larval stage. Shown are images taken on day 2 of adulthood (*left*) and total number of eggs laid (*right*; n = 20; graph shows mean ± standard deviation) up to day 10 of adulthood. All FUdR doses tested completely prevented eggs from hatching, as is evident from the images. The total number of eggs laid was significantly lower at doses higher than 50 μM (* = p-value from t-test compared to 0 μM control < 0.05; ** = p-value < 0.01). (**F**) The lifespan of N2 (Bristol) animals on 0 or 50 μM FUdR (treatment was initiated at mid-late L4) at 20°C was scored. Missing, bagged or exploded animals were censored. Shown is the mean ± standard error of mean from 2 independent experiments.

Figure S2. Differential buffer extraction reveals putative sub-cellular localization profiles for ~6,000 *C. elegans* proteins [Related to Figure 1 and Table S1]
(**A**) Venn diagram (not area-proportionate; generated using Interactivenn) showing proteins identified in all 3 biological replicates using the 5-buffer differential extraction method employed in this study that were unique to each buffer, as well as the overlap between buffers. Buffer 1 is a 4% SDS-based buffer (see Supplemental Experimental Procedures) and buffers 2-5 are from the Qiagen QProteome Cell Compartment Kit (CE1, CE2, CE3 and CE4 respectively). CE1 = cytoplasmic fraction, CE2 = membrane fraction, CE3 = nuclear fraction, CE4 = cytoskeletal fraction and insoluble materials. (**B**) Bar plot showing the number of proteins identified using each of the 5 buffers in (A) with 1 or more, 2 or more and 4 or more razor/unique peptides. Also included is the aggregate data wherein peptides identified using each buffer were pooled and compiled into a single dataset ("Combined"). (**C**) Heat map showing the distribution of 6,283 proteins across the indicated sub-cellular compartments, isolated using the QProteome Cell Compartment Kit (Qiagen), in day 1 adult *C. elegans* (also see Table S1, sheet 2). (**D**) 2.5 μg of each sub-cellular fraction or 4% SDS-extracted whole-cell lysate were run out on a 4-12% NuPAGE gel (Thermo Fisher) and analyzed by immunoblotting with the following antibodies: anti-GAPDH (Sigma; 1:5000), anti-Histone H3 (Abcam; 1:4000) and anti-ATP synthase subunit α (Mitosciences; 1:2000). (**E**) GO-term enrichment (Cellular Component) of the proteins present in each sub-cellular compartment was performed using PANTHER and plotted using REVIGO, in order to validate the efficacy of the extraction methodology in nematodes. The size of the bubbles is indicative of the number of proteins annotated with that GO-term (range: 9-309 for cytoplasm; 7-168 for membrane; 5-14 for nucleus; 4-60 for cytoskeleton); bubbles are color-coded according to significance (Bonferroni-corrected p-value). To reduce GO-term redundancy, the 'PANTHER GO-Slim Cellular Component' annotation data set was used unless the output was predominantly "unclassified", in which case 'GO Cellular Component complete' was used. Only those proteins whose levels in a single fraction were found to be 75% or more of their total abundance across all fractions were included in the analysis [i.e. (intensity in a single fraction / sum of intensities across all 4 fractions) x 100 >= 75]. This subset included 2,297 cytoplasmic proteins, 354 membrane proteins, 78 nuclear proteins and 321 cytoskeletal proteins (see Table S1, sheets 3-6). (**F**) Area-proportionate Venn diagram (generated using BioVenn) showing that only ~39% of the theoretical *C. elegans* protein-coding genes (20,359 according to

WormBase Release WS250, 31 July 2015) have at least one Cellular Component GO-term annotation (7,848; GO_CC), and of these, only 4,954 have a manually curated GO_CC annotation. (**G**) Histogram showing the number of GO_CC annotations assigned to each of the 7,848 *C. elegans* protein-coding genes with a GO_CC annotation. (**H**) Graph showing the breakdown of GO_CC assignments to the 7,848 *C. elegans* protein-coding genes with at least one GO_CC annotation. As stated in (F), 4,954 of these genes (63%) have a manually curated GO_CC annotation. However, only a subset of the manually curated GO_CC annotations (~10,000 annotations, corresponding to 2,121 protein-coding genes) is based on experimental evidence. Thus, only about 10% (2,121 out of 20,359) of all *C. elegans* protein-coding genes have a GO_CC assignment based on experimental data. (**F-H**) Raw data was obtained from WormBase, and mined/queried using WormMine.

Figure S3. Data analysis workflow [Related to Figures 1 and 2, and Tables S2-S4]
Schematic representation of the data analysis pipeline used in this study.

Figure S4. Assessment of proteomics data quality [Related to Figures 1 and 2]
(**A**) Scatter plots depicting $\log_2$-transformed SILAC ratios (for each pair-wise comparison of the 3 time-points used in this study) between the 3 biological replicates. Reproducibility of protein quantification across the replicates was high, as indicated by the Pearson's r values shown on the upper left corner of each scatter plot. (**B**) *Left*: Area-proportionate Venn diagram (generated using BioVenn) showing the overlap between proteins identified in this study (adults only; "Experimental") versus the theoretical *C. elegans* proteome comprising all developmental stages, as defined by WormBase (Release WS243, 28 March 2014 containing 20,480 protein-coding genes; "Theoretical"). *Center*: Area-proportionate Venn diagram of the theoretical *C. elegans* proteome (as above) and the number of proteins reproducibly identified with sequence-unique peptides in this study ("Experimental"). The "Experimental" dataset here does not, therefore, include peptides that were assigned to protein groups rather than individual proteins. *Right*: A theoretical tryptic digest of the *C. elegans* proteome was generated and a list of putative MS-flyable peptides compiled (conditions imposed: peptides must have at least one Lys or Arg, and must be 6-35 amino acids in length; "Theoretical"). This list was compared to the sequence-unique peptides identified in this study ("Experimental") and is represented as an area-proportionate Venn diagram. (**C**) Cartoons showing representative profiles of the 627 age-variant proteins (see main Figure 2) grouped by hierarchical clustering into either 2 clusters (*top*) or 6 clusters (*bottom*). (**D**) Hierarchical clustering of the 627 proteins using Ward's minimum variance method into 2 groups based on trend profiles. To minimize redundancy in clustering due to large differences in the magnitude of the abundance changes, the SILAC ratios were scaled to range between -1 (maximum decrease) and +1 (maximum increase) with ratios near 0 indicating no change in abundance during the 3 time points considered in this study. Results from GO-term enrichment analysis (multiple hypothesis [Bonferroni] corrected $p < 0.05$) of each cluster using PANTHER are also depicted.

Figure S5. Gene Set Enrichment Analysis of proteomics data reveals pathways altered during nematode aging [Related to Figures 3 and 4]
(**A**) Protein quantification data for day-10 versus day-5 of adulthood, as measured in this study, were analyzed using gene set enrichment analysis to identify KEGG pathways that change during aging. Shown is the GSEA output using the algorithm from the Broad Institute. (**B**) Density plots depicting the abundance of a gene set comprising KEGG 'peroxisome' genes relative to the entire dataset. The curve for peroxisome genes includes those only those genes that were detected both in a study by Youngman et al. 2011 measuring mRNA levels and in this study, measuring protein levels. *Top*: density plot for peroxisome mRNA transcripts relative to the entire Youngman et al. 2011 mRNA dataset; *Bottom*: density plot for peroxisome protein levels relative to the entire protein dataset measured in this study. (**C**) Scatter plot showing mRNA levels measured in a study by Youngman et al. 2011 plotted against the levels of their corresponding protein products as measured in this study. The mRNA and protein data show poor correlation ($r = 0.25$ [Pearson]). A linear model, calculated using the function lm in R, is depicted by the red line. 95% confidence intervals for the linear model are shaded light red.

Figure S6. Control experiments for GFP-SKL *C. elegans* strains [Related to Figure 5]
(**A**) In order to ensure that the GFP-SKL foci observed in young *Phsp-16.2::gfp-SKL* worms after induction of GFP-SKL expression by heat shock were not merely artifacts or stress granules, *Phsp-16.2::gfp* animals, lacking the peroxisome-targeting -SKL signal were used (strain CL2070). No foci were detected in these animals after heat shock. Similar to data from GFP-SKL animals (see below), *hsp-16.2* driven GFP expression was markedly decreased by day 10 of adulthood. (**B & C**) Whole-body *hsp-16.2*-driven GFP-SKL intensity was measured and found to increase significantly between days 1

and 5 of adulthood, and then drop markedly by day 10 (p-value from unpaired t-test = 1.02 x 10^{-4} for day 1 versus day 5; 1.70 x 10^{-5} for day 5 versus 10; and 0.067 for day 1 versus day 10. n = 10 in all cases). Shown are bright field and GFP images taken with (*right*) and without (*left*) heat shock (**B**) as well as a graph showing quantification of GFP intensity from the images (**C**). (**D**) The number of foci resolved ("peroxisomes") at day 1 and day 5 (based on data presented in main Figures 5C and 5D) was summed across the entire z-stack (81 sections per worm) for GFP-SKL driven by the *hsp-16.2* promoter (*left*) and the *ges-1* promoter (*right*). The number of dots resolved was found to increase significantly by day 5 (p-value from unpaired t-test = 0.0048 (*left*) and 3.07 x 10^{-9} (*right*)). (**E**) N2 (Bristol) worms were treated with control or *prx-5* RNAi from late L4/young adult stage, and the number of eggs laid on each day of adulthood measured (*left*). Also shown is the total number of eggs laid during days 1 to 10 of adulthood (*right*). Graphs show mean ± standard deviation (n = 20 for control RNAi animals and n = 26 for *prx-5(RNAi)* animals). The total number of eggs laid in *prx-5* RNAi animals was found to be significantly less than control animals using t-test (p-value = 0.00019).

Figure S7. Comparison of mass spectrometric predictions with *in vivo* measurements of age-dependent protein changes by microscopy [Related to Figure 6]
(**A-F**) Graphs showing mass spectrometry-determined changes in protein abundance during aging (*red*) compared to *in vivo* fold-change measurements calculated by imaging GFP::protein fusion worm strains (*black*). For microscopy, 20 individual worms were analyzed (see (**G**) and main Figure 6A for snapshots); quantification was performed using Cell Profiler (Broad Institute). Shown is the mean ± standard deviation. Note that the individual variation in protein expression for all GFP::protein fusion strains increased markedly by day 10. SQST-1::GFP worms (strain HZ859) were in a *him-5(e1490)* background; thus, both hermaphrodites (**E** and main Figure 6A) and males (**F, G**) were examined. (**H**) HIS-24::GFP (*left*), HIS-71::GFP (*center*) and F55B11.4::GFP-expressing (*right*) worms were grown on the indicated RNAi (initiated at L1 stage) and GFP-fluorescence was quantified on days 1, 5 and 10 of adulthood. Graphs show mean ± standard deviation (n = 20 worms). Significance was calculated using an unpaired t-test (p-value < 0.05 = *, < 0.01 = **, < 0.001 = ***). The *daf-2* and *hsf-1* RNAi are in different vector backbones (see Supplemental Experimental Procedures); thus, the corresponding empty vector control for each was used.

Figure S8. Comparison between this study and recent proteomics studies (Liang et al. 2014; Walther et al. 2015; Zimmerman et al. 2015) [Related to Figure 1 and Discussion]
(**A**) Area-proportionate Venn diagram showing that 97.4% of the proteins for which quantification data from the Liang et al. 2014 study was publicly available, were also detected in our study. (**B**) Significant fold-changes as determined in the Liang et al. 2014 study based on undefined thresholds, were projected onto our thresholds for significance (95% confidence interval calculated from the mean ± 1.96 standard deviations based on quantification of 7,380 proteins from 3 biological replicates). The majority of the changes deemed significant in the Liang et al. 2014 study were not scored as significant in our study based on our stricter thresholds. The Liang et al. 2014 data (*upper panel*) is compared to our quantification data (*lower panel*) for the same proteins. Also indicated are the mismatches (6 proteins) between the two datasets. (**C**) Area-proportionate Venn diagram showing that 92% of the proteins detected in the Walther et al. 2015 study were also detected in our study. (**D**) Scatter plot for proteins detected in both the Walther et al. 2015 study and this study, showing reproducibility of protein quantification between the studies. A modest correlation was observed (r = 0.54 [Pearson]); this is likely adversely affected by the fact that different quantification strategies were used in the two studies (spike-in SILAC versus triple-SILAC). Additionally, the measured time-points were not identical (day 21 / day 1 data from the Walter et al. 2015 study were compared to day 10 / day 1 data from our study). A linear model, calculated using the function lm in R, is depicted by the red line. 95% confidence intervals for the linear model are shaded light red. Although the magnitude of the fold-change for each protein in the datasets was only moderately similar (reflected in the modest r = 0.54), 74% of the proteins showed similar trends (i.e. increase or decrease) in both datasets. (**E**) Area-proportionate Venn diagram showing that 82% of the proteins detected in the Zimmerman et al. 2015 study were also detected in our study. (**F**) Scatter plot for proteins detected in both the Zimmerman et al. 2015 study and this study, showing reproducibility of protein quantification between the studies. A modest correlation was observed (r = 0.47 [Pearson]); this is likely adversely affected by the fact that different quantification strategies were used in the two studies (dimethyl labeling versus SILAC). Additionally, the measured time-points were not identical. A linear model, calculated using the function lm in R, is depicted by the red line. 95% confidence intervals for the linear model are shaded light red.

SUPPLEMENTAL EXPERIMENTAL PROCEDURES

Reagents

EZQ protein assay, Triscarboxyethylphosphine (TCEP), CBQCA assay, Pepmap C18 (2 cm x 75 µm) trap columns and EasySpray C18 columns (2 µm particles, 50 cm x 75 µm) were from Thermo Fisher Scientific (Waltham, MA). MS-Grade Trypsin was from Promega. Sep-Pak tC18 µ-elution 96-well plates were purchased from Waters. Purified arginine and lysine for SILAC experiments were from Cambridge Isotopes Laboratories (Tewksbury, MA) via CK Gas and were as follows: Light (Arg0, ULM-8347; Lys0, ULM-8766); Medium (Arg6, CLM-2265; Lys4, DLM-2640); and Heavy (Arg10, CNLM-539; Lys8, CNLM-291). All other materials were obtained from Sigma, unless otherwise indicated.

RNAi experiments

RNA interference experiments were performed using the RNAi via feeding strategy [Ahringer library; (Kamath et al. 2001)]. The exception was the *daf-2* RNAi construct used (pAD48-*daf-2*-RNAi; Addgene plasmid #34834 and corresponding empty vector control pAD12; Addgene plasmid #34832), which was made in-house, and has been characterized previously (Dillin et al. 2002). RNAi of *orn-1* for SILAC experiments was performed as described previously (Larance et al. 2015; Larance et al. 2011). *prx-5* RNAi was initiated during 'day 0 of adulthood', just prior to the appearance of eggs in the hermaphrodite animal, as described in Zhou et al. 2012. All other RNAi experiments were initiated at the L1 stage and the resulting adults were imaged as described in the main Experimental Procedures.

Lifespan assays

Lifespan assays were performed at 20°C as described previously, except that days of adulthood (day 1 = 24 h post L4) were reported in our study (Hsin & Kenyon 1999). Additionally, for *orn-1* and *daf-16* RNAi experiments, RNAi treatment was initiated at L4 and FUdR was not used; the animals were instead transferred to fresh RNAi plates every other day. For FUdR lifespan assays (dose response), synchronized wild-type L4 animals were transferred onto to NGM/OP50 plates containing the required concentration of FUdR and assessed for survival on each day of adulthood. Animals that exploded, bagged or were missing were censored in all cases.

Egg-laying assays

Egg-laying assays were performed at 20°C. In brief, N2 animals were synchronized as L1 larvae by filtering [11 µm filter; (Larance et al. 2011)] and at L4, 30 animals were transferred onto fresh 6 cm NGM/OP50 plates at 3 animals per plate. 24 h post L4 (day 1), the adults were transferred onto fresh NGM/OP50 plates and this was repeated every 24 h until no more eggs were laid. On each day, the plates containing laid eggs and/or L1 larvae (from which the adults were transferred onto new plates) were incubated at 25°C overnight, followed by a 30 min incubation at 4°C to temporarily immobilize the animals. Dead eggs and larvae (viable eggs) were scored. For FUdR egg-laying assays, FUdR treatment was initiated at L4 and adults were transferred onto fresh NGM/OP50 plates containing FUdR on each day of adulthood. *prx-5* RNAi treatment was initiated at day 0 of adulthood, as described previously (Zhou et al. 2012) and adults were transferred onto fresh RNAi plates on each day of adulthood.

Determination of class A, B, C animals

Class A, B, C animals were identified as described previously (Herndon et al. 2002). Measurements were made at 20°C using N2 (Bristol) animals. Experiments were performed in a similar manner to lifespan assays (see above) although no FUdR was used, and animals were scored as being class A, B or C on every other day of adulthood. Exploded, bagged or missing animals were censored. In brief, animals that moved well were scored as Class A, animals showing uncoordinated movements were scored as Class B, and animals that showed little or no movement were scored as Class C.

Pumping assays

Synchronized single N2 (Bristol) animals maintained at 20°C were observed. On each day of adulthood, the number of contractions in the terminal bulb of the pharynx was counted for 3 x 30 s intervals (n = 20 or as indicated in the figure legends).

Microscopy and imaging

Induction of GFP-SKL expression from the *hsp-16.2* promoter was carried out as follows: day-1 adults (or as indicated in the figure legends) grown at 20°C were shifted for 2 h to 34°C, followed by 30 min at 20°C, then 1 h at 34°C, after which the worms were returned to 20°C for 12 h. Animals were

anesthetized on 2% agarose pads in 10 mM levamisole for no more than 10 min immediately prior to viewing. Imaging of GFP-SKL strains (intestinal cells) was performed using an UltraVIEW Vox CSU-X1 confocal imaging system (Perkin Elmer), incorporating an Olympus IX70 microscope, a 488 nm solid-state laser, and an Olympus 60X/1.3 NA oil-immersion objective. Images were processed using Volocity 15 software (Perkin Elmer) and analyzed using Cell Profiler (Lamprecht et al. 2007) as follows: z-sections were taken over 40 µm at 0.5 µm intervals. The 81 resulting images per worm were quantified individually for peroxisomes (using a modification of the speckle counting tool in Cell Profiler) and cytosol (total GFP intensity minus peroxisomes). Mean intensities from each section were then summed to get total intensity, peroxisomal intensity and cytosolic intensity per worm. These were then graphed using R. Additionally, the number of peroxisomes was counted and graphed from the sum of the 81 z-stacks as well as from the single z-section that had the maximum number of peroxisomes. All other microscopy was performed using a Leica MZ16FA microscope (Leica; Bannockburn, IL) and images were analyzed using Cell Profiler. Here, fluorescence intensity in the whole animal was measured and subtracted from background fluorescence (i.e. mean fluorescence) outside the worm and plotted using R.

SILAC labeling, sample preparation, fractionation and clean-up

For isotopic labeling, N2 (Bristol) worms were grown on NGM-N plates at 20°C as described previously (Larance et al. 2011) with the following adaptations: 25 mid-late L4 larvae were picked on to NMG-N plates supplemented with 1 mM IPTG and 1 µg/ml carbenicillin, and seeded with *E. coli* SLE1 grown in light, medium or heavy label as described previously (Larance et al. 2011). Labeling was for 2 generations at 20°C. L1 animals from the F2 generation were collected by filtering through an 11 µm filter (Millipore) and 40,000 L1 larvae per condition (light, medium or heavy) were transferred to 9 cm NGM-N plates seeded with labeled SLE1 at 4,000 worms per plate. Approximately 2 days later, when the worms were mid-late L4 animals, they were washed off the plates with M9 buffer and transferred to fresh NGM-N plates (seeded with the required labeled bacteria) containing 50 µM FUdR. Care was taken to ensure the animals did not starve during the experiment – if required, additional concentrated, labeled SLE1 was added to the plates. The animals were then collected as required (Heavy – Day 1 adults, Medium – Day 5 and Light – Day 10) by washing off the plate with M9 buffer, followed by a further 3-5 washes in M9 to remove bacteria and eggs. Worms were collected by light centrifugation or by gravity at room temperature (5-10 min in a falcon tube on the bench). Worm pellets were snap-frozen in liquid N_2 and upon thawing, were mixed at a 1:1:1 ratio by volume of heavy:medium:light. 5% of the mixture was lysed directly in SDS buffer (4% SDS, 150 mM NaCl, 10 mM phosphate buffer pH 7, 50 mM N-ethylmaleimide, 25 mM TCEP) as follows: 2x volume of buffer and 1x volume of 0.7 mM Zirconia beads (BioSpec) were added and the sample was lysed by bead beating (Mini Beadbeater 8, BioSpec) at max speed for 3 x 20 s with 20 s intervals. The sample was then heated to 65°C for 10 min, centrifuged at 16,100 g for 15 min at room temperature and the supernatant collected. The remainder of the worm mixture (95%) was fractionated using the Q-Proteome Cell Compartment Kit (Qiagen) as described previously (Pourkarimi et al. 2012), but with the following modifications: (1) worm pellets were aliquotted into 100 µl aliquots and each aliquot was processed separately; (2) 100 µl 0.7 mM Zirconia beads were used per aliquot, together with 250 µl buffer CE1; subsequent buffers were scaled accordingly, maintaining the ratios recommended by the manufacturer; (3) homogenizing using a Qiashredder (Qiagen) was adjusted as follows: the sample was added to a shredder and centrifuged for 10 s at 100 g, after which the eluate was transferred to a fresh shredder and the process repeated. SDS, TCEP and NEM were added to each fraction, to final concentrations of 2%, 25 mM and 50 mM respectively, following which the samples were heated at 65°C for 10 min. Equal amounts of protein (500 µg, measured by EZQ Assay) from the SDS-lysate and sub-cellular fractions were concentrated by chloroform-methanol precipitation and resuspended in 8 M urea in 100 mM triethylammonium bicarbonate (TEAB). Samples were diluted 8-fold using 100 mM TEAB and supplemented with $CaCl_2$ to a final concentration of 1 mM. Trypsin was added at a 1:50 ratio of enzyme to protein and incubated for 16 h at 37°C. Sample desalting and offline SAX chromatography was performed as described previously (Larance et al. 2015; Ly et al. 2014).

LC-MS/MS and analysis of spectra

~1 µg of each desalted SAX fraction containing peptides in 5% (vol/vol) formic acid (~10 µl) was injected onto a 0.3 mm id × 5 mm PepMap-C18 pre-column and chromatographed on a 75 µm × 15 cm PepMap-C18 column. Using the following mobile phases: 2% ACN incorporating 0.1% FA (Solvent A) and 80% ACN incorporating 0.1% FA (Solvent B), peptides were resolved using a linear gradient from 5% B to 35% B over 4 hours with a constant flow of 200 nL min^{-1} as described previously (Ly et al. 2014). Peptides were ionized by electrospray ionization at +2.0 kV. Tandem mass spectrometry analysis was carried out on a Q-Exactive mass spectrometer (Thermo Fisher Scientific) using HCD

fragmentation. The data-dependent acquisition method used acquired MS/MS spectra on the top 10 most abundant ions at any one point during the gradient. The raw data produced by the mass spectrometer were analysed using the quantitative proteomics software MaxQuant including the Andromeda search engine (http://www.maxquant.org, version 1.4.1.2) (Cox & Mann 2008). Peptide and protein level identification were both set to a false discovery rate of 1% using a target-decoy based strategy. The databases supplied to the search engine for peptide identifications were both the *C. elegans* (December 2013) and *E. coli* Uniprot databases (July 2012) containing 22,998 and 4,431 entries respectively. The mass tolerance was set to 7ppm for precursor ions and MS/MS mass tolerance was set at 20 ppm. Enzyme was set to trypsin/p with up to 2 missed cleavages. Deamidation of Asn and Gln, oxidation of Met, pyro-Glu (with N-term Gln), and acetylation of the protein N-terminus were set as variable modifications. N-ethylmaleimide on Cys was searched as a fixed modification. The output from MaxQuant provided peptide level data as well as protein level data, grouped by protein isoforms. Protein groups were defined as a set of unique peptides that are either shared between multiple protein isoforms, or belong to a single protein isoform (Figure S3). While this may lead to higher redundancy it enables us to quantify changes in individual protein isoforms.
All raw proteomics data from this study have been deposited to the ProteomeXchange Consortium (http://proteomecentral.proteomexchange.org) via the MassIVE partner repository with the dataset identifiers PXD004584 (ProteomeXchange) and MSV000079263 (MassIVE). The processed data are additionally provided in a searchable, online database (https://www.peptracker.com/epd/).

Data analysis, statistics, homology mapping and graphing
Most of the subsequent data analysis was done using R version 3.1.3 (R Core Team n.d.) employing Rstudio 0.98.1091 (RStudio Team n.d.) and the ggplot2 package for generating graphs (Wickham 2009). The MaxQuant output file "evidence.txt" was used to compile protein groups as depicted in Figure S3; the MaxQuant "proteinGroups.txt" output file was used for analysis of the sub-cellular fractionation data presented in Figure S2 (see Table S1). Only those proteins identified in all 3 biological replicates with at least 2 razor+unique peptides were included in our "quantified proteins" dataset (Table S3). As measurement error correlates inversely with intensity, prior to p-value calculations by ANOVA, the dataset was grouped into three bins based on protein abundance (i.e. measured intensity). Control proteins for each ANOVA calculation were randomly sampled from a set of age-invariant proteins (with SILAC pair ratios or $\log_2$ fold changes ≈ 0) in the same abundance bin as the protein in consideration. The random sampling and ANOVA calculation for each protein was looped 100 times and used to estimate the false discovery rate (FDR). GO-term enrichment was performed using DAVID (Huang, Sherman & Lempicki 2009a; Huang, Sherman & Lempicki 2009b) unless otherwise indicated, with the full *C. elegans* proteome supplied by DAVID used as the background list, and plotted to reduce redundancy using ReviGo (Supek et al. 2011); Venn diagrams were generated using BioVenn (Hulsen et al. 2008). PCA analysis was done using Partek Genomics Suite (Partek Inc, St. Louis, MO). GSEA analysis was done using the algorithm from the Broad Institute (Subramanian et al. 2005) using either a user-defined gene set or the KEGG pathway database (Kanehisa et al. 2014; Kanehisa & Goto 2000). Human homologs of *C. elegans* genes were obtained using information from the WormBase FTP site (ftp://ftp.wormbase.org/pub/wormbase/); files containing both best BLAST hits and orthologs were used and merged into a single file using R. Microscopy images were analyzed and quantified using Cell Profiler (Lamprecht et al. 2007).

SUPPLEMENTAL REFERENCES

Cox, J. & Mann, M., 2008. MaxQuant enables high peptide identification rates, individualized p.p.b.-range mass accuracies and proteome-wide protein quantification. *Nature biotechnology*, 26(12), pp.1367–1372.

Dillin, A., Crawford, D.K. & Kenyon, C., 2002. Timing requirements for insulin/IGF-1 signaling in C. elegans. *Science (New York, N.Y.)*, 298(5594), pp.830–834.

Herndon, L.A. et al., 2002. Stochastic and genetic factors influence tissue-specific decline in ageing C. elegans. *Nature*, 419(6909), pp.808–814.

Hsin, H. & Kenyon, C., 1999. Signals from the reproductive system regulate the lifespan of C. elegans. *Nature*, 399(6734), pp.362–366.

Huang, D.W., Sherman, B.T. & Lempicki, R.A., 2009a. Bioinformatics enrichment tools: paths toward the comprehensive functional analysis of large gene lists. *Nucleic acids research*, 37(1), pp.1–13.

Huang, D.W., Sherman, B.T. & Lempicki, R.A., 2009b. Systematic and integrative analysis of large gene lists using DAVID bioinformatics resources. *Nature protocols*, 4(1), pp.44–57.

Hulsen, T., de Vlieg, J. & Alkema, W., 2008. BioVenn – a web application for the comparison and visualization of biological lists using area-proportional Venn diagrams. *BMC Genomics*, 9(1), p.488.

Kamath, R.S. et al., 2001. Effectiveness of specific RNA-mediated interference through ingested double-stranded RNA in Caenorhabditis elegans. *Genome biology*, 2(1), p.RESEARCH0002.

Kanehisa, M. & Goto, S., 2000. KEGG: kyoto encyclopedia of genes and genomes. *Nucleic acids research*, 28(1), pp.27–30.

Kanehisa, M. et al., 2014. Data, information, knowledge and principle: back to metabolism in KEGG. *Nucleic acids research*, 42(Database issue), pp.D199–205.

Lamprecht, M.R., Sabatini, D.M. & Carpenter, A.E., 2007. CellProfiler™: free, versatile software for automated biological image analysis. *Biotechniques*.

Larance, M. et al., 2015. Global proteomics analysis of the response to starvation in C. elegans. *Molecular & cellular proteomics : MCP*, 14(7), pp.mcp.M114.044289–2001.

Larance, M. et al., 2011. Stable-isotope labeling with amino acids in nematodes. *Nature methods*, 8(10), pp.849–851.

Ly, T. et al., 2014. A proteomic chronology of gene expression through the cell cycle in human myeloid leukemia cells J. Pines, ed. *eLife*, 3, p.e01630.

Pourkarimi, E., Greiss, S. & Gartner, A., 2012. Evidence that CED-9|[sol]|Bcl2 and CED-4|[sol]|Apaf-1 localization is not consistent with the current model for C. elegans apoptosis induction. *Cell Death & Differentiation*, 19(3), pp.406–415.

R Core Team, R: A Language and Environment for Statistical Computing. Available at: http://www.R-project.org/.

RStudio Team, RStudio: Integrated Development Environment for R. Available at: http://www.rstudio.com/.

Subramanian, A. et al., 2005. Gene set enrichment analysis: a knowledge-based approach for interpreting genome-wide expression profiles. *Proceedings of the National Academy of Sciences of the United States of America*, 102(43), pp.15545–15550.

Supek, F. et al., 2011. REVIGO summarizes and visualizes long lists of gene ontology terms. C. Gibas, ed. *PloS one*, 6(7), p.e21800.

Wickham, H., 2009. *ggplot2: elegant graphics for data analysis*, New York, NY: Springer New York. Available at: http://had.co.nz/ggplot2/book.

Zhou, B. et al., 2012. Midlife gene expressions identify modulators of aging through dietary interventions. *Proceedings of the National Academy of Sciences of the United States of America*, 109(19), pp.E1201–9.
