# Supplementary material for: Deep Proteome Analysis Identifies Age-Related Processes in C. elegans
Source: Cell Syst. 2016 Aug 24;3(2):144–59. doi: 10.1016/j.cels.2016.06.011 (PMC5003814; doi:10.1016/j.cels.2016.06.011)
Supplement: Document S2. Article plus Supplemental Information [file mmc6.pdf]

Cell Systems

Article

Deep Proteome Analysis Identifies Age-Related Processes in *C. elegans*

Graphical Abstract

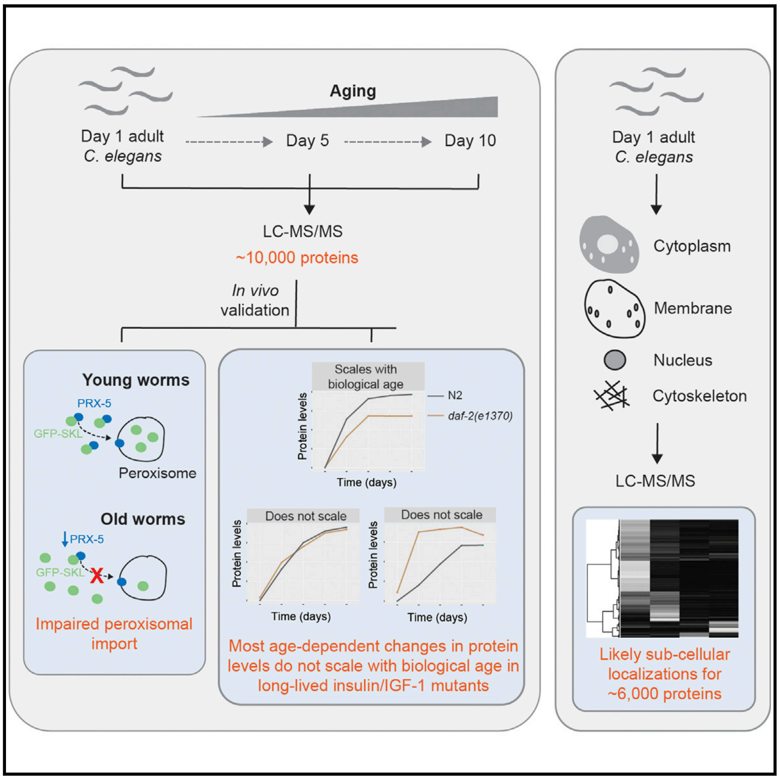

Highlights

- Near doubling in *C. elegans* proteome coverage with age-dependent protein measurements
- Putative sub-cellular localization assignments for >6,000 nematode proteins
- Peroxisome protein import impaired during aging
- Most age-variant proteins do not scale with biological age in insulin/IGF-1 mutants

Authors

Vikram Narayan, Tony Ly, Ehsan Pourkarimi, Alejandro Brenes Murillo, Anton Gartner, Angus I. Lamond, Cynthia Kenyon

Correspondence

cynthia@calicolabs.com

In Brief

Deep proteome analysis of adult *C. elegans* provides a valuable resource, reveals a failure in peroxisome protein import during aging, and, surprisingly, shows that the rates of change in the levels of the majority of age-variant proteins do not scale with the reduced rate of biological aging in long-lived insulin/IGF-1 mutants.

Accession Numbers

PXD004584

Narayan et al., 2016, Cell Systems *3*, 144–159
August 24, 2016 © 2016 The Authors. Published by Elsevier Inc.
http://dx.doi.org/10.1016/j.cels.2016.06.011

OPEN ACCESS
CellPress

Cell Systems
Article

Deep Proteome Analysis Identifies Age-Related Processes in *C. elegans*

Vikram Narayan,[1,2,3] Tony Ly,[1] Ehsan Pourkarimi,[1,5] Alejandro Brenes Murillo,[1] Anton Gartner,[1,4] Angus I. Lamond,[1,4] and Cynthia Kenyon[2,3,4,*]
[1]Centre for Gene Regulation and Expression, School of Life Sciences, University of Dundee, Dundee DD1 5EH, UK
[2]Department of Biochemistry and Biophysics, University of California, San Francisco, San Francisco, CA 94158-2517, USA
[3]Calico Life Sciences, 1170 Veterans Boulevard, South San Francisco, CA 94080, USA
[4]Co-senior author
[5]Present address: Memorial Sloan Kettering Cancer Center, New York, NY 10065, USA
*Correspondence: cynthia@calicolabs.com
http://dx.doi.org/10.1016/j.cels.2016.06.011

SUMMARY

Effective network analysis of protein data requires high-quality proteomic datasets. Here, we report a near doubling in coverage of the *C. elegans* adult proteome, identifying >11,000 proteins in total with ~9,400 proteins reproducibly detected in three biological replicates. Using quantitative mass spectrometry, we identify proteins whose abundances vary with age, revealing a concerted downregulation of proteins involved in specific metabolic pathways and upregulation of cellular stress responses with advancing age. Among these are ~30 peroxisomal proteins, including the PRX-5/PEX5 import protein. Functional experiments confirm that protein import into the peroxisome is compromised in vivo in old animals. We also studied the behavior of the set of age-variant proteins in chronologically age-matched, long-lived *daf-2* insulin/IGF-1-pathway mutants. Unexpectedly, the levels of many of these age-variant proteins did not scale with extended lifespan. This indicates that, despite their youthful appearance and extended lifespans, not all aspects of aging are reset in these long-lived mutants.

INTRODUCTION

The nematode *C. elegans* has been used widely to study aging due to its short lifespan and hermaphroditic reproductive cycle, as a result of which large numbers of isogenic animals can be cultured easily. Much of what we know about how aging is regulated was discovered through genetic perturbations in *C. elegans*. For example, lifespan extension through downregulation of insulin/IGF-1 signaling, and later TOR, was first described in these animals (Kenyon et al., 1993; Vellai et al., 2003; Kimura et al., 1997). Since then, dozens of genes, primarily affecting the sensation of, or response to, stress and nutrient sensing, have been found to affect lifespan. Many of these genes also are associated with aging in other organisms, including mammals (Kapahi et al., 2010; Kenyon, 2010).

In comparison with detailed studies on the genetic control of aging, relatively less is known about how the proteome changes with age. There is evidence for increased protein aggregation and a gradual failure of the ubiquitin-proteasome machinery with advancing age, leading to a disruption of proteostasis (David et al., 2010; Taylor and Dillin, 2011; Morley and Morimoto, 2004). Only a handful of proteomic-based aging studies have been reported to date, so far with limited global protein coverage and resolution. Recently, the opportunity to apply quantitative proteomic approaches in *C. elegans* has been aided by the development of a stable-isotope labeling with amino acids in cell culture (SILAC)-based methodology for nematodes (Larance et al., 2011; Fredens et al., 2011). A database of mRNA and protein-abundance changes during nematode development also has been compiled (Grün et al., 2014) and age-dependent changes measured (Walther et al., 2015; Liang et al., 2014; Dong et al., 2007; Zimmerman et al., 2015; Copes et al., 2015).

Consistent with the measurement of transcript and protein levels during development (Grün et al., 2014), recent findings in worms have reported an unexpectedly weak correlation between mRNA-level changes and protein-level changes during aging, suggesting that a significant proportion of age-related changes in protein levels are regulated at the post-translational level (Walther et al., 2015). However, current reports quantify only about 25% of the theoretical proteome, i.e., 5,000 proteins of ~20,000 protein-coding genes, although not every protein is likely to be expressed at all times. Together, these findings highlight the need to measure protein-level changes, and they underscore the importance of expanding the depth of protein coverage to provide a better understanding of aging. Having in-depth, high-quality proteomic datasets also will expand the number of validated protein-coding genes, and it will aid in the development of novel network analysis tools based on proteins, rather than mRNAs.

In this study, we expand the coverage and quantification of the *C. elegans* proteome, identifying >9,300 protein groups in each of three biological replicates. We characterize how organismal aging affects the levels of a large subset of these proteins (7,380), providing a proteomic fingerprint of the aging process. This expanded analysis of the proteome identifies a large set of proteins whose abundances change with age. These data, coupled with in vivo biological experiments, also identify the

This is an open access article under the CC BY license (http://creativecommons.org/licenses/by/4.0/).

OPEN ACCESS
CellPress

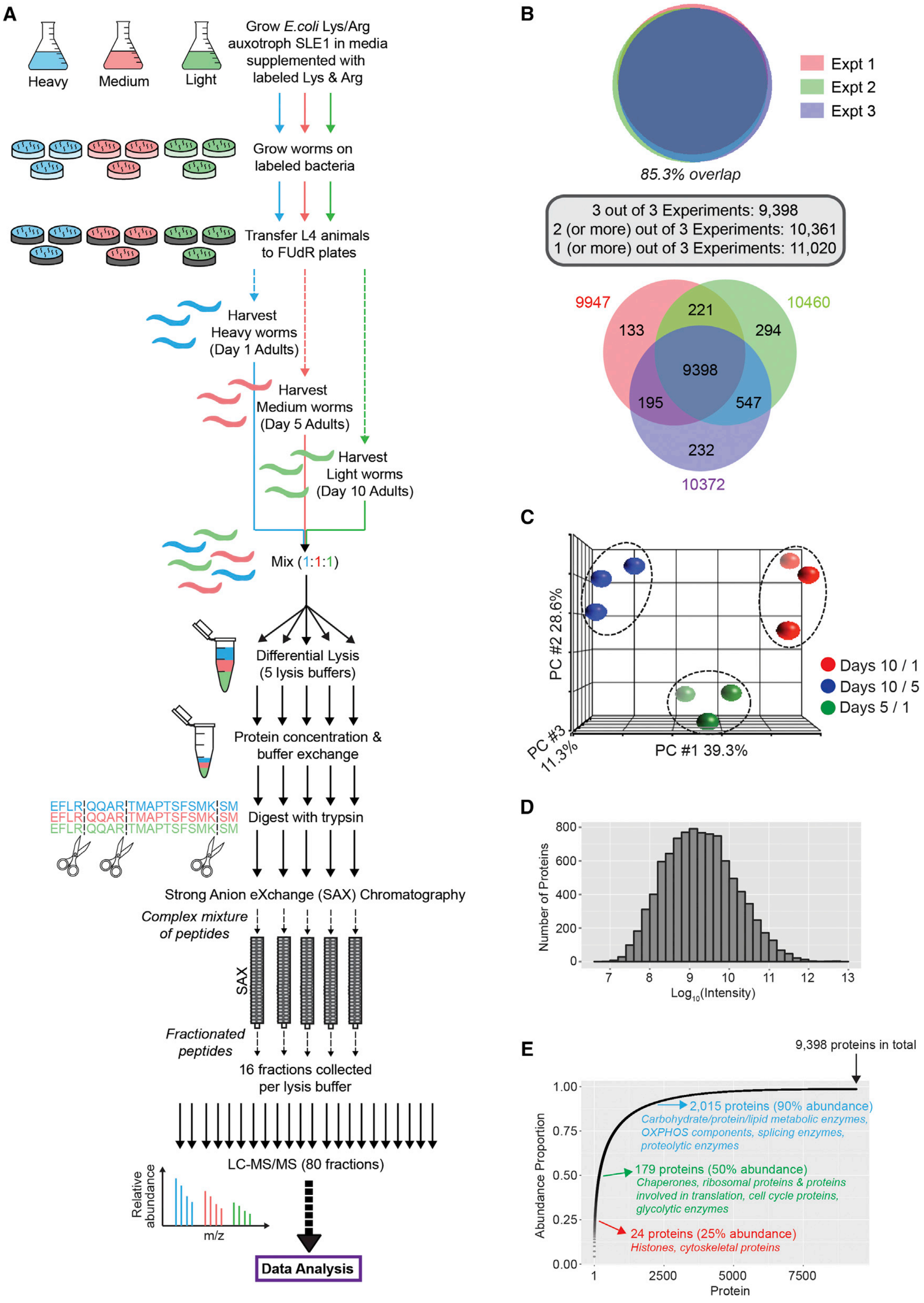

Figure 1. SILAC-Based Deep Proteome Analysis Reproducibly Identifies 9,398 Proteins in *C. elegans*

(A) Schematic overview shows the methodology used in this study.

(B) Area-proportionate Venn diagram (top) shows reproducibility in protein identifications across three biological replicates, with the number of proteins in each region of the Venn diagram indicated on a schematic (bottom).

(legend continued on next page)

concerted downregulation of specific cellular metabolic networks during aging and uncover an age-dependent dysregulation of the peroxisome. In addition, combining our new findings with a detailed analysis of previously published datasets shows that only a subset of proteins whose abundances change with age in wild-type animals scale with the rate of biological aging in long- and short-lived insulin/IGF-1 pathway mutants.

RESULTS

Deep Proteome Analysis in *C. elegans* Reproducibly Identifies 9,398 Proteins

To make global measurements of age-related changes in protein abundance, we first developed a workflow to improve proteome coverage in *C. elegans*, while ensuring accurate quantification. We used the recently described SILAC-for-nematodes strategy, in which worms are fed bacteria whose proteins are labeled with arginine and lysine containing either light, medium, or heavy isotopes, resulting in near-complete labeling of the worm proteome after two generations (Larance et al., 2011; Fredens et al., 2011). This technique also exploits an RNAi-via-feeding strategy (Timmons and Fire, 1998) to knock down *orn-1*, thus preventing ORN-1-catalyzed arginine-to-proline conversion (Larance et al., 2011). We confirmed that *orn-1* RNAi had no significant effect on *C. elegans* lifespan (Figure S1A). Stable-isotope-labeled nematodes were harvested at days 1 (fertile young adults), 5 (early aging/post-reproductive), and 10 (aging) of adulthood (Figures 1A and S1B). Time points after day 10 of adulthood were not considered because *C. elegans* aging has a stochastic component that becomes particularly evident after this time (Herndon et al., 2002; Figure S1C). Importantly, although wild-type worms can live for up to 4 weeks, many phenotypes associated with aging, such as decreased rates of pharyngeal pumping, mitochondrial fission, and muscle deterioration, are visible at day 10 (Jiang et al., 2015; Croll et al., 1977; Huang et al., 2004; Garigan et al., 2002; Figure S1D).

As this study includes time points at which worms are fertile, in order to eliminate progeny from our assay, labeled worms were treated with 50 μM 5-Fluoro-2′-deoxyuridine (FUdR) at the mid-late L4 larval stage. We found that 100% of the progeny from worms treated with 50 μM FUdR were unviable, and that treated worms also had fewer eggs, thus decreasing egg-protein contamination in the assay (Figure S1E). Notably, unlike animals treated with higher doses of FUdR, at the chosen dose, animals were healthy, not prone to bursting, and, importantly, had a lifespan similar to that of untreated control animals (Figure S1F). The FUdR-treated labeled worms that were harvested at days 1, 5, and 10 of adulthood were next mixed and lysed in different buffers to increase proteome coverage (Figure 1A). Following digestion with trypsin, the resulting peptides were fractionated by hydrophilic strong anion exchange (SAX) chromatography to further reduce sample complexity, as described previously (Ly et al., 2014), prior to analysis by liquid chromatography-tandem mass spectrometry (LC-MS/MS). Three independent biological replicates were analyzed.

We used a differential buffer extraction methodology (QProteome Cell Compartment Kit, QIAGEN), which allowed us to sample a wider pool of proteins with different physicochemical properties (Figure S2A; Pourkarimi et al., 2012). This method also increased the number of unique peptides we identified from a single protein when the data were aggregated, thereby improving sequence coverage (Figure S2B). Additionally, we found that, although the QProteome kit was designed to work primarily with cells grown in culture, with our optimizations, the kit could be used successfully to isolate proteins from different sub-cellular compartments in day 1 adult worms, in spite of the presence of a cuticle and syncytium (Figures S2C–S2E). The validity of our method was supported by the predicted fractionation of proteins that previously have been shown to localize to specific cellular locations (Figures S2D and S2E).

Using this method, we compiled a resource with putative sub-cellular localization profiles for ~6,300 *C. elegans* proteins, based on our proteomic analysis of day 1 adult nematodes treated with FUdR (Table S1). The relative proportions of these proteins in four sub-cellular compartments—i.e., cytoplasm, membrane (plasma membrane and membrane-bound organelles, excluding the nucleus), nucleus, and cytoskeleton/insoluble fractions—are presented, together with lists of proteins that were predominantly localized to any of these fractions based on our proteomic analysis (Table S1). This is a significant improvement over the 4,954 protein-coding nematode genes (of ~20,000) that currently have a manually curated gene ontology (GO) term cellular component annotation, of which only a small subset (~2,200 genes) is based on experimental evidence (Figures S2F–S2H; GO annotations and associated data were obtained from WormBase). As worm tissue integrity is compromised during aging (Garigan et al., 2002) and we were unable to confirm the efficacy of the kit in isolating sub-cellular fractions from old nematodes, we were hesitant to make any conclusions about changes in protein localization occurring during aging based on these data.

Using this combination of protein-level and peptide-level fractionation, >125,000 sequence-unique peptides were identified with a false discovery rate (FDR) of 1%. These were assembled into >9,300 protein groups that were detected in all three biological replicates (see the Supplemental Experimental Procedures; Table S2; Figure S3). Identifications were highly reproducible, with 10,361 protein groups identified in at least two of three biological replicates and 9,398 protein groups identified across all three biological replicates (Figure 1B). Relative protein quantification among the three biological replicates also was very reproducible, as shown in the principal-component analysis (PCA) plot (Figure 1C) and using Pearson's correlation (median value for Pearson's r across the various pairwise comparisons was

(C) Principal-component analysis (PCA) plot shows reproducibility in protein quantification across the three biological replicates.
(D) Histogram shows $\log_{10}$-transformed protein abundance (MaxQuant intensity) for day 1 measurements.
(E) A cumulative plot of protein abundance, as estimated using median protein intensity measurements based on three biological replicates. 9,398 proteins were identified in total with at least one peptide per protein. 90% of the bulk protein mass is made up of only 2,015 proteins (21% of measured proteins). The remaining protein identifications (7,383 or 79%) comprise less than 10% of the bulk protein mass.
See also Figures S1–S4 and S8.

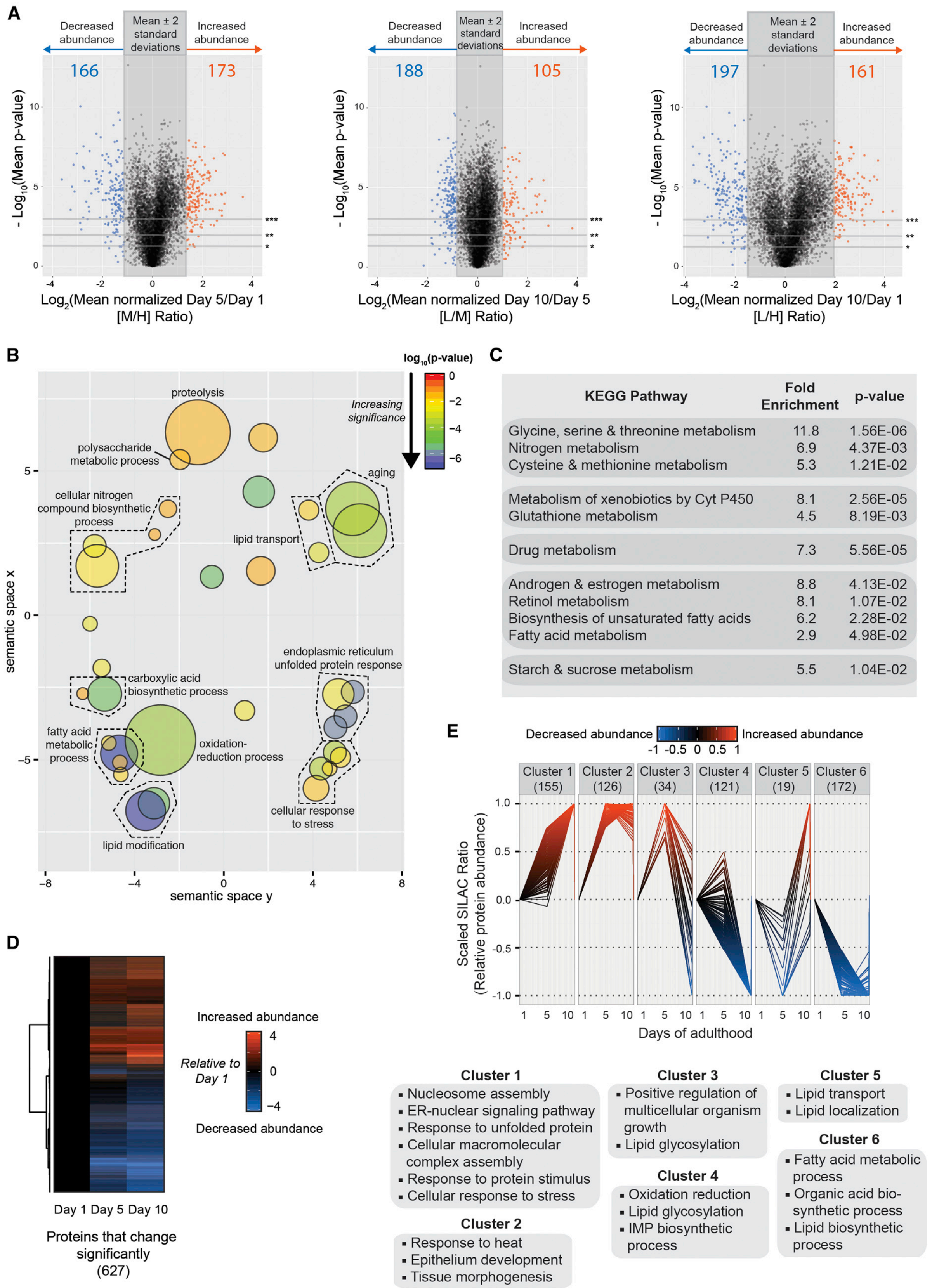

KEGG Pathway	Fold Enrichment	p-value
Glycine, serine & threonine metabolism	11.8	1.56E-06
Nitrogen metabolism	6.9	4.37E-03
Cysteine & methionine metabolism	5.3	1.21E-02
Metabolism of xenobiotics by Cyt P450	8.1	2.56E-05
Glutathione metabolism	4.5	8.19E-03
Drug metabolism	7.3	5.56E-05
Androgen & estrogen metabolism	8.8	4.13E-02
Retinol metabolism	8.1	1.07E-02
Biosynthesis of unsaturated fatty acids	6.2	2.28E-02
Fatty acid metabolism	2.9	4.98E-02
Starch & sucrose metabolism	5.5	1.04E-02

(legend on next page)

0.85; Figure S4A). The measured protein intensities spanned approximately seven orders of magnitude (Figure 1D), and mean protein sequence coverage was ~30%.

Interestingly, the abundance composition of the *C. elegans* proteome is dominated by a relatively small proportion of proteins (Figure 1E; 24 proteins make up 25% of the measured abundance or total protein content), with apparent saturation at ~4,000 proteins. Protein- and peptide-level fractionation enabled access to a much deeper proportion of the proteome, i.e., ~7,383 proteins that cumulatively represent only 10% of the measured abundance. The total of 11,020 protein groups identified in at least one of the three biological replicates (see Figure 1B) corresponds to ~54% of the predicted *C. elegans* proteome as defined by WormBase (Release WS243, March 28, 2014, containing 20,480 protein-coding genes; Figure S4B). Of the 11,020 protein groups, 6,480 proteins were reproducibly identified with sequence-unique peptides (Figure S4B). Where peptides could not be assigned to a single protein unambiguously (e.g., alternatively spliced isoforms and proteins encoded by genes with high sequence similarity), the lead member of the assembled protein group, as determined by Occam's razor rule using MaxQuant (Cox and Mann, 2008), was used for all downstream data analysis.

Our dataset corresponds to ~22% of the theoretical *C. elegans* MS-flyable peptide digest of the predicted proteome (Figure S4B). However, both the 54% overlap of our dataset with the theoretical proteome and 22% overlap with the theoretical peptide-digest are likely to be underestimated, as our study only used adult *C. elegans*. Indeed, a recent study identified only ~3,000 proteins in young-adult nematodes (compared to ~10,000 in our study) but ~9,000 when developmental stages (embryo, L1, L2, L3, early L4, late L4, and young adult) were included (Grün et al., 2014). Similarly, ~11,000 proteins were identified from a mixed-developmental stage worm population in an independent study (Schrimpf et al., 2009), although the study additionally reported a very high FDR (~7%), a large number of single-peptide-based protein identifications (~23% of the dataset), and an absence of biological replicates.

Of the Adult *C. elegans* Proteome, ~8% Changes Strikingly during Aging

Of the 9,398 protein groups identified in all three biological replicates, those with at least two sequence-unique peptides in each replicate (7,380) were quantified across the day 1, 5, and 10 time points (Figure S3; Table S3). The 95% confidence intervals were defined for each SILAC pair (i.e., day 5/day 1 [M/H] ratio, day 10/day 5 [L/M] ratio, and day 10/day 1 [L/H] ratio) by calculating mean ratio ±2 SDs across the dataset. To compute a statistic for data reproducibility and significance, p values were calculated using ANOVA. Age-invariant control proteins with $\log_2$ SILAC pair ratios close to 0 were randomly sampled from the dataset for the ANOVA calculation. Additionally, as measurement error correlates inversely with intensity, the random sampling of controls was controlled for intensity (see the Supplemental Experimental Procedures and Figure S3 for details). Volcano plots show the FDR-corrected p value from ANOVA for each protein group against the respective $\log_2$(fold change) for each SILAC pair (Figure 2A). The p values less than 0.05 (95% cutoff) were considered significant, and 627 proteins were found to be significantly altered in abundance across the three time points considered (Table S4). The vast majority of these age-altered proteins had p values < 0.001.

Specific Metabolic Processes and Stress Responses Are Preferentially Affected during Aging

GO term analysis for biological process was performed on the 627 proteins showing the most significant change in abundance, during the three time points measured, to profile nematode aging, using the Database for Annotation, Visualization and Integrated Discovery (DAVID; Huang et al., 2009) and ReviGo (Supek et al., 2011). This analysis revealed an enrichment of GO terms including aging itself, fatty acid metabolic processes, oxidation-reduction processes, unfolded protein response, and cellular response to stress (Figure 2B). Additionally, a highly significant enrichment of the GO term lipid modification also was observed, together with lipid transport and metabolic processes such as polysaccharide metabolism and cellular nitrogen compound biosynthesis (Figure 2B). Similar results were obtained upon analyzing the same 627 proteins for Kyoto Encyclopedia of Genes and Genomes (KEGG) pathway enrichment (Kanehisa and Goto, 2000; Kanehisa et al., 2014) using DAVID. Thus, enrichment of cellular metabolic pathways, including amino acid metabolism, fatty acid metabolism, and carbohydrate metabolism, was observed (Figure 2C), along with cellular stress responses. As GO term enrichment analysis does not take into account the direction of changes (i.e., whether a network is up- or downregulated), we further probed our dataset using gene set enrichment analysis (GSEA) as described below.

We generated a heatmap of the 627 proteins whose levels showed the most significant change across the three time points examined to assess whether groups of proteins show similar expression profiles during aging (Figure 2D). The heatmap

Figure 2. Of the Detected *C. elegans* Proteome, 8.5% Changes by a Large Magnitude during Aging

(A) Volcano plot of $\log_2$-transformed SILAC ratios (fold change) against the negative $\log_{10}$ of the FDR-corrected p value calculated using ANOVA. Shown are the graphs for day 5 versus day 1 fold changes (left), day 10 versus day 5 (center), and day 10 versus day 1 (right). The shaded dark gray box represents the mean ± 1.96 SD for each plot. Horizontal gray lines denote p value cutoffs of 0.05 (*), 0.01 (**), and 0.001 (***). The number of proteins that increase (orange) or decrease (blue) significantly (p value < 0.05) in each graph also is indicated.

(B) GO term enrichment (biological process) of the 627 age-variant proteins (blue and orange dots from the three volcano plots in A with p values < 0.05) performed using DAVID and plotted using REVIGO. The size of the bubbles is indicative of the number of proteins annotated with that GO term; bubbles are color coded according to significance.

(C) KEGG pathway enrichment of the 627 age-variant proteins calculated using DAVID is shown.

(D) Heatmap shows age-dependent changes in abundance of the above proteins generated using R.

(E) Hierarchical clustering of the 627 proteins into six groups based on trend profiles. Results from GO term enrichment analysis (p < 0.05) of each cluster using DAVID also are depicted. Only GO terms in clusters 1, 4, 5, and 6 remained significant (p < 0.05) after multiple hypothesis correction (Benjamini).

See also Figure S4.

groups proteins that showed similar abundance changes during adulthood, which were then further clustered using an unbiased hierarchical clustering approach. To minimize redundancy in clustering due to large differences in the magnitude of the abundance changes, the SILAC ratios were scaled to range between −1 (maximum decrease) and +1 (maximum increase), with ratios near 0 indicating no change in abundance with age. Using hierarchical clustering based on Ward's minimum variance method (Ward, 2012), we grouped the age-variant proteins into clusters based on their abundance trend profiles.

Although two clusters (proteins that increase with age and proteins that decrease with age; Figures S4C and S4D) may be used to categorize this set of 627 proteins, we found that grouping the proteins into six clusters could be more biologically meaningful, although one must be cautious in interpreting these data as only three time points were considered in this study (Figures 2E, S4C, and S4D). For example, cluster 3 in the six-cluster model, which includes 34 proteins that increase sharply in abundance between days 1 and 5 of adulthood and then decrease sharply between days 5 and 10, is enriched for the GO term lipid glycosylation (Figure 2E). Thus, it will be interesting to carry out follow-up biological experiments to examine whether lipid glycosylation or other lipid modifications decrease during aging and to study the functional consequences of this decrease. In the two-cluster model, this small group of proteins is grouped together with a larger group of proteins whose levels increase during days 1–5 and then continue to increase between days 5 and 10, and the GO term lipid glycosylation is not significantly enriched (Figure S4D).

We next extended our analysis to include our entire aging dataset, rather than just the subset of 627 proteins that showed the largest fold change during the three time points, using GSEA (Subramanian et al., 2005). The advantage of this type of analysis is that, in addition to genes (or proteins) associated with gross changes in abundance that are usually defined by arbitrary fold-change cutoffs, GSEA also detects small but reproducible changes in levels of RNAs or proteins, provided a similar trend is observed in multiple constituents of a defined gene set or pathway. Because GSEA, in common with the majority of pathway analysis tools, was designed to work primarily with human genes rather than nematode genes, we first probed the dataset using two nematode gene sets that we compiled, using information from WormBook, WormBase, and the literature (Kenyon, 2010; Murphy and Hu, 2013; Lapierre and Hansen, 2012), for changes in networks known to be associated with aging in *C. elegans*. We could reproducibly detect and quantify known *C. elegans* components of the TORC1 and TORC2 complexes and 17 components of the insulin pathway involved in aging, notable exceptions being IST-1, SKN-1, and HSB-1.

As shown by density plots (Figure 3A, left panel), nearly all proteins in the insulin-signaling gene set showed a consistent, albeit modest, increase in abundance between days 1 and 10 of adulthood when compared to the total quantified proteome, suggesting the hypothesis that the activity of this pathway late in life accelerates aging. The exceptions were the longevity-promoting transcription factor DAF-16 (FOXO3 in humans) and the serine/threonine protein kinase SGK-1 (SGK1), which decreased in abundance during aging. Since DAF-16 is negatively regulated by insulin/IGF-1 signaling, its downregulation makes sense; however, SGK-1, which was identified with at least ten razor/unique peptides in each of the three biological replicates (~31% sequence coverage), seems to be a true biological exception. The increase in abundance of the proteins SIR-2.1 (SIRT1) and DAF-18 (PTEN) was more striking than other components of the insulin-signaling gene set. These appear to be biological exceptions, as both proteins are positive regulators of longevity (Kaeberlein et al., 1999; Masse et al., 2005; Schmeisser et al., 2013). At least 9 razor/unique peptides from SIR-2.1 were detected in each biological replicate, and the sequence coverage was ~29%, while >26 razor/unique peptides from DAF-18 were detected with sequence coverage of ~49%. A similar trend was observed when the mTOR gene set we compiled was examined, consistent with the literature (Chen et al., 2009; Perkey et al., 2013), with an increase in TORC1 and TORC2 components observed between days 1 and 10 of adulthood (Figure 3A, right panel).

Having extracted information about known aging pathways from our dataset, we next performed GSEA for all pathways in the KEGG database using human homologs of the *C. elegans* proteins quantified in this study. Of the *C. elegans* protein-coding genes from our proteomic dataset, 4,394 had known human homologs (see the Supplemental Experimental Procedures), with multiple worm genes mapping to a single human homolog in many cases. Consequently, this list comprises 3,067 unique human homologs. To help exclude effects contributing to reproduction rather than organismal aging (Figure S1B), we focused on post-reproductive changes in protein abundance occurring between days 5 and 10 of adulthood. GSEA comparing changes in protein levels in day 5 versus day 10 animals confirmed an enrichment of pathways that also were enriched in the analysis of the subset of 627 proteins whose levels showed the largest change during aging (Figures 2B and 2C). As GSEA also takes into account the direction of the change in abundance, we observed that there is a marked downregulation of proteins involved in cellular metabolic pathways during aging. For example, enzymes involved in carbohydrate, amino acid, fatty acid, as well as drug and retinol metabolism are all decreased between days 5 and 10 of adulthood (Figures 3B and S5A).

GSEA Uncovers an Age-Dependent Decline in Peroxisomal Protein Import

In addition to age-dependent decreases in cellular metabolism, GSEA also showed a downregulation of proteins functioning in the KEGG pathway peroxisome (Figure 3B). A closer look revealed a decrease in abundance of ~30 proteins involved in peroxisomal protein import and function. For example, PEX3 (human ortholog)/PRX-3 (*C. elegans*), involved in the import of peroxisome membrane proteins, decreased in abundance during aging (Figure 4A), as did levels of PEX5/PRX-5, a protein known to be involved in the import of peroxisomal-matrix enzymes (Figures 4A and 4B; Petriv et al., 2002). Additionally, peroxisomal proteins involved in fatty acid oxidation, ether phospholipid biosynthesis, sterol precursor biosynthesis, amino acid metabolism, purine metabolism, and retinol metabolism decreased in abundance between days 5 and 10 of adulthood (Figure 4A).

The peroxisomal import protein PRX-5 has not been identified previously in proteomic studies on aging in wild-type nematodes,

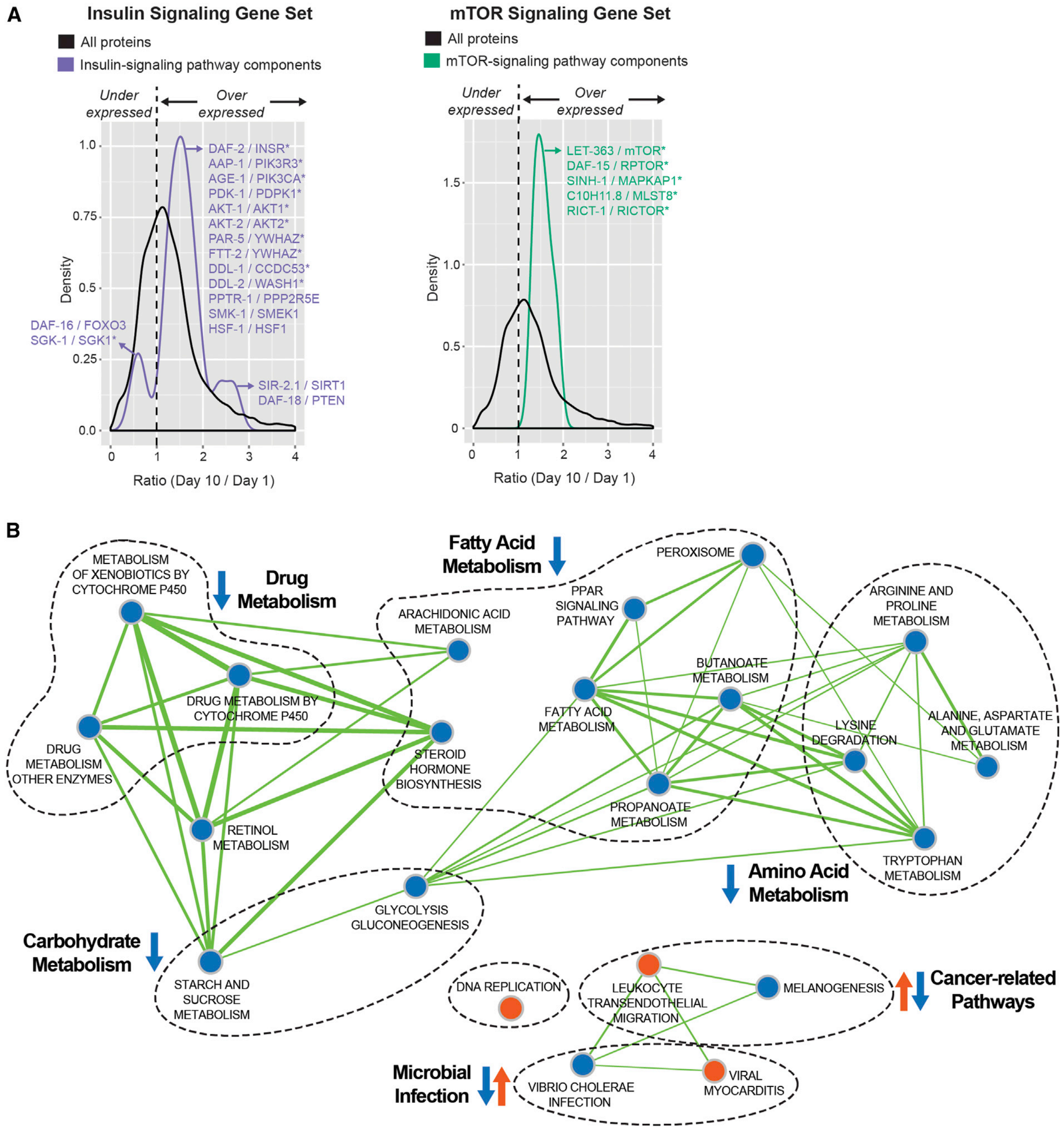

Figure 3. GSEA Uncovers a Downregulation of Cellular Metabolic Pathways with Age

(A) Density plots depicting the protein abundance of self-defined gene sets (left, insulin-IGF signaling; right, mTOR) relative to the entire dataset collected in this study. *C. elegans* proteins and their human orthologs are indicated in the figure. Negative regulators, in the context of extended longevity, are annotated with an asterisk (*).

(B) GSEA (KEGG pathways) output of the day 10 versus day 5 changes in protein abundance depicted schematically using Enrichment Map (Cytoscape). The thickness of the lines connecting nodes (i.e., KEGG pathways) indicates the overlap of quantified proteins common to both nodes.

See also Figure S5.

probably because of a limited depth of proteome coverage. When we mined existing microarray data of *C. elegans* aging (Youngman et al., 2011; Budovskaya et al., 2008), we found that *prx-5* mRNA levels decreased during aging in two independent studies using wild-type nematodes as well as temperature-sensitive sterile mutants (Figure 4C, left and right panels, respectively). Interestingly, analysis of mRNA transcript levels from published data (Youngman et al., 2011) for all the peroxisome proteins identified in our proteomic study revealed that, unlike *prx-5* transcript levels, the majority of mRNAs encoding peroxisome proteins were present at similar levels to the overall mRNA population, i.e., the entire dataset (Figure S5B, upper

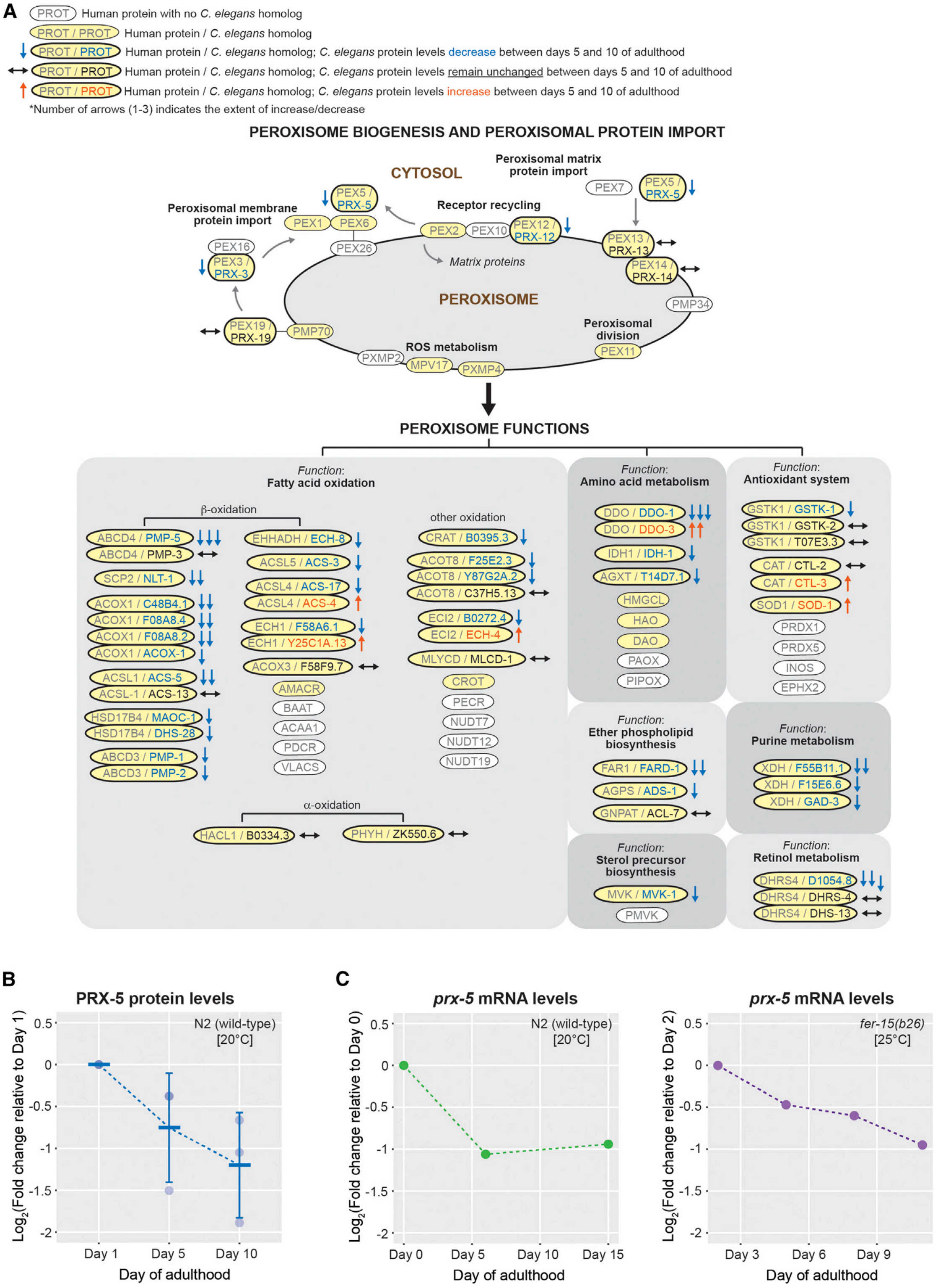

Figure 4. Levels of the Peroxisome Import Protein PRX-5 Decrease during Aging

(A) Schematic depicting an adaptation of the KEGG pathway peroxisome in humans (adapted from www.genome.jp/kegg/). *C. elegans* homologs of the human proteins are shaded yellow. Also indicated are the proteins quantified in this dataset, with orange, blue, or black arrows to denote whether the proteins were found to increase or decrease in abundance or to remain relatively unchanged between days 5 and 10 of adulthood, as measured in our study.

(legend continued on next page)

panel). This is in contrast with peroxisome protein levels measured in our proteomic study, the majority of which decreased in abundance when compared with the overall protein population (Figure S5B, lower panel). Examination of global mRNA levels (not just for the subset of peroxisome transcripts; Youngman et al., 2011) and our protein data showed a poor correlation (r = 0.25 [Pearson]; Figure S5C), consistent with previous findings (Walther et al., 2015).

To validate our proteomic data and assess whether a gradual failure of the peroxisomal import machinery occurs during aging (Figure 5A), we made use of a *C. elegans* peroxisome reporter in which GFP is linked to the peroxisome-targeting sequence (PTS1; GFP-SKL) and expressed using an *hsp-16.2* heat shock promoter (Thieringer et al., 2003). We verified that GFP-SKL localized correctly to foci indicative of peroxisomes (Thieringer et al., 2003) in the presence of functional import machinery (Figure 5B, middle panel). As predicted, when animals were treated with *prx-5* RNAi during adulthood, GFP failed to localize to the peroxisome and instead showed a diffuse cytoplasmic distribution (Figure 5B, right panel; Thieringer et al., 2003). To assess whether the foci were artifacts due to heat stress, we tested and confirmed that GFP localization without the PTS1 target sequence in *Phsp-16.2*::*gfp* animals was diffuse and devoid of distinct foci (Figure S6A).

Next we asked whether GFP-SKL was similarly mislocalized to the cytoplasm (see Figure 5B) during aging, as predicted from our MS analysis. Day 5 adult worms showed a mixed distribution of GFP-SKL between peroxisomes, indicated by intense spots/foci, and the cytoplasm (diffuse signal), whereas young day 1 animals showed GFP-SKL localized predominantly in peroxisomes (Figure 5C). Quantification of the cytoplasmic GFP signal confirmed an increase by day 5 relative to day 1 (Figure 5C, top left graph). However, as total GFP levels produced using the *hsp-16.2* promoter increased by day 5 (Figures S6B and S6C), we normalized the cytoplasmic GFP signal to total GFP intensity. After normalization, cytoplasmic GFP levels were still found to be significantly higher in day 5 adults compared with day 1 adults (Figure 5C, top right graph). Time points beyond day 5 were not considered, as GFP-SKL expression driven by the *hsp-16.2* promoter was markedly reduced by day 10 (Figures S6B and S6C).

Interestingly, the number of resolved foci (peroxisomes) increased significantly between days 1 and 5 of adulthood (Figures 5C, lower graph, and S6D). A similar observation has been made in senescent human fibroblasts (Legakis et al., 2002), where the authors speculate that this could be due to an age-dependent impairment in the signals that lead to peroxisome growth and division or, alternately, a failure to remove these structures by autophagy. The cytoplasmic mislocalization of GFP-SKL in aging animals was verified using a second strain, in which expression was controlled by the intestine-specific *ges-1* promoter (Figure 5D). Thus, we conclude that post-reproductive *C. elegans* hermaphrodites mislocalize proteins that are normally targeted to the peroxisome in young adults, likely due to a dysfunction of the peroxisome import machinery.

Reducing Insulin/IGF-1 Signaling Slows the Rate of Biological Aging without Slowing the Rate of Change of Much of the Age-Dependent Proteome

Long-lived insulin/IGF-1-pathway mutants exhibit reduced rates of tissue and behavioral aging. This raises the question of whether proteins that change in abundance with age in wild-type animals (Figure 2) show the expected slower rate of change when lifespan is extended. To test this, we first confirmed, in vivo, the age-dependent change in levels of several proteins whose abundance increased with age in our MS analysis, using GFP::protein-fusion worm strains (Figures 6A and S7A–S7G). Specifically, levels of GFP::fusions to either the DNA-binding protein HMG-11, the linker histone HIS-24, the histone H3 variant HIS-71, a nematode-specific protein (F55B11.4), or the p62/sequestosome homolog SQST-1, an indicator of autophagic flux, all increased during the course of aging in vivo (Figure 6A).

Next we asked how these proteins behaved in lifespan mutants. When HMG-11::GFP worms were raised under life-extending conditions (*daf-2* RNAi), HMG-11::GFP protein levels were significantly lower (younger pattern) on day 10 of adulthood, relative to control. Conversely, under conditions that accelerate aging and shorten lifespan (*hsf-1* RNAi) (Garigan et al., 2002), HMG-11::GFP protein levels were higher (older pattern) on day 5 relative to control. Thus, HMG-11 protein accumulation scales with the overall apparent rate of aging in these cases (Figure 6B). However, surprisingly, this was not the case for any of the remaining proteins. SQST-1::GFP levels were significantly lower in *daf-2*(*RNAi*) animals, as predicted, but they were not higher in *hsf-1*(*RNAi*) animals (Figure 6C). HIS-24::GFP, HIS-71::GFP, and F55B11.4::GFP protein levels were not lowered in *daf-2*(*RNAi*) worms; in fact, the levels of F55B11.4::GFP were higher (older pattern) in *daf-2*(*RNAi*) worms when compared with wild-type (Figure S7H). In *hsf-1*(*RNAi*) animals, in some cases (day 10 for HIS-24 and day 5 for F55B11.4), protein levels were lower (younger pattern) than in wild-type animals (Figure S7H).

These unexpected results prompted us to extend this analysis to include other proteins from the list of 627 proteins whose abundance changed most strikingly during aging. Of these 627 proteins, we concentrated on the 88 that also were identified in a recent study that analyzed the proteomes of wild-type (N2 Bristol), long-lived *daf-2*(*e1370*) mutants, and short-lived progeric *hsf-1*(*sy441*) mutant worms (Walther et al., 2015). Of the 88 proteins, 55 increased with age in wild-type animals in both the Walther et al. (2015) study and in our study, and 33 decreased with age. A closer look at the 55 proteins that increased with age revealed that 35% (19 proteins) accumulated to a lesser extent (more youthful pattern) in day 12 *daf-2*(*e1370*) adults relative to wild-type animals (Figure 6D). These included peptidases

(B) Graph shows the decrease in levels of the peroxisomal import protein PRX-5 during aging, at the indicated time points.
(C) Graph shows the decrease in mRNA levels of *prx-5* during aging observed in previously published datasets in wild-type nematodes (left; Youngman et al., 2011) and temperature-sensitive sterile mutants (right; Budovskaya et al., 2008).
See also Figure S5.

OPEN ACCESS
Cell Press

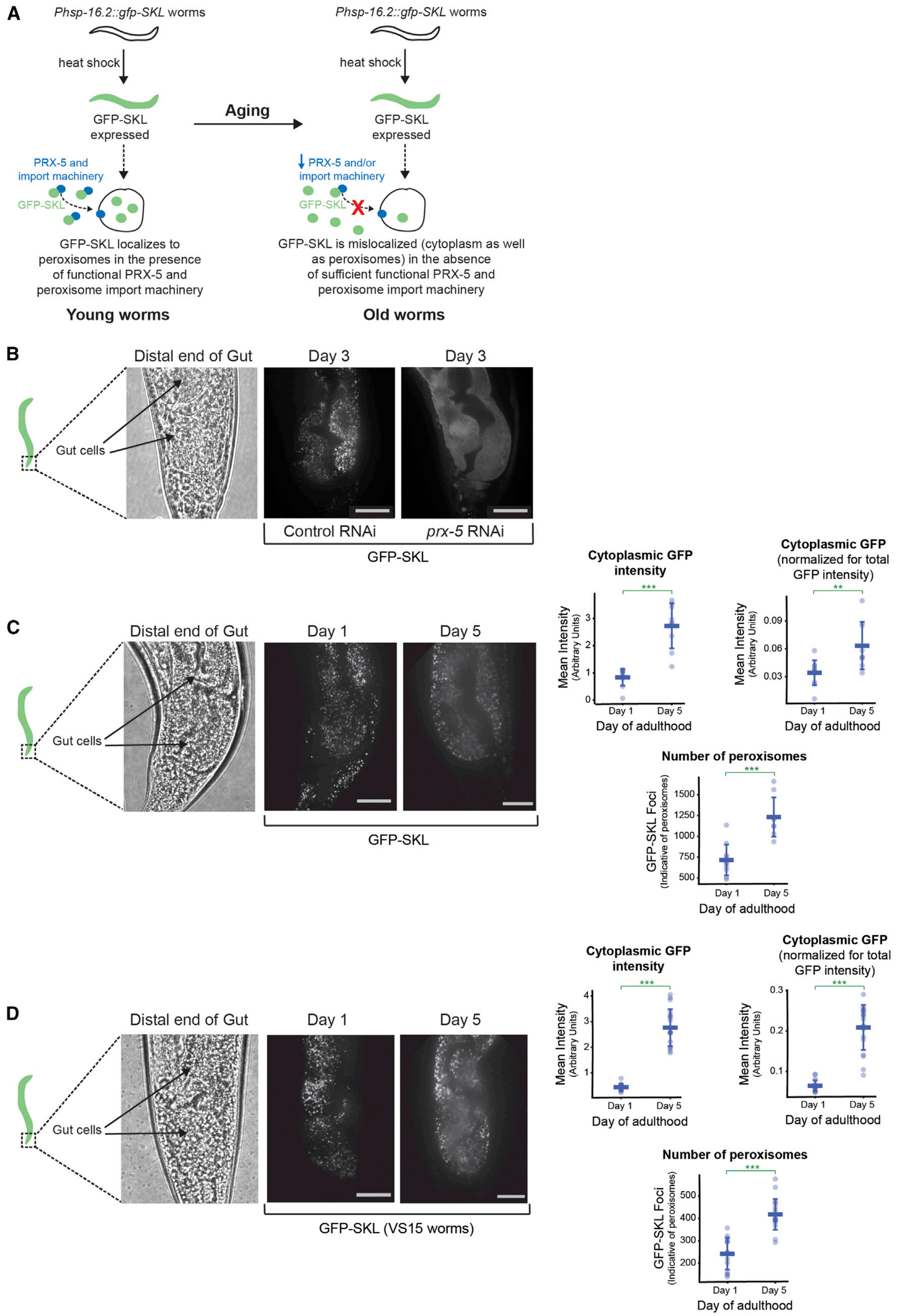

(legend on next page)

(F48E3.4 and T28H10.3), fatty acid-binding proteins (FAR-6 and LBP-2), the tumor necrosis factor-induced protein homolog T05E12.3, the carbohydrate-binding C-type lectin CLEC-146, transthyretin-related proteins (TTR-51, TTR-6, and TTR-30), the galectin LEC-2, which is believed to play a role in programmed cell death, the UDP glycosyltransferase UGT-31, and some nematode-specific proteins of unknown function.

Interestingly, a large proportion of the 55 age-increasing proteins (40%) did not show an appreciable change in levels between age-matched N2 and *daf-2* mutant animals. Further, 25% (14/55) of them were found to increase to a much greater extent (older pattern) in *daf-2*(*e1370*) mutants than in wild-type worms. In principle, these 14 proteins could function to delay aging, consistent with the findings of our lab and other labs that *daf-2* mutants express cell-protective mRNA species at high levels even in young adults (Murphy et al., 2003). In support of this hypothesis, we found that this subset of proteins contained the small heat shock protein HSP-12.3. However, this subset also included the aurora-related kinase AIR-2, which could be associated with the longer period of egg laying observed in *daf-2*(*e1370*) mutants (Gems et al., 1998). Other proteins in this subset included cytoskeletal and cuticle components (TBB-6, CHT-1, and TTH-1), the fatty acid-binding protein FAR-3, the nucleotide-binding protein CATP-3, and the enzymes HEX-1 (hexosaminidase) and F13H8.11 (phospholipase B1).

When the levels of the 55 proteins that increase with age in wild-type animals were measured in *hsf-1*(*sy441*) adults at day 6 (*hsf-1* mutants are mostly dead by day 12; hence, proteomic measurements during this period are likely to be biased), it was found that 62% (34/55) scaled with biological age, accumulating to a greater extent in these animals when compared with day 6 wild-type control animals (Figure 6D). However, 36% (20/55) of the proteins were unchanged between *hsf-1*(*sy441*) and control animals, and one protein of unknown function, T12D8.5, accumulated to a lesser extent in *hsf-1*(*sy441*) animals.

Similarly, we examined the 33 proteins from the pool of 627 age-variant proteins that were found to decrease during aging in wild-type animals in our study as well as in the Walther et al. (2015) study. Of these, 36% (12/33) scaled with biological age, decreasing to a lesser extent (i.e., the protein was more abundant, younger pattern) in long-lived *daf-2*(*e1370*) worms compared to wild-type worms at day 12 of adulthood (Figure 6E); 39% of the proteins remained unchanged between the two populations, and 24% (8/33) were present at lower levels (older pattern) in *daf-2*(*e1370*) mutants when compared to wild-type controls. In *hsf-1*(*sy441*) mutants at day 6 of adulthood, the levels of 27% (9/33) scaled with biological age, i.e., they were present at lower levels in these animals compared to age-matched controls (Figure 6E). However, the vast majority (61%) were unchanged between wild-type and *hsf-1*(*sy441*) worms, and 12% (4/33) were present at higher levels (younger pattern) in *hsf-1*(*sy441*) animals. Thus, the aging rate of much of the age-variant proteome, at least in terms of protein abundance, is unchanged under conditions where the rate of biological aging is altered by manipulating insulin/IGF signaling.

DISCUSSION

In this study, we present an in-depth measurement of age-related changes in protein abundance in *C. elegans*, facilitated by deep MS-based quantitative proteomic analysis that provides a major increase—almost a doubling—in coverage of the adult *C. elegans* proteome. Specifically, we have reproducibly identified >9,300 proteins in each of three biological replicates (and >11,000 in at least one of the three experiments), and we have monitored how these vary in abundance at three time points. The depth of proteome coverage obtained, together with the high degree of reproducibility among biological replicates, allowed the definition of strict statistical thresholds for assessing the significance of age-related protein abundance changes, rather than relying simply on arbitrary fold-change cutoffs. Using these stringent thresholds, we identified, in addition to factors previously shown to vary in abundance during aging in *C. elegans* (Liang et al., 2014; Walther et al., 2015; Zimmerman et al., 2015; see Figures S8A–S8F for comparisons between our study and previous proteomic studies), examples of proteins whose abundance variation and function have not previously been linked to aging.

Our dataset provides a valuable resource that can be used to generate hypotheses that will help to further our understanding of aging, which can then be tested in vivo. We provide two examples of this. First, our deep proteome analysis unveiled a decrease in the abundance of ~30 peroxisomal proteins with age (Figure 4A). In particular, we observed a decrease in abundance of the peroxisomal import protein PRX-5 (PEX5 in humans; Figure 4B) during aging, and we were curious to examine

Figure 5. Peroxisomal Protein Import Is Impaired during Aging

(A) Schematic depicting the correct peroxisomal localization of GFP targeted to the peroxisome (GFP-SKL) in young animals (left panel). If, as predicted by our MS analysis, the peroxisome import machinery is compromised during aging, GFP-SKL import into the peroxisome will be impaired (right panel).

(B–D) Scale bar, 10 μm. (B) GFP-SKL is correctly localized to the peroxisome in the intestine of control animals, but mislocalized to the cytosol under conditions where *prx-5* is knocked down with RNAi (n = 7). (C) GFP-SKL begins to accumulate in the cytoplasm of aging animals (day 5 adults) when compared to day 1 adults. Shown are representative images from the distal intestinal cells. Cytoplasmic (diffuse) GFP intensity was quantified, summed, and plotted from 81 individual z-sections at 0.5-μm intervals for each worm to minimize masking of the cytoplasmic signal by intense GFP foci (peroxisomes) as well as to minimize bleed through. The cytoplasmic signal was found to increase significantly with age (upper left graph; p value calculated using an unpaired t test = 7.68×10^{-5}; n = 11 individuals for day 1 adults and n = 9 for day 5 animals). As the total *hsp-16.2*-driven GFP-SKL signal also was found to increase with age, cytoplasmic GFP measurements were corrected for total intensity in each image and age-dependent changes remained significant (upper right graph; p value from unpaired t test = 0.009). Also indicated is a count of the number of dots resolved (peroxisomes) at days 1 and 5 from a single z-section. This was found to increase significantly by day 5 (lower graph; p value from unpaired t test = 7.86×10^{-5}). (D) As above, but we used worms that express GFP-SKL under the intestine-specific *ges-1* promoter (strain VS15). Cytoplasmic GFP-SKL intensity increased significantly with age (p value from unpaired t test = 1.56×10^{-11} for raw cytosolic intensities [left graph] and 4.54×10^{-10} for cytosolic GFP intensity normalized to total GFP intensity [right graph]; n = 15 for day 1 adults and n = 19 for day 5 adults). As in (C), the number of dots resolved also increased significantly with age (lower graph; p value from unpaired t test = 4.46×10^{-8}).

See also Figure S6.

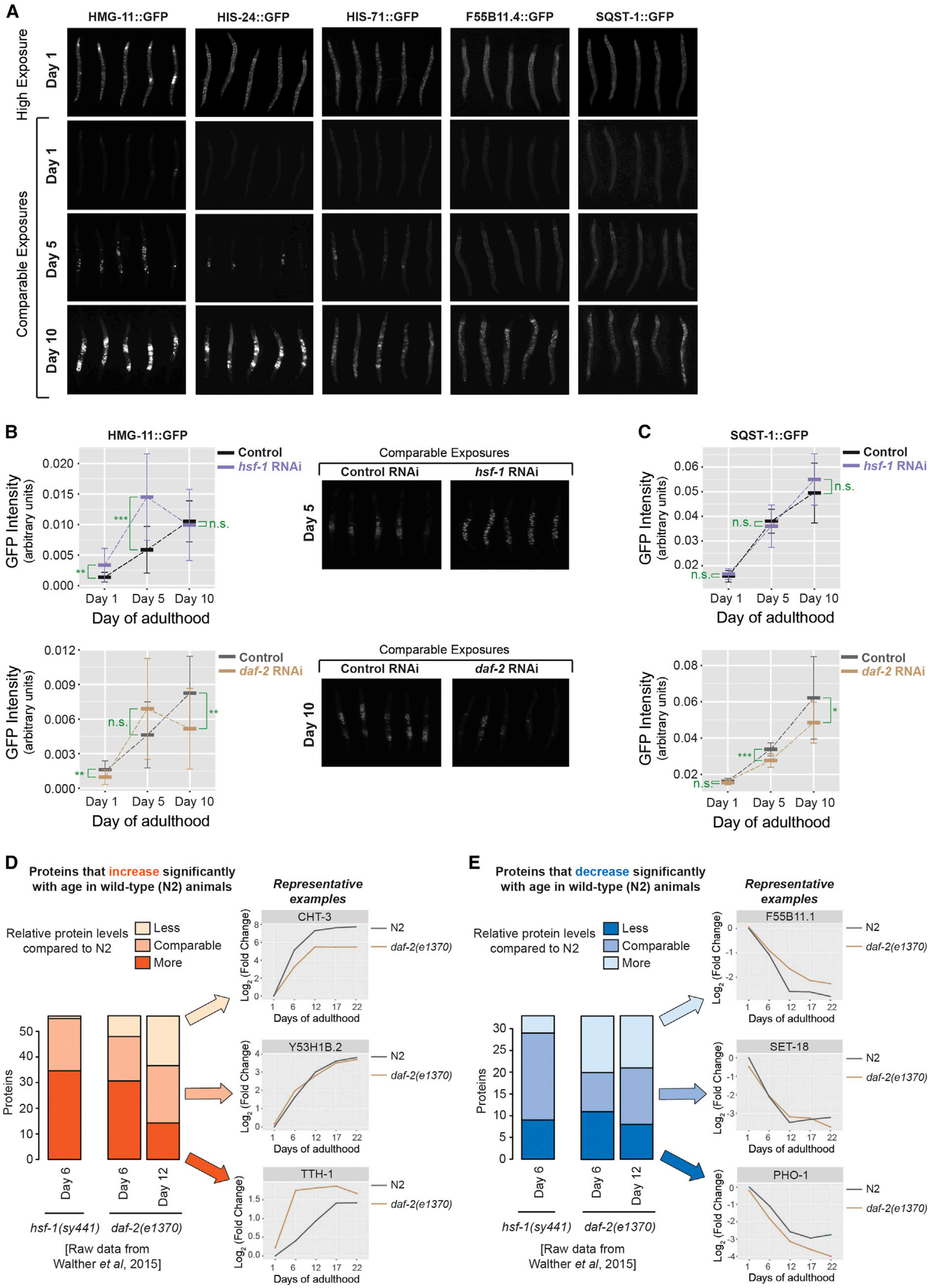

(legend on next page)

whether this results in impaired peroxisomal protein import during aging. There are reports of an age-dependent mislocalization of catalase to the cytoplasm, and studies in middle- and late-passage human fibroblasts speculate that catalase mislocalization during replicative senescence may be attributed to impaired peroxisome import (Legakis et al., 2002). Whether this phenomenon also is observed during organismal aging and applies to all PTS1-containing peroxisomal proteins or only to catalase, which has a non-canonical PTS1 (Lys-Ala-Asn-Leu rather than Ser-Lys-Leu; Purdue et al., 1996), was still unclear. In this study, we used a peroxisome-targeted GFP-PTS1 reporter and showed, in vivo, that this protein was mislocalized during aging in two independent worm strains (Figures 5C and 5D). Thus, our data support a model wherein impaired peroxisome protein import caused by decreased PRX-5 levels leads to peroxisome dysfunction with age.

Like mitochondria, peroxisomes generate reactive oxygen species (ROS), and, although mitochondria are responsible for the majority of the ROS produced within a cell, ROS generated in peroxisomes appear to have a profound impact on mitochondria. Peroxisomal catalase deficiency disrupts mitochondrial redox balance (Hwang et al., 2012), and the induction of ROS production in the peroxisome results in increased mitochondrial fragmentation (a hallmark of aging) and an altered redox potential (Ivashchenko et al., 2011). Peroxisomes and mitochondria therefore appear to be intimately linked, and the interplay between the two organelles could be important in the context of aging. Although *prx-5* is essential for nematode larval development (Thieringer et al., 2003), surprisingly, when knocked down in adult nematodes, lifespan was not shortened but, if anything, increased slightly (Zhou et al., 2012; Curran and Ruvkun, 2007). In our hands, adult-only *prx-5* RNAi treatment resulted in a significant decrease in brood size (p = 0.00019; Figure S6E), raising the possibility that lifespan is increased in nematodes via the pathway that extends lifespan in response to germline depletion or potentially by a cell-protective response triggered by, for example, an altered redox potential. Interestingly, in yeast, although *Δpex5* cells show minimal peroxisome biogenesis defects, they have a dramatically shorter chronological lifespan when grown on 0.5% glucose (mean lifespan ~7 versus ~16 days in wild-type cells and maximum lifespan ~10 versus ~23 days), underscoring the importance of correctly localized peroxisome matrix proteins imported via functional Pex5p (PEX5/PRX-5) in maintaining normal lifespan (Lefevre et al., 2013).

A large compendium of proteins whose levels change with age is a powerful tool to better understand how single-gene mutations, such as *daf-2* insulin/IGF-1-receptor mutations, can slow aging and extend lifespan. *daf-2* mutants are known to express many cell-protective proteins at elevated levels, even as young adults (e.g., proteasome subunits and molecular chaperones [Depuydt et al., 2013; Walther et al., 2015; Walker et al., 2001] as well as some autophagy components [Hashimoto et al., 2009; Hansen et al., 2008]). To test whether abundances of age-variant proteins scale with the rate of biological aging, we examined the levels of some of the age-variant proteins identified in our study in vivo in *daf-2*(*RNAi*) worms. As expected, the accumulation rates of some proteins were slower in *daf-2*(*RNAi*) animals than in wild-type. However, surprisingly, this was not the case for all the proteins we examined. When this analysis was expanded to include proteomic data from other studies (Walther et al., 2015), we confirmed that only a minor fraction of the age-variant proteins identified in wild-type animals showed reduced rates of change in long-lived *daf-2* mutants. The majority of proteins accumulated at similar rates in chronologically age-matched wild-type animals versus *daf-2*(*e1370*) mutants. Stated simply, these proteins didn't seem to know that they were in a long-lived mutant.

These results agree with our lab's previous study of mRNA changes during early adulthood (days 1–6) in *daf-2*(−) animals (Murphy et al., 2003), where again, the age-specific abundances of many mRNAs changed at a wild-type rate. However, the significance of this was not clear because these mRNAs might have been linked to reproduction, which takes place during this period, involves two-thirds of the animal's cells, and was known to be fairly normal in these mutants. Our finding that this observation also holds true for protein levels, and extends well beyond the reproductive phase of adulthood, is thus significant. It suggests that the aging rate of much of the age-variant proteome, at least in terms of protein abundance, is unchanged in these long-lived animals. More fundamentally, it implies that an important biological clock still ticks at a normal rate in these animals, in spite of the change in their rates of morphological decline. These mutants probably age more slowly at the organismal level and live long because the relatively small set of cell-protective proteins and proteostasis

Figure 6. Most Age-Variant Proteins Do Not Scale with Biological Age in Long-Lived Insulin/IGF-1 Mutants

(A) Snapshots of GFP fluorescence at days 1, 5, and 10 of adulthood for the indicated GFP::protein fusions. Shown are high exposures at day 1 (upper panels) and comparable exposures at days 1, 5, and 10 (lower panels) (n = 20 per experiment).

(B) HMG-11::GFP-expressing worms were grown on the indicated RNAi-bacteria (initiated at L1 stage) and GFP fluorescence was quantified on days 1, 5, and 10 of adulthood. Graphs show mean ± SD (n = 20 worms). Significance was calculated using an unpaired t test (*p < 0.05, **p < 0.01, ***p < 0.001; n.s., not significant). The *daf-2* and *hsf-1* dsRNA constructs are in different vector backbones, hence the corresponding empty vector control for each was used. Snapshots of images taken at day 5 (*hsf-1* RNAi) and day 10 (*daf-2* RNAi) also are shown.

(C) As in (B), except we used SQST-1::GFP-expressing hermaphrodites (n = 20 except for day 10 *hsf-1* RNAi where n = 11).

(D and E) 88 proteins from the 627 age-variant proteins identified in this study were detected in a previous study by Walther et al. (2015) in wild-type, *daf-2*(*e1370*), and *hsf-1*(*sy441*) animals, and they were found to show similar abundance trends in wild-type animals when compared to our dataset. Of these, those that increased with age in wild-type animals also were quantified in *daf-2*(*e1370*) and *hsf-1*(*sy441*) worms (D). The stacked bar plot shows how the levels of these proteins vary in the different strains relative to wild-type animals at day 6 (*hsf-1*) or days 6 and 12 (*daf-2*) of adulthood, using raw data from Walther et al. (2015). Also indicated are representative examples of proteins present at lower, comparable, or higher levels in day 12 *daf-2* mutants compared to wild-type controls. (E) As in (D), except that proteins whose levels were found to decrease in wild-type animals were analyzed. As in (D), raw data from Walther et al. (2015) were used to generate the bar plots.

See also Figure S7.

regulators, known to be expressed constitutively at high (or low) levels even in young *daf-2* adults (Murphy et al., 2003; Dong et al., 2007), alter the extent to which normal time-dependent processes affect biological aging.

In summary, here we describe a highly reproducible proteomic dataset containing >11,000 proteins identified in the adult worm (>9,300 reproducibly in three biological replicates), and we show how these proteins change during aging. Network analysis of our data, together with follow-up in vivo studies to test hypotheses generated using our dataset, has identified impairments in peroxisomal protein import during aging and, surprisingly, the finding that the majority of age-variant proteins do not scale with the rate of biological aging in long-lived insulin/IGF-1-receptor mutants. Additionally, this deep proteomic analysis contributes candidate determinants for biological aging (organismal and tissue decline) and a large set of proteins whose abundances, to our knowledge, were not previously shown to change with age, including proteins that may serve as biomarkers of aging and can be tested genetically for a causal role. To maximize the value of this resource to the community, all of these data are provided in a convenient, searchable online database (https://www.peptracker.com/epd).

EXPERIMENTAL PROCEDURES

Detailed procedures for SILAC labeling, sub-cellular fractionation, LC-MS/MS and data analysis, microscopy, RNAi treatment, and in vivo assays are included in the Supplemental Experimental Procedures.

C. elegans Strains and Maintenance

The N2 (Bristol) strain was used as wild-type. HZ859 was a gift from Hong Zhang and Malene Hansen, GFP-SKL was from Monica Driscoll, and TH184 and TH237 were from Mihail Sarov. All other strains were from the Caenorhabditis Genetics Center (CGC). The strains were grown and maintained at 20°C as previously described (Brenner, 1974) with sufficient food (*E. coli* OP50 unless otherwise indicated) for at least three generations prior to use.

Data Sharing

The data have been assembled into a searchable, online resource with a user-friendly graphical interface, maintained by the A.I.L. lab, to provide convenient and open access to the community (https://www.peptracker.com/epd/). Our data will also shortly be incorporated into WormBase, a resource that is used extensively by the worm community.

ACCESSION NUMBERS

The accession numbers for all of the raw MS data, as well as MaxQuant output files (containing protein identification/matching and quantification data), reported in this paper are ProteomeXchange Consortium (http://proteomecentral.proteomexchange.org): PXD004584 and MassIVE (http://massive.ucsd.edu/ProteoSAFe/): MSV000079263.

SUPPLEMENTAL INFORMATION

Supplemental Information includes Supplemental Experimental Procedures, eight figures, and four tables and can be found with this article online at http://dx.doi.org/10.1016/j.cels.2016.06.011.

AUTHOR CONTRIBUTIONS

V.N. conceived and performed the experiments, with input from T.L., A.G., A.I.L., and C.K. V.N., A.I.L., A.G., and C.K. secured funding. V.N. and T.L. wrote R scripts for data analysis. E.P. and V.N. optimized SILAC protocols for worms. A.B.M. integrated the proteomic data into the EPD (https://www.peptracker.com/epd/). V.N., C.K., A.I.L., T.L., and A.G. wrote the manuscript.

ACKNOWLEDGMENTS

We would like to thank members of the C.K., A.G., and A.I.L. labs and WormBase (Gary Williams, Kevin Howe, and Chris Grove) for helpful discussion, advice, and experimental protocols. We are grateful to Monica Driscoll, Malene Hansen, and Mihail Sarov for strains. Some strains were provided by the CGC, which is funded by NIH Office of Research Infrastructure Programs (P40 OD010440). This research was funded by a Sir Henry Wellcome Postdoctoral Fellowship to V.N. (098646/Z/12/Z), a Wellcome Trust Strategic Award to A.I.L. (073980/Z/03/BR), a Wellcome Trust Senior Fellowship to A.G. (0909444/Z/09/Z), an NIH R01 Grant to C.K. (R01 AG11816), and by Calico Life Sciences LLC, where V.N. and C.K. are now employed.

Received: January 22, 2016
Revised: May 11, 2016
Accepted: June 21, 2016
Published: July 21, 2016

REFERENCES

Brenner, S. (1974). The genetics of Caenorhabditis elegans. Genetics *77*, 71–94.

Budovskaya, Y.V., Wu, K., Southworth, L.K., Jiang, M., Tedesco, P., Johnson, T.E., and Kim, S.K. (2008). An elt-3/elt-5/elt-6 GATA transcription circuit guides aging in C. elegans. Cell *134*, 291–303.

Chen, C., Liu, Y., Liu, Y., and Zheng, P. (2009). mTOR regulation and therapeutic rejuvenation of aging hematopoietic stem cells. Sci. Signal. *2*, ra75.

Copes, N., Edwards, C., Chaput, D., Saifee, M., Barjuca, I., Nelson, D., Paraggio, A., Saad, P., Lipps, D., Stevens, S.M., Jr., and Bradshaw, P.C. (2015). Metabolome and proteome changes with aging in Caenorhabditis elegans. Exp. Gerontol. *72*, 67–84.

Cox, J., and Mann, M. (2008). MaxQuant enables high peptide identification rates, individualized p.p.b.-range mass accuracies and proteome-wide protein quantification. Nat. Biotechnol. *26*, 1367–1372.

Croll, N.A., Smith, J.M., and Zuckerman, B.M. (1977). The aging process of the nematode Caenorhabditis elegans in bacterial and axenic culture. Exp. Aging Res. *3*, 175–189.

Curran, S.P., and Ruvkun, G. (2007). Lifespan regulation by evolutionarily conserved genes essential for viability. PLoS Genet. *3*, e56.

David, D.C., Ollikainen, N., Trinidad, J.C., Cary, M.P., Burlingame, A.L., and Kenyon, C. (2010). Widespread protein aggregation as an inherent part of aging in C. elegans. PLoS Biol. *8*, e1000450.

Depuydt, G., Xie, F., Petyuk, V.A., Shanmugam, N., Smolders, A., Dhondt, I., Brewer, H.M., Camp, D.G., 2nd, Smith, R.D., and Braeckman, B.P. (2013). Reduced insulin/insulin-like growth factor-1 signaling and dietary restriction inhibit translation but preserve muscle mass in Caenorhabditis elegans. Mol. Cell. Proteomics *12*, 3624–3639.

Dong, M.-Q., Venable, J.D., Au, N., Xu, T., Park, S.K., Cociorva, D., Johnson, J.R., Dillin, A., and Yates, J.R., 3rd (2007). Quantitative mass spectrometry identifies insulin signaling targets in C. elegans. Science *317*, 660–663.

Fredens, J., Engholm-Keller, K., Giessing, A., Pultz, D., Larsen, M.R., Højrup, P., Møller-Jensen, J., and Færgeman, N.J. (2011). Quantitative proteomics by amino acid labeling in C. elegans. Nat. Methods *8*, 845–847.

Garigan, D., Hsu, A.L., Fraser, A.G., Kamath, R.S., Ahringer, J., and Kenyon, C. (2002). Genetic analysis of tissue aging in Caenorhabditis elegans: a role for heat-shock factor and bacterial proliferation. Genetics *161*, 1101–1112.

Gems, D., Sutton, A.J., Sundermeyer, M.L., Albert, P.S., King, K.V., Edgley, M.L., Larsen, P.L., and Riddle, D.L. (1998). Two pleiotropic classes of daf-2

mutation affect larval arrest, adult behavior, reproduction and longevity in Caenorhabditis elegans. Genetics *150*, 129–155.

Grün, D., Kirchner, M., Thierfelder, N., Stoeckius, M., Selbach, M., and Rajewsky, N. (2014). Conservation of mRNA and protein expression during development of C. elegans. Cell Rep. *6*, 565–577.

Hansen, M., Chandra, A., Mitic, L.L., Onken, B., Driscoll, M., and Kenyon, C. (2008). A role for autophagy in the extension of lifespan by dietary restriction in C. elegans. PLoS Genet. *4*, e24.

Hashimoto, Y., Ookuma, S., and Nishida, E. (2009). Lifespan extension by suppression of autophagy genes in Caenorhabditis elegans. Genes Cells *14*, 717–726.

Herndon, L.A., Schmeissner, P.J., Dudaronek, J.M., Brown, P.A., Listner, K.M., Sakano, Y., Paupard, M.C., Hall, D.H., and Driscoll, M. (2002). Stochastic and genetic factors influence tissue-specific decline in ageing C. elegans. Nature *419*, 808–814.

Huang, C., Xiong, C., and Kornfeld, K. (2004). Measurements of age-related changes of physiological processes that predict lifespan of Caenorhabditis elegans. Proc. Natl. Acad. Sci. USA *101*, 8084–8089.

Huang, W., Sherman, B.T., and Lempicki, R.A. (2009). Systematic and integrative analysis of large gene lists using DAVID bioinformatics resources. Nat. Protoc. *4*, 44–57.

Hwang, I., Lee, J., Huh, J.Y., Park, J., Lee, H.B., Ho, Y.S., and Ha, H. (2012). Catalase deficiency accelerates diabetic renal injury through peroxisomal dysfunction. Diabetes *61*, 728–738.

Ivashchenko, O., Van Veldhoven, P.P., Brees, C., Ho, Y.S., Terlecky, S.R., and Fransen, M. (2011). Intraperoxisomal redox balance in mammalian cells: oxidative stress and interorganellar cross-talk. Mol. Biol. Cell *22*, 1440–1451.

Jiang, H.-C., Hsu, J.M., Yen, C.P., Chao, C.C., Chen, R.H., and Pan, C.L. (2015). Neural activity and CaMKII protect mitochondria from fragmentation in aging Caenorhabditis elegans neurons. Proc. Natl. Acad. Sci. USA *112*, 8768–8773.

Kaeberlein, M., McVey, M., and Guarente, L. (1999). The SIR2/3/4 complex and SIR2 alone promote longevity in Saccharomyces cerevisiae by two different mechanisms. Genes Dev. *13*, 2570–2580.

Kanehisa, M., and Goto, S. (2000). KEGG: kyoto encyclopedia of genes and genomes. Nucleic Acids Res. *28*, 27–30.

Kanehisa, M., Goto, S., Sato, Y., Kawashima, M., Furumichi, M., and Tanabe, M. (2014). Data, information, knowledge and principle: back to metabolism in KEGG. Nucleic Acids Res. *42*, D199–D205.

Kapahi, P., Chen, D., Rogers, A.N., Katewa, S.D., Li, P.W., Thomas, E.L., and Kockel, L. (2010). With TOR, less is more: a key role for the conserved nutrient-sensing TOR pathway in aging. Cell Metab. *11*, 453–465.

Kenyon, C.J. (2010). The genetics of ageing. Nature *464*, 504–512.

Kenyon, C., Chang, J., Gensch, E., Rudner, A., and Tabtiang, R. (1993). A C. elegans mutant that lives twice as long as wild type. Nature *366*, 461–464.

Kimura, K.D., Tissenbaum, H.A., Liu, Y., and Ruvkun, G. (1997). daf-2, an insulin receptor-like gene that regulates longevity and diapause in Caenorhabditis elegans. Science *277*, 942–946.

Lapierre, L.R., and Hansen, M. (2012). Lessons from C. elegans: signaling pathways for longevity. Trends Endocrinol. Metab. *23*, 637–644.

Larance, M., Bailly, A.P., Pourkarimi, E., Hay, R.T., Buchanan, G., Coulthurst, S., Xirodimas, D.P., Gartner, A., and Lamond, A.I. (2011). Stable-isotope labeling with amino acids in nematodes. Nat. Methods *8*, 849–851.

Lefevre, S.D., van Roermund, C.W., Wanders, R.J., Veenhuis, M., and van der Klei, I.J. (2013). The significance of peroxisome function in chronological aging of Saccharomyces cerevisiae. Aging Cell *12*, 784–793.

Legakis, J.E., Koepke, J.I., Jedeszko, C., Barlaskar, F., Terlecky, L.J., Edwards, H.J., Walton, P.A., and Terlecky, S.R. (2002). Peroxisome senescence in human fibroblasts. Mol. Biol. Cell *13*, 4243–4255.

Liang, V., Ullrich, M., Lam, H., Chew, Y.L., Banister, S., Song, X., Zaw, T., Kassiou, M., Götz, J., and Nicholas, H.R. (2014). Altered proteostasis in aging and heat shock response in C. elegans revealed by analysis of the global and de novo synthesized proteome. Cell. Mol. Life Sci. *71*, 3339–3361.

Ly, T., Ahmad, Y., Shlien, A., Soroka, D., Mills, A., Emanuele, M.J., Stratton, M.R., and Lamond, A.I. (2014). A proteomic chronology of gene expression through the cell cycle in human myeloid leukemia cells. eLife *3*, e01630.

Masse, I., Molin, L., Billaud, M., and Solari, F. (2005). Lifespan and dauer regulation by tissue-specific activities of Caenorhabditis elegans DAF-18. Dev. Biol. *286*, 91–101.

Morley, J.F., and Morimoto, R.I. (2004). Regulation of longevity in Caenorhabditis elegans by heat shock factor and molecular chaperones. Mol. Biol. Cell *15*, 657–664.

Murphy, C.T., and Hu, P.J. (2013). Insulin/insulin-like growth factor signaling in C. elegans. WormBook, 1–43.

Murphy, C.T., McCarroll, S.A., Bargmann, C.I., Fraser, A., Kamath, R.S., Ahringer, J., Li, H., and Kenyon, C. (2003). Genes that act downstream of DAF-16 to influence the lifespan of Caenorhabditis elegans. Nature *424*, 277–283.

Perkey, E., Fingar, D., Miller, R.A., and Garcia, G.G. (2013). Increased mammalian target of rapamycin complex 2 signaling promotes age-related decline in CD4 T cell signaling and function. J. Immunol. *191*, 4648–4655.

Petriv, O.I., Pilgrim, D.B., Rachubinski, R.A., and Titorenko, V.I. (2002). RNA interference of peroxisome-related genes in C. elegans: a new model for human peroxisomal disorders. Physiol. Genomics *10*, 79–91.

Pourkarimi, E., Greiss, S., and Gartner, A. (2012). Evidence that CED-9/Bcl2 and CED-4/Apaf-1 localization is not consistent with the current model for C. elegans apoptosis induction. Cell Death Differ. *19*, 406–415.

Purdue, P.E., Castro, S.M., Protopopov, V., and Lazarow, P.B. (1996). Targeting of human catalase to peroxisomes is dependent upon a novel C-terminal peroxisomal targeting sequence. Ann. N Y Acad. Sci. *804*, 775–776.

Schmeisser, K., Mansfeld, J., Kuhlow, D., Weimer, S., Priebe, S., Heiland, I., Birringer, M., Groth, M., Segref, A., Kanfi, Y., et al. (2013). Role of sirtuins in lifespan regulation is linked to methylation of nicotinamide. Nat. Chem. Biol. *9*, 693–700.

Schrimpf, S.P., Weiss, M., Reiter, L., Ahrens, C.H., Jovanovic, M., Malmström, J., Brunner, E., Mohanty, S., Lercher, M.J., Hunziker, P.E., et al. (2009). Comparative functional analysis of the Caenorhabditis elegans and Drosophila melanogaster proteomes. PLoS Biol. *7*, e48.

Subramanian, A., Tamayo, P., Mootha, V.K., Mukherjee, S., Ebert, B.L., Gillette, M.A., Paulovich, A., Pomeroy, S.L., Golub, T.R., Lander, E.S., and Mesirov, J.P. (2005). Gene set enrichment analysis: a knowledge-based approach for interpreting genome-wide expression profiles. Proc. Natl. Acad. Sci. USA *102*, 15545–15550.

Supek, F., Bošnjak, M., Škunca, N., and Šmuc, T. (2011). REVIGO summarizes and visualizes long lists of gene ontology terms. PloS One *6*, e21800.

Taylor, R.C., and Dillin, A. (2011). Aging as an event of proteostasis collapse. Cold Spring Harb. Perspect. Biol. *3*, a004440–a004440.

Thieringer, H., Moellers, B., Dodt, G., Kunau, W.H., and Driscoll, M. (2003). Modeling human peroxisome biogenesis disorders in the nematode Caenorhabditis elegans. J. Cell Sci. *116*, 1797–1804.

Timmons, L., and Fire, A. (1998). Specific interference by ingested dsRNA. Nature *395*, 854.

Vellai, T., Takacs-Vellai, K., Zhang, Y., Kovacs, A.L., Orosz, L., and Müller, F. (2003). Genetics: influence of TOR kinase on lifespan in C. elegans. Nature *426*, 620–620.

Walker, G.A., White, T.M., McColl, G., Jenkins, N.L., Babich, S., Candido, E.P., Johnson, T.E., and Lithgow, G.J. (2001). Heat shock protein accumulation is upregulated in a long-lived mutant of Caenorhabditis elegans. J. Gerontol. A Biol. Sci. Med. Sci. *56*, B281–B287.

Walther, D.M., Kasturi, P., Zheng, M., Pinkert, S., Vecchi, G., Ciryam, P., Morimoto, R.I., Dobson, C.M., Vendruscolo, M., Mann, M., and Hartl, F.U.

(2015). Widespread proteome remodeling and aggregation in aging C. elegans. Cell *161*, 919–932.

Ward, J.H., Jr. (2012). Hierarchical grouping to optimize an objective function. J. Am. Stat. Assoc. *58*, 236–244.

Youngman, M.J., Rogers, Z.N., and Kim, D.H. (2011). A decline in p38 MAPK signaling underlies immunosenescence in Caenorhabditis elegans. PLoS Genet. *7*, e1002082.

Zhou, B., Yang, L., Li, S., Huang, J., Chen, H., Hou, L., Wang, J., Green, C.D., Yan, Z., Huang, X., et al. (2012). Midlife gene expressions identify modulators of aging through dietary interventions. Proc. Natl. Acad. Sci. USA *109*, E1201–E1209.

Zimmerman, S.M., Hinkson, I.V., Elias, J.E., and Kim, S.K. (2015). Reproductive aging drives protein accumulation in the uterus and limits lifespan in C. elegans. PLoS Genet. *11*, e1005725.

Cell Systems, Volume *3*

Supplemental Information

Deep Proteome Analysis Identifies Age-Related Processes in *C. elegans*

Vikram Narayan, Tony Ly, Ehsan Pourkarimi, Alejandro Brenes Murillo, Anton Gartner, Angus I. Lamond, and Cynthia Kenyon

Figure S1

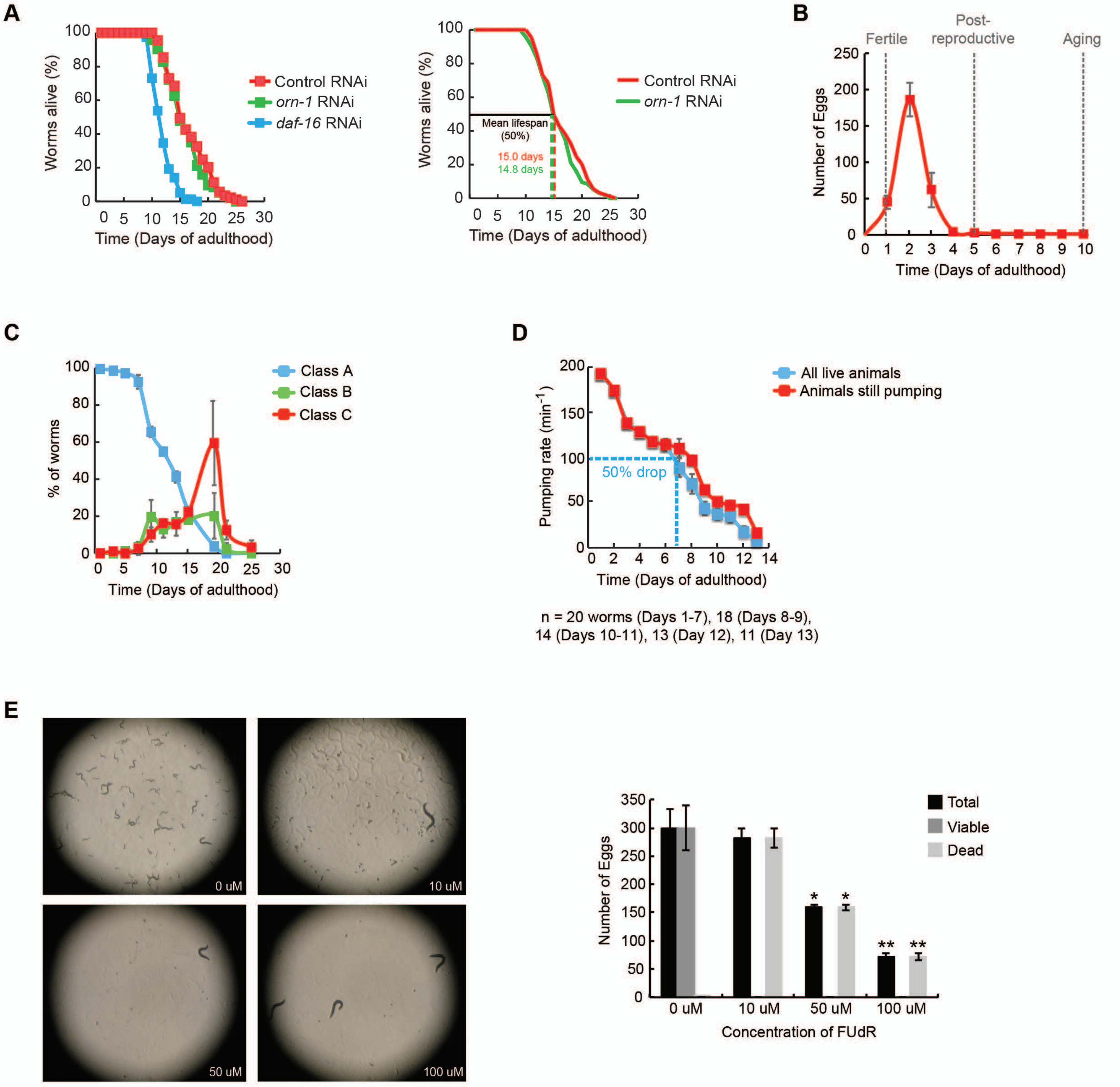

F

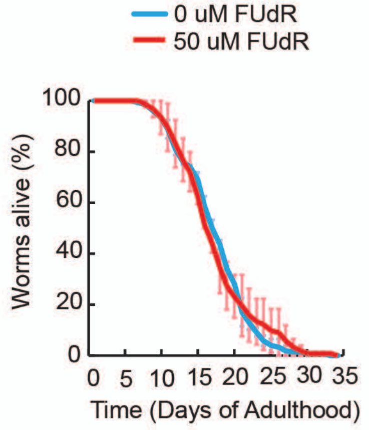

Background	Experiment	Treatment	#Total worms	#Censored	#Scored	Mean lifespan (days of adulthood)
N2	1	0 uM FUdR	100	21	79	16.4
		50 uM FUdR	100	6	94	16.2
N2	2	0 uM FUdR	100	25	75	17.2
		50 uM FUdR	100	24	76	16

Figure S2

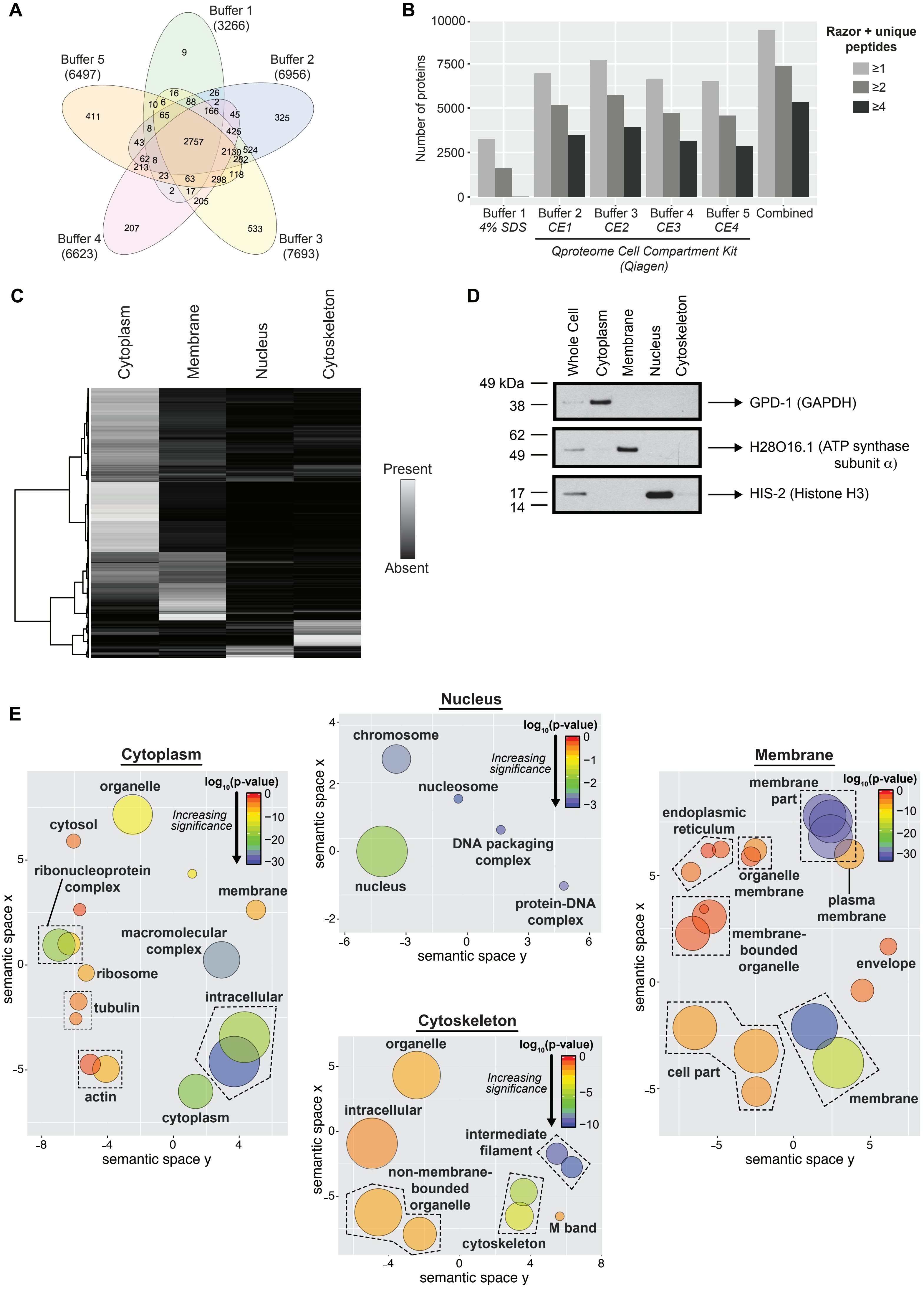
A
Buffer 1 (3266)
Buffer 2 (6956)
Buffer 3 (7693)
Buffer 4 (6623)
Buffer 5 (6497)
9
16
26
88
2
166
10
6
65
45
325
411
8
425
43
2757
2130
524
282
62
8
213
23
63
298
118
2
17
205
207
533
B
Razor + unique peptides
≥1
≥2
≥4
Number of proteins
10000
7500
5000
2500
0
Buffer 1 4% SDS
Buffer 2 CE1
Buffer 3 CE2
Buffer 4 CE3
Buffer 5 CE4
Combined
Qproteome Cell Compartment Kit (Qiagen)
C
Cytoplasm
Membrane
Nucleus
Cytoskeleton
Present
Absent
D
Whole Cell
Cytoplasm
Membrane
Nucleus
Cytoskeleton
49 kDa
38
62
49
17
14
GPD-1 (GAPDH)
H28O16.1 (ATP synthase subunit α)
HIS-2 (Histone H3)
E
Cytoplasm
organelle
cytosol
ribonucleoprotein complex
membrane
macromolecular complex
ribosome
tubulin
intracellular
actin
cytoplasm
log10(p-value)
Increasing significance
semantic space x
semantic space y
Nucleus
chromosome
nucleosome
DNA packaging complex
nucleus
protein-DNA complex
Cytoskeleton
organelle
intracellular
intermediate filament
non-membrane-bounded organelle
M band
cytoskeleton
Membrane
membrane part
endoplasmic reticulum
organelle membrane
plasma membrane
membrane-bounded organelle
envelope
cell part
membrane

Figure S2

F

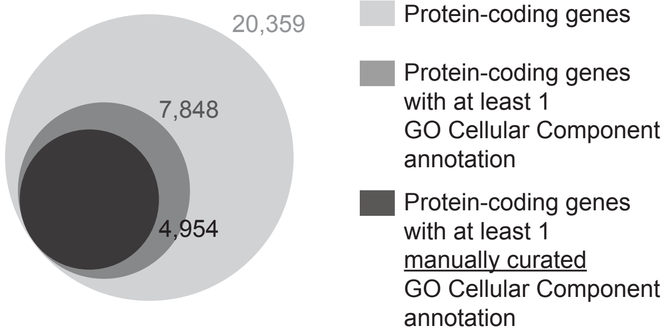

G

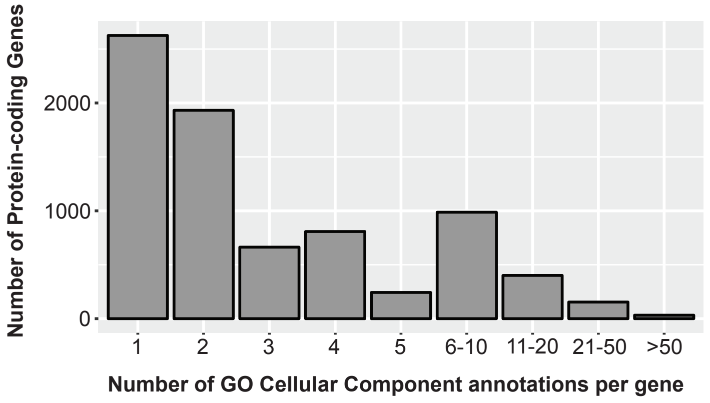

H

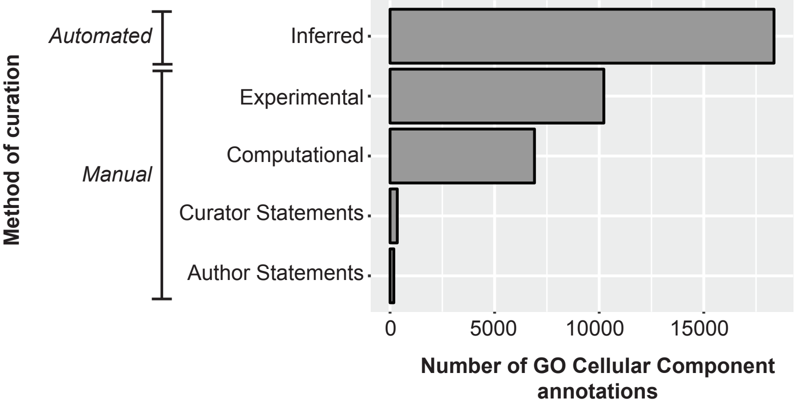

Figure S3

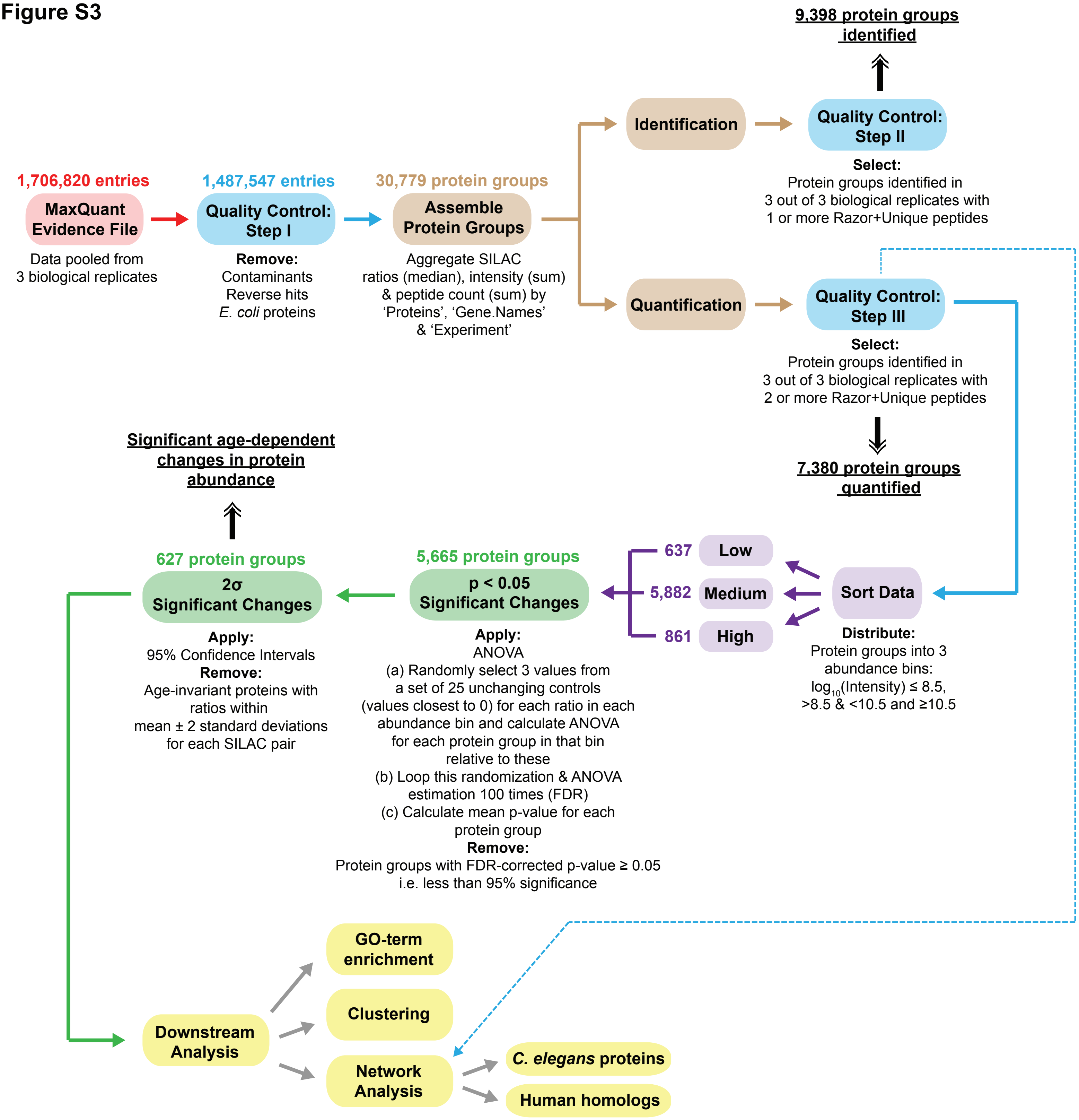

9,398 protein groups identified
Identification
Quality Control: Step II
Select:
Protein groups identified in 3 out of 3 biological replicates with 1 or more Razor+Unique peptides
1,706,820 entries
MaxQuant Evidence File
Data pooled from 3 biological replicates
1,487,547 entries
Quality Control: Step I
Remove:
Contaminants
Reverse hits
E. coli proteins
30,779 protein groups
Assemble Protein Groups
Aggregate SILAC ratios (median), intensity (sum) & peptide count (sum) by 'Proteins', 'Gene.Names' & 'Experiment'
Quantification
Quality Control: Step III
Select:
Protein groups identified in 3 out of 3 biological replicates with 2 or more Razor+Unique peptides
7,380 protein groups quantified
Significant age-dependent changes in protein abundance
627 protein groups
2σ Significant Changes
Apply:
95% Confidence Intervals
Remove:
Age-invariant proteins with ratios within mean ± 2 standard deviations for each SILAC pair
5,665 protein groups
p < 0.05 Significant Changes
Apply:
ANOVA
(a) Randomly select 3 values from a set of 25 unchanging controls (values closest to 0) for each ratio in each abundance bin and calculate ANOVA for each protein group in that bin relative to these
(b) Loop this randomization & ANOVA estimation 100 times (FDR)
(c) Calculate mean p-value for each protein group
Remove:
Protein groups with FDR-corrected p-value ≥ 0.05 i.e. less than 95% significance
637
Low
5,882
Medium
861
High
Sort Data
Distribute:
Protein groups into 3 abundance bins: log10(Intensity) ≤ 8.5, >8.5 & <10.5 and ≥10.5
GO-term enrichment
Clustering
Downstream Analysis
Network Analysis
C. elegans proteins
Human homologs

Figure S4

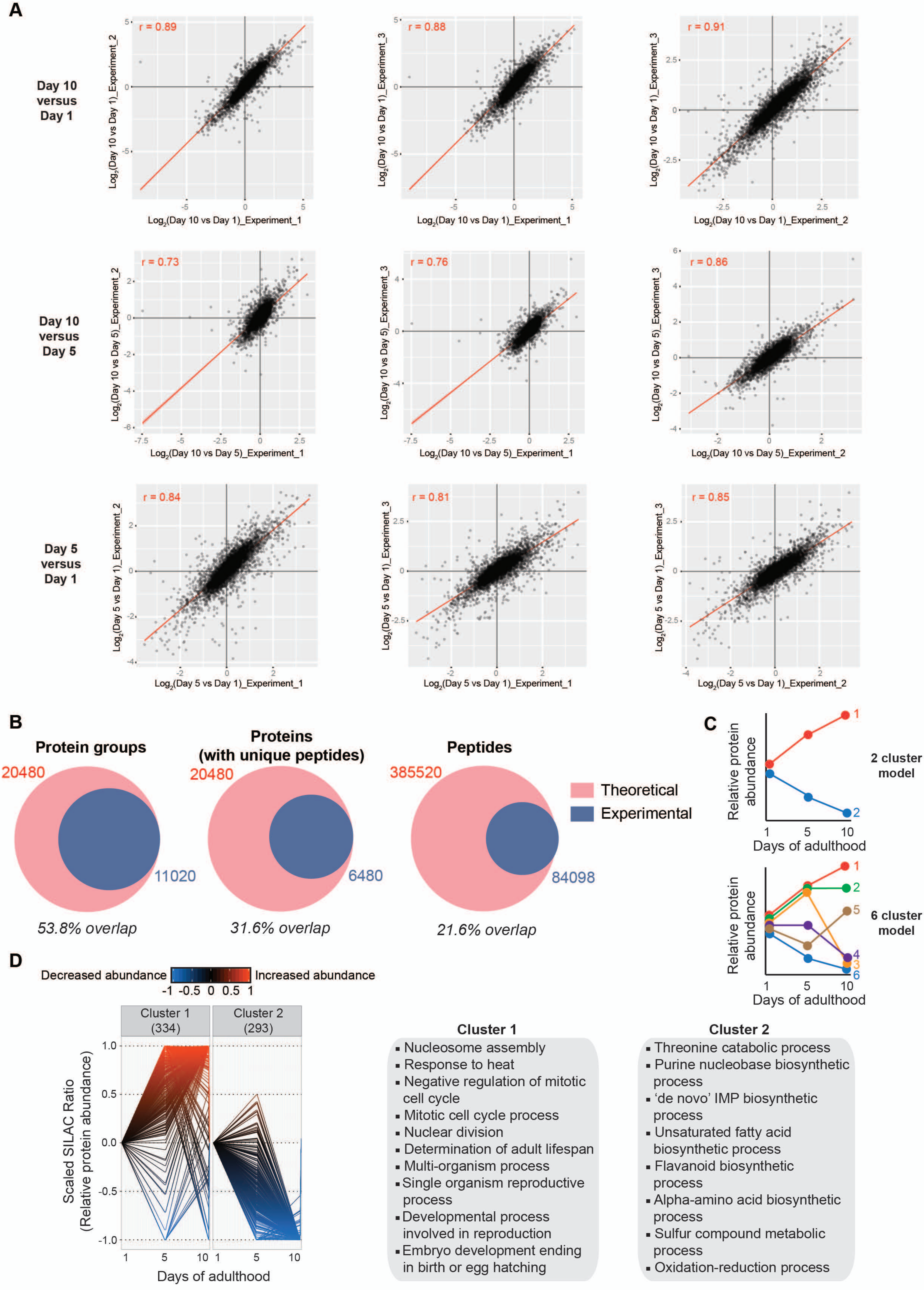
A
Day 10 versus Day 1
r = 0.89
r = 0.88
r = 0.91
Log2(Day 10 vs Day 1)_Experiment_1
Log2(Day 10 vs Day 1)_Experiment_2
Log2(Day 10 vs Day 1)_Experiment_3
Day 10 versus Day 5
r = 0.73
r = 0.76
r = 0.86
Log2(Day 10 vs Day 5)_Experiment_1
Log2(Day 10 vs Day 5)_Experiment_2
Log2(Day 10 vs Day 5)_Experiment_3
Day 5 versus Day 1
r = 0.84
r = 0.81
r = 0.85
Log2(Day 5 vs Day 1)_Experiment_1
Log2(Day 5 vs Day 1)_Experiment_2
Log2(Day 5 vs Day 1)_Experiment_3
B
Protein groups
20480
11020
53.8% overlap
Proteins (with unique peptides)
20480
6480
31.6% overlap
Peptides
385520
84098
21.6% overlap
Theoretical
Experimental
C
Relative protein abundance
Days of adulthood
2 cluster model
6 cluster model
D
Decreased abundance
Increased abundance
-1 -0.5 0 0.5 1
Cluster 1 (334)
Cluster 2 (293)
Scaled SILAC Ratio (Relative protein abundance)
Days of adulthood
Cluster 1
Nucleosome assembly
Response to heat
Negative regulation of mitotic cell cycle
Mitotic cell cycle process
Nuclear division
Determination of adult lifespan
Multi-organism process
Single organism reproductive process
Developmental process involved in reproduction
Embryo development ending in birth or egg hatching
Cluster 2
Threonine catabolic process
Purine nucleobase biosynthetic process
'de novo' IMP biosynthetic process
Unsaturated fatty acid biosynthetic process
Flavanoid biosynthetic process
Alpha-amino acid biosynthetic process
Sulfur compound metabolic process
Oxidation-reduction process

Figure S5

A

Day 10 vs Day 5: Upregulated KEGG pathways

NAME	NES	NOM p-val	FDR q-val
KEGG_VIRAL_MYOCARDITIS	2.1533	0.0000	0.0450
KEGG_DNA_REPLICATION	1.5842	0.0000	0.2558
KEGG_COLORECTAL_CANCER	1.5057	0.0000	0.3044
KEGG_LEUKOCYTE_TRANSENDOTHELIAL_MIGRATION	1.4806	0.0000	0.2396
KEGG_NUCLEOTIDE_EXCISION_REPAIR	1.3546	0.0000	0.5439
KEGG_RNA_DEGRADATION	1.3420	0.0000	0.4912
KEGG_CELL_CYCLE	1.3398	0.0000	0.4274
KEGG_PROGESTERONE_MEDIATED_OOCYTE_MATURATION	1.1921	0.2894	1.0000
KEGG_AMINOACYL_TRNA_BIOSYNTHESIS	1.1787	0.0000	0.9867
KEGG_PATHWAYS_IN_CANCER	1.1783	0.2702	0.8925
KEGG_WNT_SIGNALING_PATHWAY	1.1773	0.2702	0.8155
KEGG_AXON_GUIDANCE	1.1168	0.3110	1.0000
KEGG_SPLICEOSOME	1.0939	0.2903	1.0000
KEGG_BASAL_TRANSCRIPTION_FACTORS	1.0655	0.6190	1.0000
KEGG_ADHERENS_JUNCTION	1.0628	0.0000	0.9950
KEGG_PROSTATE_CANCER	1.0476	0.3488	0.9911
KEGG_VEGF_SIGNALING_PATHWAY	1.0370	0.3295	0.9383
KEGG_TIGHT_JUNCTION	1.0344	0.2702	0.9081
KEGG_PROTEASOME	1.0266	0.2903	0.8652
KEGG_FC_GAMMA_R_MEDIATED_PHAGOCYTOSIS	1.0200	0.3488	0.8333
KEGG_MTOR_SIGNALING_PATHWAY	1.0087	0.3359	0.8329
KEGG_ENDOCYTOSIS	0.9947	0.2903	0.8111
KEGG_UBIQUITIN_MEDIATED_PROTEOLYSIS	0.9890	0.3488	0.7989
KEGG_RENAL_CELL_CARCINOMA	0.9864	0.6391	0.7675
KEGG_MAPK_SIGNALING_PATHWAY	0.9787	0.2602	0.7532

Day 10 vs Day 5: Downregulated KEGG pathways

NAME	NES	NOM p-val	FDR q-val
KEGG_TRYPTOPHAN_METABOLISM	-1.6700	0.0000	0.4179
KEGG_BUTANOATE_METABOLISM	-1.5473	0.0000	0.3489
KEGG_RIBOSOME	-1.5399	0.2780	0.2476
KEGG_PPAR_SIGNALING_PATHWAY	-1.5263	0.0000	0.2384
KEGG_STARCH_AND_SUCROSE_METABOLISM	-1.4929	0.0000	0.2132
KEGG_PEROXISOME	-1.4351	0.0000	0.2485
KEGG_PROPANOATE_METABOLISM	-1.4291	0.0000	0.2617
KEGG_MELANOGENESIS	-1.3842	0.0000	0.2784
KEGG_ARACHIDONIC_ACID_METABOLISM	-1.3644	0.0000	0.2886
KEGG_STEROID_HORMONE_BIOSYNTHESIS	-1.3551	0.0000	0.2974
KEGG_GLYCOLYSIS_GLUCONEOGENESIS	-1.3481	0.0000	0.2744
KEGG_LYSINE_DEGRADATION	-1.3304	0.0000	0.3178
KEGG_ASCORBATE_AND_ALDARATE_METABOLISM	-1.3163	0.0000	0.3209
KEGG_FATTY_ACID_METABOLISM	-1.2962	0.0000	0.3700
KEGG_PYRUVATE_METABOLISM	-1.2432	0.3165	0.4800
KEGG_RETINOL_METABOLISM	-1.2389	0.0000	0.4736
KEGG_DRUG_METABOLISM_CYTOCHROME_P450	-1.2354	0.0000	0.4583
KEGG_METABOLISM_OF_XENOBIOTICS_BY_CYTOCHROME_P450	-1.2303	0.0000	0.4445
KEGG_EPITHELIAL_CELL_SIGNALING_IN_H_PYLORI_INFECTION	-1.2273	0.3419	0.4323
KEGG_CITRATE_CYCLE_TCA_CYCLE	-1.2267	0.2960	0.4129
KEGG_DRUG_METABOLISM_OTHER_ENZYMES	-1.2266	0.0000	0.3954
KEGG_ARGININE_AND_PROLINE_METABOLISM	-1.2201	0.2702	0.3867
KEGG_GLUTATHIONE_METABOLISM	-1.2200	0.3488	0.3718
KEGG_PENTOSE_AND_GLUCURONATE_INTERCONVERSIONS	-1.2043	0.0000	0.3893
KEGG_ALANINE_ASPARTATE_AND_GLUTAMATE_METABOLISM	-1.1700	0.0000	0.4330

B

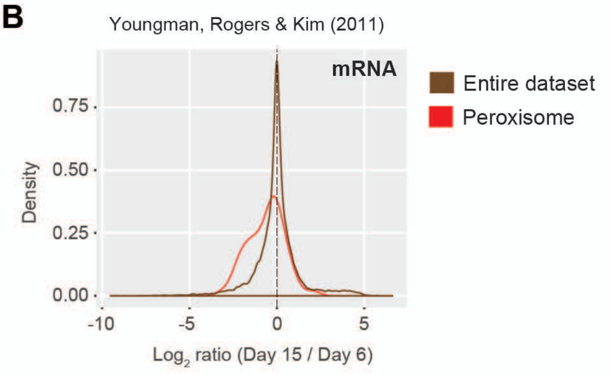

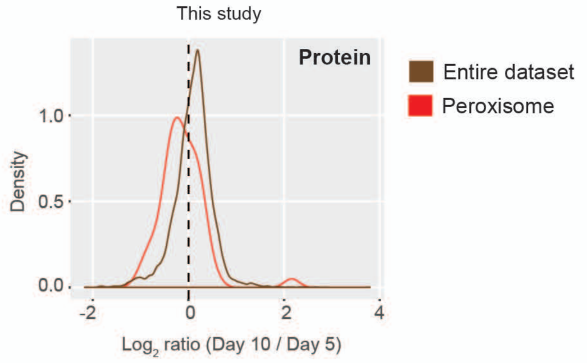

C

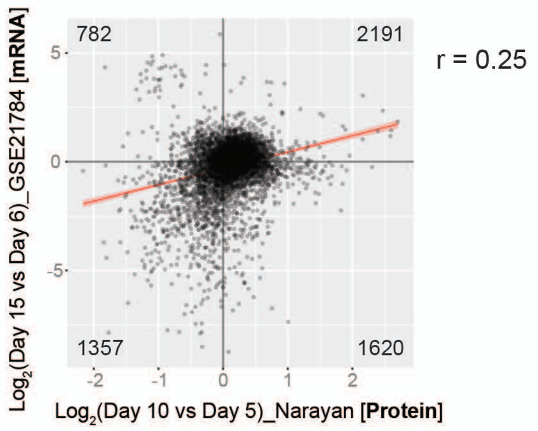

Figure S6

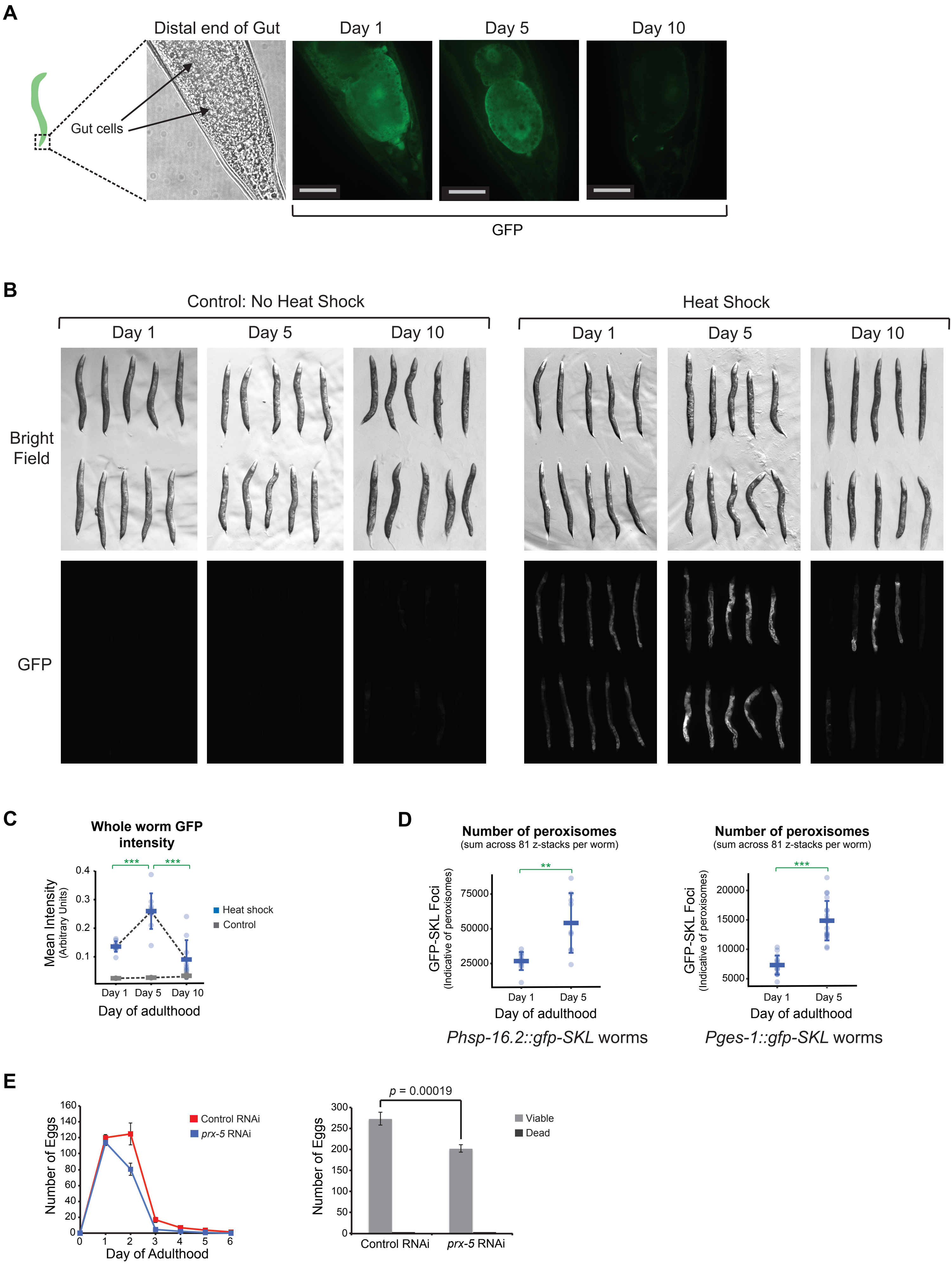
A
Distal end of Gut
Day 1
Day 5
Day 10
Gut cells
GFP
B
Control: No Heat Shock
Heat Shock
Day 1
Day 5
Day 10
Day 1
Day 5
Day 10
Bright Field
GFP
C
Whole worm GFP intensity
Mean Intensity (Arbitrary Units)
0.4
0.3
0.2
0.1
***
***
Heat shock
Control
Day 1
Day 5
Day 10
Day of adulthood
D
Number of peroxisomes
(sum across 81 z-stacks per worm)
GFP-SKL Foci
(Indicative of peroxisomes)
75000
50000
25000
**
Day 1
Day 5
Day of adulthood
Phsp-16.2::gfp-SKL worms
Number of peroxisomes
(sum across 81 z-stacks per worm)
GFP-SKL Foci
(Indicative of peroxisomes)
20000
15000
10000
5000
***
Day 1
Day 5
Day of adulthood
Pges-1::gfp-SKL worms
E
Number of Eggs
160
140
120
100
80
60
40
20
0
0
1
2
3
4
5
6
Day of Adulthood
Control RNAi
prx-5 RNAi
p = 0.00019
Number of Eggs
300
250
200
150
100
50
0
Control RNAi
prx-5 RNAi
Viable
Dead

Figure S7

Microscopy data (n = 20 individual worms except for SQST-1 Day 10 males, where n = 5 individual worms)
Mass spectrometry data (n = 3 biological replicates)
Graphs show sample mean ± standard deviation

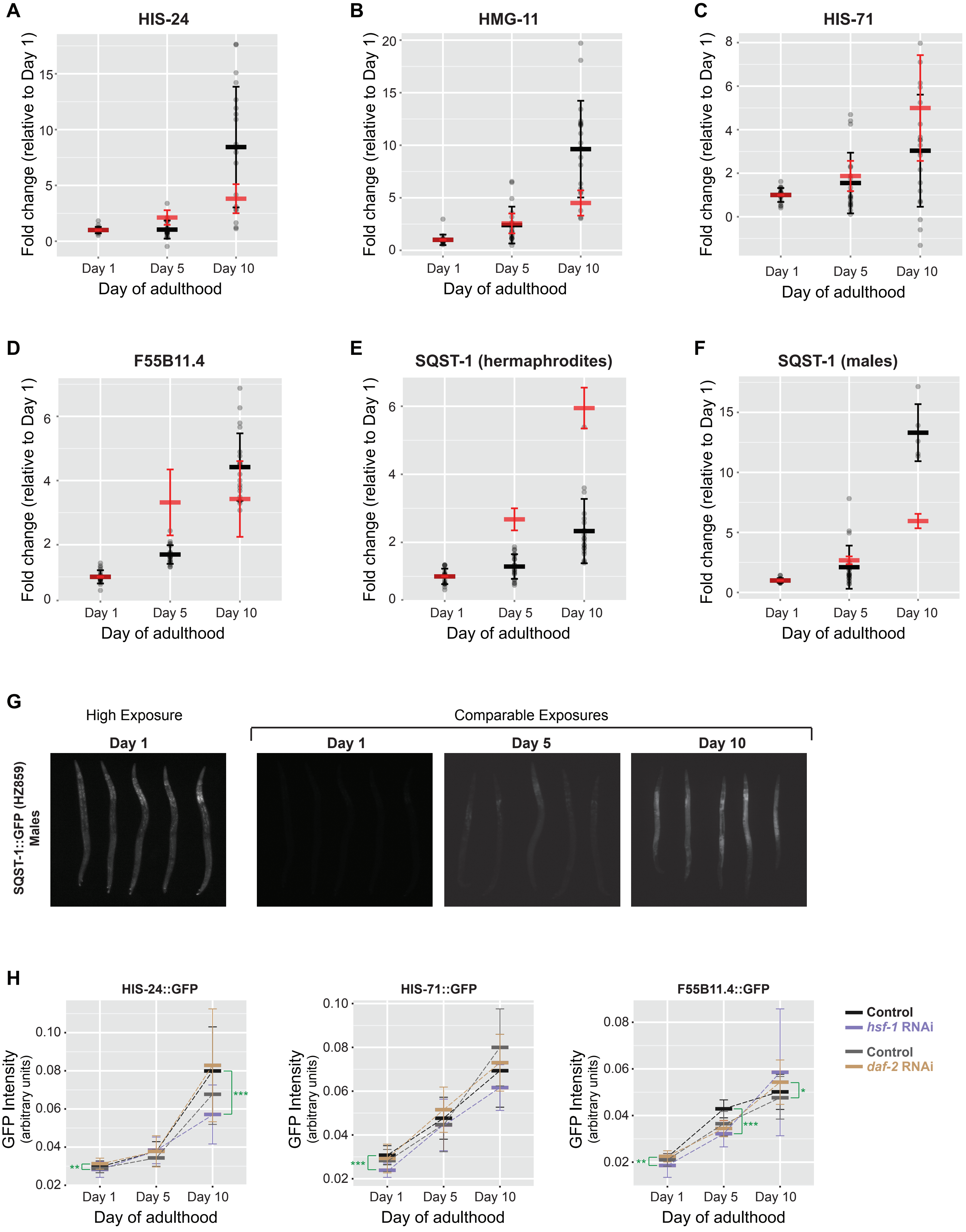

Figure S8

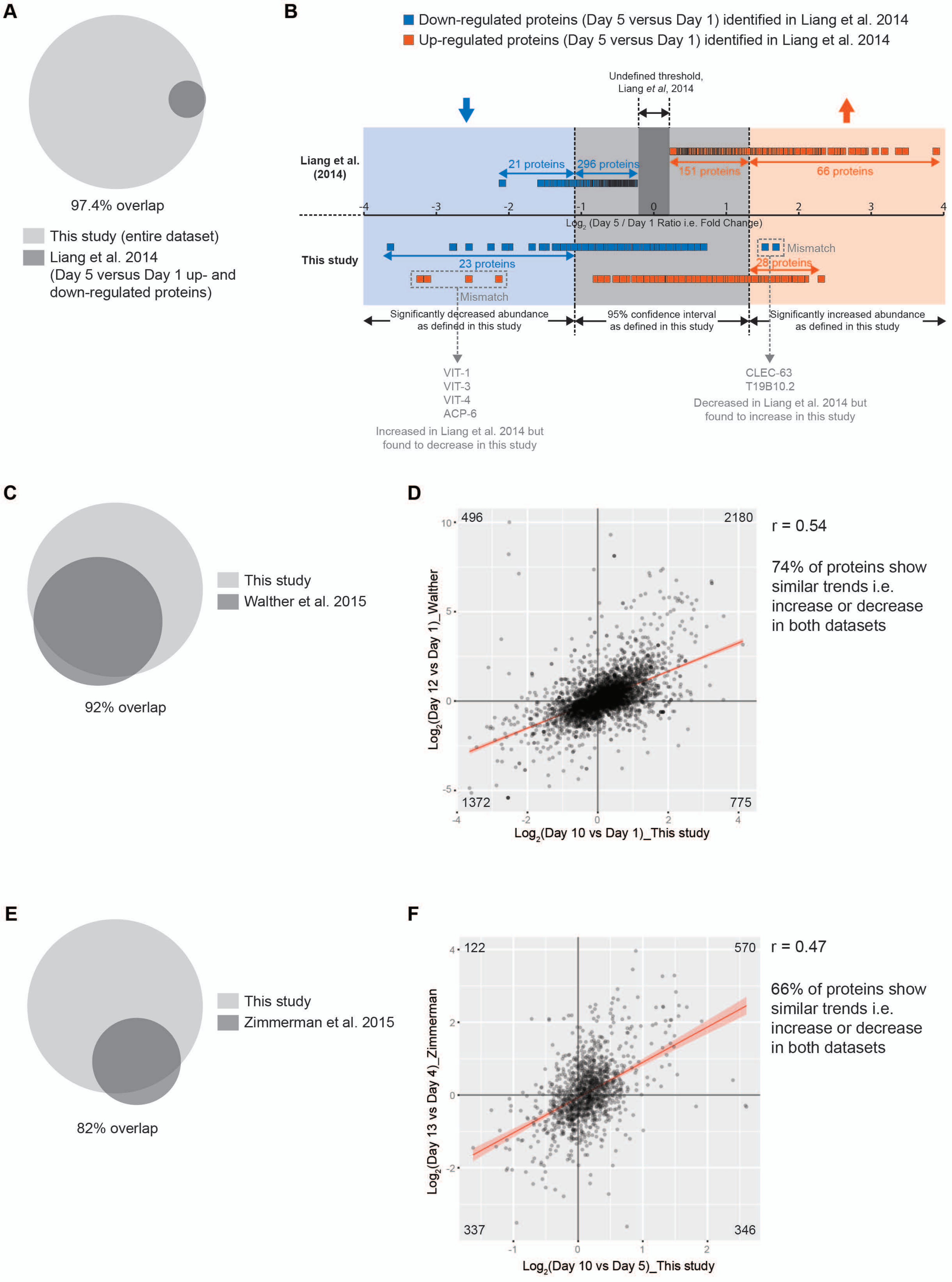
A
97.4% overlap
This study (entire dataset)
Liang et al. 2014
(Day 5 versus Day 1 up- and down-regulated proteins)
B
Down-regulated proteins (Day 5 versus Day 1) identified in Liang et al. 2014
Up-regulated proteins (Day 5 versus Day 1) identified in Liang et al. 2014
Undefined threshold, Liang et al, 2014
Liang et al. (2014)
21 proteins
296 proteins
151 proteins
66 proteins
-4 -3 -2 -1 0 1 2 3 4
Log2 (Day 5 / Day 1 Ratio i.e. Fold Change)
This study
23 proteins
Mismatch
28 proteins
Mismatch
Significantly decreased abundance as defined in this study
95% confidence interval as defined in this study
Significantly increased abundance as defined in this study
VIT-1
VIT-3
VIT-4
ACP-6
Increased in Liang et al. 2014 but found to decrease in this study
CLEC-63
T19B10.2
Decreased in Liang et al. 2014 but found to increase in this study
C
92% overlap
This study
Walther et al. 2015
D
496
2180
1372
775
Log2(Day 12 vs Day 1)_Walther
Log2(Day 10 vs Day 1)_This study
r = 0.54
74% of proteins show similar trends i.e. increase or decrease in both datasets
E
82% overlap
This study
Zimmerman et al. 2015
F
122
570
337
346
Log2(Day 13 vs Day 4)_Zimmerman
Log2(Day 10 vs Day 5)_This study
r = 0.47
66% of proteins show similar trends i.e. increase or decrease in both datasets

SUPPLEMENTAL FIGURE LEGENDS

Figure S1. FUdR treatment does not affect adult *C. elegans* lifespan [Related to Figure 1]
(**A**) Wild-type N2 (Bristol) animals were grown on HT115 *E. coli* at 20°C from hatching and transferred to the indicated RNAi bacteria at mid-late L4. Animals were scored as alive or dead on each day of adulthood until the entire population was dead; missing, bagged or exploded animals were censored. n = 89 (L4440 empty vector RNAi control), n = 94 (*orn-1* RNAi) and n = 78 (*daf-16* RNAi); n does not include censored animals; n = 100 per condition prior to censoring. The graph on the right was plotted without depicting any data points, for clarity, and shows the mean lifespan for wild-type and *orn-1(RNAi)* animals. (**B**) Graph of number of eggs laid in wild-type N2 (Bristol) worms at 20°C between days 1 to 10 of adulthood. Day 1 was considered to be 3 days after hatching (24 h after L4). 20 adult animals were scored; mean eggs laid ± standard error of mean is indicated on the graph. The time points used in this study (days 1, 5 and 10 of adulthood) are highlighted. (**C**) The lifespan of wild-type N2 (Bristol) worms (n = 100 per experiment) at 20°C was measured; missing, bagged or exploded animals were censored. On each day of adulthood, the number of Class A, B and C animals, designated based on motility as described in Herndon et al. 2002, was scored. Shown is the mean ± standard deviation from two independent experiments. (**D**) Graph of the rate of pharyngeal pumping per minute plotted against the age of the animals. Pharyngeal pumping decreases markedly early in adulthood and is virtually undetectable by day 13. Animals (n = 20; see figure for details) were scored for 3 x 30 s intervals every 24 h and the mean pumping rate per minute for each animal calculated. The graph shows the average of the mean pumping rate per minute per worm ± standard error of mean. Pumping could not be detected in some animals after day 7. (**E**) N2 (Bristol) animals were treated with the indicated concentrations of FUdR beginning from the mid-late L4 larval stage. Shown are images taken on day 2 of adulthood (*left*) and total number of eggs laid (*right*; n = 20; graph shows mean ± standard deviation) up to day 10 of adulthood. All FUdR doses tested completely prevented eggs from hatching, as is evident from the images. The total number of eggs laid was significantly lower at doses higher than 50 μM (* = p-value from t-test compared to 0 μM control < 0.05; ** = p-value < 0.01). (**F**) The lifespan of N2 (Bristol) animals on 0 or 50 μM FUdR (treatment was initiated at mid-late L4) at 20°C was scored. Missing, bagged or exploded animals were censored. Shown is the mean ± standard error of mean from 2 independent experiments.

Figure S2. Differential buffer extraction reveals putative sub-cellular localization profiles for ~6,000 *C. elegans* proteins [Related to Figure 1 and Table S1]
(**A**) Venn diagram (not area-proportionate; generated using Interactivenn) showing proteins identified in all 3 biological replicates using the 5-buffer differential extraction method employed in this study that were unique to each buffer, as well as the overlap between buffers. Buffer 1 is a 4% SDS-based buffer (see Supplemental Experimental Procedures) and buffers 2-5 are from the Qiagen QProteome Cell Compartment Kit (CE1, CE2, CE3 and CE4 respectively). CE1 = cytoplasmic fraction, CE2 = membrane fraction, CE3 = nuclear fraction, CE4 = cytoskeletal fraction and insoluble materials. (**B**) Bar plot showing the number of proteins identified using each of the 5 buffers in (A) with 1 or more, 2 or more and 4 or more razor/unique peptides. Also included is the aggregate data wherein peptides identified using each buffer were pooled and compiled into a single dataset ("Combined"). (**C**) Heat map showing the distribution of 6,283 proteins across the indicated sub-cellular compartments, isolated using the QProteome Cell Compartment Kit (Qiagen), in day 1 adult *C. elegans* (also see Table S1, sheet 2). (**D**) 2.5 μg of each sub-cellular fraction or 4% SDS-extracted whole-cell lysate were run out on a 4-12% NuPAGE gel (Thermo Fisher) and analyzed by immunoblotting with the following antibodies: anti-GAPDH (Sigma; 1:5000), anti-Histone H3 (Abcam; 1:4000) and anti-ATP synthase subunit α (Mitosciences; 1:2000). (**E**) GO-term enrichment (Cellular Component) of the proteins present in each sub-cellular compartment was performed using PANTHER and plotted using REVIGO, in order to validate the efficacy of the extraction methodology in nematodes. The size of the bubbles is indicative of the number of proteins annotated with that GO-term (range: 9-309 for cytoplasm; 7-168 for membrane; 5-14 for nucleus; 4-60 for cytoskeleton); bubbles are color-coded according to significance (Bonferroni-corrected p-value). To reduce GO-term redundancy, the 'PANTHER GO-Slim Cellular Component' annotation data set was used unless the output was predominantly "unclassified", in which case 'GO Cellular Component complete' was used. Only those proteins whose levels in a single fraction were found to be 75% or more of their total abundance across all fractions were included in the analysis [i.e. (intensity in a single fraction / sum of intensities across all 4 fractions) x 100 >= 75]. This subset included 2,297 cytoplasmic proteins, 354 membrane proteins, 78 nuclear proteins and 321 cytoskeletal proteins (see Table S1, sheets 3-6). (**F**) Area-proportionate Venn diagram (generated using BioVenn) showing that only ~39% of the theoretical *C. elegans* protein-coding genes (20,359 according to

WormBase Release WS250, 31 July 2015) have at least one Cellular Component GO-term annotation (7,848; GO_CC), and of these, only 4,954 have a manually curated GO_CC annotation. (**G**) Histogram showing the number of GO_CC annotations assigned to each of the 7,848 *C. elegans* protein-coding genes with a GO_CC annotation. (**H**) Graph showing the breakdown of GO_CC assignments to the 7,848 *C. elegans* protein-coding genes with at least one GO_CC annotation. As stated in (F), 4,954 of these genes (63%) have a manually curated GO_CC annotation. However, only a subset of the manually curated GO_CC annotations (~10,000 annotations, corresponding to 2,121 protein-coding genes) is based on experimental evidence. Thus, only about 10% (2,121 out of 20,359) of all *C. elegans* protein-coding genes have a GO_CC assignment based on experimental data. (**F-H**) Raw data was obtained from WormBase, and mined/queried using WormMine.

Figure S3. Data analysis workflow [Related to Figures 1 and 2, and Tables S2-S4]
Schematic representation of the data analysis pipeline used in this study.

Figure S4. Assessment of proteomics data quality [Related to Figures 1 and 2]
(**A**) Scatter plots depicting log_2-transformed SILAC ratios (for each pair-wise comparison of the 3 time-points used in this study) between the 3 biological replicates. Reproducibility of protein quantification across the replicates was high, as indicated by the Pearson's r values shown on the upper left corner of each scatter plot. (**B**) *Left*: Area-proportionate Venn diagram (generated using BioVenn) showing the overlap between proteins identified in this study (adults only; "Experimental") versus the theoretical *C. elegans* proteome comprising all developmental stages, as defined by WormBase (Release WS243, 28 March 2014 containing 20,480 protein-coding genes; "Theoretical"). *Center*: Area-proportionate Venn diagram of the theoretical *C. elegans* proteome (as above) and the number of proteins reproducibly identified with sequence-unique peptides in this study ("Experimental"). The "Experimental" dataset here does not, therefore, include peptides that were assigned to protein groups rather than individual proteins. *Right*: A theoretical tryptic digest of the *C. elegans* proteome was generated and a list of putative MS-flyable peptides compiled (conditions imposed: peptides must have at least one Lys or Arg, and must be 6-35 amino acids in length; "Theoretical"). This list was compared to the sequence-unique peptides identified in this study ("Experimental") and is represented as an area-proportionate Venn diagram. (**C**) Cartoons showing representative profiles of the 627 age-variant proteins (see main Figure 2) grouped by hierarchical clustering into either 2 clusters (*top*) or 6 clusters (*bottom*). (**D**) Hierarchical clustering of the 627 proteins using Ward's minimum variance method into 2 groups based on trend profiles. To minimize redundancy in clustering due to large differences in the magnitude of the abundance changes, the SILAC ratios were scaled to range between -1 (maximum decrease) and +1 (maximum increase) with ratios near 0 indicating no change in abundance during the 3 time points considered in this study. Results from GO-term enrichment analysis (multiple hypothesis [Bonferroni] corrected $p < 0.05$) of each cluster using PANTHER are also depicted.

Figure S5. Gene Set Enrichment Analysis of proteomics data reveals pathways altered during nematode aging [Related to Figures 3 and 4]
(**A**) Protein quantification data for day-10 versus day-5 of adulthood, as measured in this study, were analyzed using gene set enrichment analysis to identify KEGG pathways that change during aging. Shown is the GSEA output using the algorithm from the Broad Institute. (**B**) Density plots depicting the abundance of a gene set comprising KEGG 'peroxisome' genes relative to the entire dataset. The curve for peroxisome genes includes those only those genes that were detected both in a study by Youngman et al. 2011 measuring mRNA levels and in this study, measuring protein levels. *Top*: density plot for peroxisome mRNA transcripts relative to the entire Youngman et al. 2011 mRNA dataset; *Bottom*: density plot for peroxisome protein levels relative to the entire protein dataset measured in this study. (**C**) Scatter plot showing mRNA levels measured in a study by Youngman et al. 2011 plotted against the levels of their corresponding protein products as measured in this study. The mRNA and protein data show poor correlation ($r = 0.25$ [Pearson]). A linear model, calculated using the function lm in R, is depicted by the red line. 95% confidence intervals for the linear model are shaded light red.

Figure S6. Control experiments for GFP-SKL *C. elegans* strains [Related to Figure 5]
(**A**) In order to ensure that the GFP-SKL foci observed in young *Phsp-16.2::gfp-SKL* worms after induction of GFP-SKL expression by heat shock were not merely artifacts or stress granules, *Phsp-16.2::gfp* animals, lacking the peroxisome-targeting -SKL signal were used (strain CL2070). No foci were detected in these animals after heat shock. Similar to data from GFP-SKL animals (see below), *hsp-16.2* driven GFP expression was markedly decreased by day 10 of adulthood. (**B & C**) Whole-body *hsp-16.2*-driven GFP-SKL intensity was measured and found to increase significantly between days 1

and 5 of adulthood, and then drop markedly by day 10 (p-value from unpaired t-test = 1.02×10^{-4} for day 1 versus day 5; 1.70×10^{-5} for day 5 versus 10; and 0.067 for day 1 versus day 10. n = 10 in all cases). Shown are bright field and GFP images taken with (*right*) and without (*left*) heat shock (**B**) as well as a graph showing quantification of GFP intensity from the images (**C**). (**D**) The number of foci resolved ("peroxisomes") at day 1 and day 5 (based on data presented in main Figures 5C and 5D) was summed across the entire z-stack (81 sections per worm) for GFP-SKL driven by the *hsp-16.2* promoter (*left*) and the *ges-1* promoter (*right*). The number of dots resolved was found to increase significantly by day 5 (p-value from unpaired t-test = 0.0048 (*left*) and 3.07×10^{-9} (*right*)). (**E**) N2 (Bristol) worms were treated with control or *prx-5* RNAi from late L4/young adult stage, and the number of eggs laid on each day of adulthood measured (*left*). Also shown is the total number of eggs laid during days 1 to 10 of adulthood (*right*). Graphs show mean ± standard deviation (n = 20 for control RNAi animals and n = 26 for *prx-5(RNAi)* animals). The total number of eggs laid in *prx-5* RNAi animals was found to be significantly less than control animals using t-test (p-value = 0.00019).

Figure S7. Comparison of mass spectrometric predictions with *in vivo* measurements of age-dependent protein changes by microscopy [Related to Figure 6]
(**A-F**) Graphs showing mass spectrometry-determined changes in protein abundance during aging (*red*) compared to *in vivo* fold-change measurements calculated by imaging GFP::protein fusion worm strains (*black*). For microscopy, 20 individual worms were analyzed (see (**G**) and main Figure 6A for snapshots); quantification was performed using Cell Profiler (Broad Institute). Shown is the mean ± standard deviation. Note that the individual variation in protein expression for all GFP::protein fusion strains increased markedly by day 10. SQST-1::GFP worms (strain HZ859) were in a *him-5(e1490)* background; thus, both hermaphrodites (**E** and main Figure 6A) and males (**F, G**) were examined. (**H**) HIS-24::GFP (*left*), HIS-71::GFP (*center*) and F55B11.4::GFP-expressing (*right*) worms were grown on the indicated RNAi (initiated at L1 stage) and GFP-fluorescence was quantified on days 1, 5 and 10 of adulthood. Graphs show mean ± standard deviation (n = 20 worms). Significance was calculated using an unpaired t-test (p-value < 0.05 = *, < 0.01 = **, < 0.001 = ***). The *daf-2* and *hsf-1* RNAi are in different vector backbones (see Supplemental Experimental Procedures); thus, the corresponding empty vector control for each was used.

Figure S8. Comparison between this study and recent proteomics studies (Liang et al. 2014; Walther et al. 2015; Zimmerman et al. 2015) [Related to Figure 1 and Discussion]
(**A**) Area-proportionate Venn diagram showing that 97.4% of the proteins for which quantification data from the Liang et al. 2014 study was publicly available, were also detected in our study. (**B**) Significant fold-changes as determined in the Liang et al. 2014 study based on undefined thresholds, were projected onto our thresholds for significance (95% confidence interval calculated from the mean ± 1.96 standard deviations based on quantification of 7,380 proteins from 3 biological replicates). The majority of the changes deemed significant in the Liang et al. 2014 study were not scored as significant in our study based on our stricter thresholds. The Liang et al. 2014 data (*upper panel*) is compared to our quantification data (*lower panel*) for the same proteins. Also indicated are the mismatches (6 proteins) between the two datasets. (**C**) Area-proportionate Venn diagram showing that 92% of the proteins detected in the Walther et al. 2015 study were also detected in our study. (**D**) Scatter plot for proteins detected in both the Walther et al. 2015 study and this study, showing reproducibility of protein quantification between the studies. A modest correlation was observed (r = 0.54 [Pearson]); this is likely adversely affected by the fact that different quantification strategies were used in the two studies (spike-in SILAC versus triple-SILAC). Additionally, the measured time-points were not identical (day 21 / day 1 data from the Walter et al. 2015 study were compared to day 10 / day 1 data from our study). A linear model, calculated using the function lm in R, is depicted by the red line. 95% confidence intervals for the linear model are shaded light red. Although the magnitude of the fold-change for each protein in the datasets was only moderately similar (reflected in the modest r = 0.54), 74% of the proteins showed similar trends (i.e. increase or decrease) in both datasets. (**E**) Area-proportionate Venn diagram showing that 82% of the proteins detected in the Zimmerman et al. 2015 study were also detected in our study. (**F**) Scatter plot for proteins detected in both the Zimmerman et al. 2015 study and this study, showing reproducibility of protein quantification between the studies. A modest correlation was observed (r = 0.47 [Pearson]); this is likely adversely affected by the fact that different quantification strategies were used in the two studies (dimethyl labeling versus SILAC). Additionally, the measured time-points were not identical. A linear model, calculated using the function lm in R, is depicted by the red line. 95% confidence intervals for the linear model are shaded light red.

SUPPLEMENTAL EXPERIMENTAL PROCEDURES

Reagents

EZQ protein assay, Triscarboxyethylphosphine (TCEP), CBQCA assay, Pepmap C18 (2 cm x 75 µm) trap columns and EasySpray C18 columns (2 µm particles, 50 cm x 75 µm) were from Thermo Fisher Scientific (Waltham, MA). MS-Grade Trypsin was from Promega. Sep-Pak tC18 µ-elution 96-well plates were purchased from Waters. Purified arginine and lysine for SILAC experiments were from Cambridge Isotopes Laboratories (Tewksbury, MA) via CK Gas and were as follows: Light (Arg0, ULM-8347; Lys0, ULM-8766); Medium (Arg6, CLM-2265; Lys4, DLM-2640); and Heavy (Arg10, CNLM-539; Lys8, CNLM-291). All other materials were obtained from Sigma, unless otherwise indicated.

RNAi experiments

RNA interference experiments were performed using the RNAi via feeding strategy [Ahringer library; (Kamath et al. 2001)]. The exception was the *daf-2* RNAi construct used (pAD48-*daf-2*-RNAi; Addgene plasmid #34834 and corresponding empty vector control pAD12; Addgene plasmid #34832), which was made in-house, and has been characterized previously (Dillin et al. 2002). RNAi of *orn-1* for SILAC experiments was performed as described previously (Larance et al. 2015; Larance et al. 2011). *prx-5* RNAi was initiated during 'day 0 of adulthood', just prior to the appearance of eggs in the hermaphrodite animal, as described in Zhou et al. 2012. All other RNAi experiments were initiated at the L1 stage and the resulting adults were imaged as described in the main Experimental Procedures.

Lifespan assays

Lifespan assays were performed at 20°C as described previously, except that days of adulthood (day 1 = 24 h post L4) were reported in our study (Hsin & Kenyon 1999). Additionally, for *orn-1* and *daf-16* RNAi experiments, RNAi treatment was initiated at L4 and FUdR was not used; the animals were instead transferred to fresh RNAi plates every other day. For FUdR lifespan assays (dose response), synchronized wild-type L4 animals were transferred onto to NGM/OP50 plates containing the required concentration of FUdR and assessed for survival on each day of adulthood. Animals that exploded, bagged or were missing were censored in all cases.

Egg-laying assays

Egg-laying assays were performed at 20°C. In brief, N2 animals were synchronized as L1 larvae by filtering [11 µm filter; (Larance et al. 2011)] and at L4, 30 animals were transferred onto fresh 6 cm NGM/OP50 plates at 3 animals per plate. 24 h post L4 (day 1), the adults were transferred onto fresh NGM/OP50 plates and this was repeated every 24 h until no more eggs were laid. On each day, the plates containing laid eggs and/or L1 larvae (from which the adults were transferred onto new plates) were incubated at 25°C overnight, followed by a 30 min incubation at 4°C to temporarily immobilize the animals. Dead eggs and larvae (viable eggs) were scored. For FUdR egg-laying assays, FUdR treatment was initiated at L4 and adults were transferred onto fresh NGM/OP50 plates containing FUdR on each day of adulthood. *prx-5* RNAi treatment was initiated at day 0 of adulthood, as described previously (Zhou et al. 2012) and adults were transferred onto fresh RNAi plates on each day of adulthood.

Determination of class A, B, C animals

Class A, B, C animals were identified as described previously (Herndon et al. 2002). Measurements were made at 20°C using N2 (Bristol) animals. Experiments were performed in a similar manner to lifespan assays (see above) although no FUdR was used, and animals were scored as being class A, B or C on every other day of adulthood. Exploded, bagged or missing animals were censored. In brief, animals that moved well were scored as Class A, animals showing uncoordinated movements were scored as Class B, and animals that showed little or no movement were scored as Class C.

Pumping assays

Synchronized single N2 (Bristol) animals maintained at 20°C were observed. On each day of adulthood, the number of contractions in the terminal bulb of the pharynx was counted for 3 x 30 s intervals (n = 20 or as indicated in the figure legends).

Microscopy and imaging

Induction of GFP-SKL expression from the *hsp-16.2* promoter was carried out as follows: day-1 adults (or as indicated in the figure legends) grown at 20°C were shifted for 2 h to 34°C, followed by 30 min at 20°C, then 1 h at 34°C, after which the worms were returned to 20°C for 12 h. Animals were

anesthetized on 2% agarose pads in 10 mM levamisole for no more than 10 min immediately prior to viewing. Imaging of GFP-SKL strains (intestinal cells) was performed using an UltraVIEW Vox CSU-X1 confocal imaging system (Perkin Elmer), incorporating an Olympus IX70 microscope, a 488 nm solid-state laser, and an Olympus 60X/1.3 NA oil-immersion objective. Images were processed using Volocity 15 software (Perkin Elmer) and analyzed using Cell Profiler (Lamprecht et al. 2007) as follows: z-sections were taken over 40 μm at 0.5 μm intervals. The 81 resulting images per worm were quantified individually for peroxisomes (using a modification of the speckle counting tool in Cell Profiler) and cytosol (total GFP intensity minus peroxisomes). Mean intensities from each section were then summed to get total intensity, peroxisomal intensity and cytosolic intensity per worm. These were then graphed using R. Additionally, the number of peroxisomes was counted and graphed from the sum of the 81 z-stacks as well as from the single z-section that had the maximum number of peroxisomes. All other microscopy was performed using a Leica MZ16FA microscope (Leica; Bannockburn, IL) and images were analyzed using Cell Profiler. Here, fluorescence intensity in the whole animal was measured and subtracted from background fluorescence (i.e. mean fluorescence) outside the worm and plotted using R.

SILAC labeling, sample preparation, fractionation and clean-up

For isotopic labeling, N2 (Bristol) worms were grown on NGM-N plates at 20°C as described previously (Larance et al. 2011) with the following adaptations: 25 mid-late L4 larvae were picked on to NMG-N plates supplemented with 1 mM IPTG and 1 μg/ml carbenicillin, and seeded with *E. coli* SLE1 grown in light, medium or heavy label as described previously (Larance et al. 2011). Labeling was for 2 generations at 20°C. L1 animals from the F2 generation were collected by filtering through an 11 μm filter (Millipore) and 40,000 L1 larvae per condition (light, medium or heavy) were transferred to 9 cm NGM-N plates seeded with labeled SLE1 at 4,000 worms per plate. Approximately 2 days later, when the worms were mid-late L4 animals, they were washed off the plates with M9 buffer and transferred to fresh NGM-N plates (seeded with the required labeled bacteria) containing 50 μM FUdR. Care was taken to ensure the animals did not starve during the experiment – if required, additional concentrated, labeled SLE1 was added to the plates. The animals were then collected as required (Heavy – Day 1 adults, Medium – Day 5 and Light – Day 10) by washing off the plate with M9 buffer, followed by a further 3-5 washes in M9 to remove bacteria and eggs. Worms were collected by light centrifugation or by gravity at room temperature (5-10 min in a falcon tube on the bench). Worm pellets were snap-frozen in liquid N_2 and upon thawing, were mixed at a 1:1:1 ratio by volume of heavy:medium:light. 5% of the mixture was lysed directly in SDS buffer (4% SDS, 150 mM NaCl, 10 mM phosphate buffer pH 7, 50 mM N-ethylmaleimide, 25 mM TCEP) as follows: 2x volume of buffer and 1x volume of 0.7 mM Zirconia beads (BioSpec) were added and the sample was lysed by bead beating (Mini Beadbeater 8, BioSpec) at max speed for 3 x 20 s with 20 s intervals. The sample was then heated to 65°C for 10 min, centrifuged at 16,100 g for 15 min at room temperature and the supernatant collected. The remainder of the worm mixture (95%) was fractionated using the Q-Proteome Cell Compartment Kit (Qiagen) as described previously (Pourkarimi et al. 2012), but with the following modifications: (1) worm pellets were aliquotted into 100 μl aliquots and each aliquot was processed separately; (2) 100 μl 0.7 mM Zirconia beads were used per aliquot, together with 250 μl buffer CE1; subsequent buffers were scaled accordingly, maintaining the ratios recommended by the manufacturer; (3) homogenizing using a Qiashredder (Qiagen) was adjusted as follows: the sample was added to a shredder and centrifuged for 10 s at 100 g, after which the eluate was transferred to a fresh shredder and the process repeated. SDS, TCEP and NEM were added to each fraction, to final concentrations of 2%, 25 mM and 50 mM respectively, following which the samples were heated at 65°C for 10 min. Equal amounts of protein (500 μg, measured by EZQ Assay) from the SDS-lysate and sub-cellular fractions were concentrated by chloroform-methanol precipitation and resuspended in 8 M urea in 100 mM triethylammonium bicarbonate (TEAB). Samples were diluted 8-fold using 100 mM TEAB and supplemented with $CaCl_2$ to a final concentration of 1 mM. Trypsin was added at a 1:50 ratio of enzyme to protein and incubated for 16 h at 37°C. Sample desalting and offline SAX chromatography was performed as described previously (Larance et al. 2015; Ly et al. 2014).

LC-MS/MS and analysis of spectra

~1 μg of each desalted SAX fraction containing peptides in 5% (vol/vol) formic acid (~10 μl) was injected onto a 0.3 mm id × 5 mm PepMap-C18 pre-column and chromatographed on a 75 μm × 15 cm PepMap-C18 column. Using the following mobile phases: 2% ACN incorporating 0.1% FA (Solvent A) and 80% ACN incorporating 0.1% FA (Solvent B), peptides were resolved using a linear gradient from 5% B to 35% B over 4 hours with a constant flow of 200 nL min^{-1} as described previously (Ly et al. 2014). Peptides were ionized by electrospray ionization at +2.0 kV. Tandem mass spectrometry analysis was carried out on a Q-Exactive mass spectrometer (Thermo Fisher Scientific) using HCD

fragmentation. The data-dependent acquisition method used acquired MS/MS spectra on the top 10 most abundant ions at any one point during the gradient. The raw data produced by the mass spectrometer were analysed using the quantitative proteomics software MaxQuant including the Andromeda search engine (http://www.maxquant.org, version 1.4.1.2) (Cox & Mann 2008). Peptide and protein level identification were both set to a false discovery rate of 1% using a target-decoy based strategy. The databases supplied to the search engine for peptide identifications were both the *C. elegans* (December 2013) and *E. coli* Uniprot databases (July 2012) containing 22,998 and 4,431 entries respectively. The mass tolerance was set to 7ppm for precursor ions and MS/MS mass tolerance was set at 20 ppm. Enzyme was set to trypsin/p with up to 2 missed cleavages. Deamidation of Asn and Gln, oxidation of Met, pyro-Glu (with N-term Gln), and acetylation of the protein N-terminus were set as variable modifications. N-ethylmaleimide on Cys was searched as a fixed modification. The output from MaxQuant provided peptide level data as well as protein level data, grouped by protein isoforms. Protein groups were defined as a set of unique peptides that are either shared between multiple protein isoforms, or belong to a single protein isoform (Figure S3). While this may lead to higher redundancy it enables us to quantify changes in individual protein isoforms.
All raw proteomics data from this study have been deposited to the ProteomeXchange Consortium (http://proteomecentral.proteomexchange.org) via the MassIVE partner repository with the dataset identifiers PXD004584 (ProteomeXchange) and MSV000079263 (MassIVE). The processed data are additionally provided in a searchable, online database (https://www.peptracker.com/epd/).

Data analysis, statistics, homology mapping and graphing
Most of the subsequent data analysis was done using R version 3.1.3 (R Core Team n.d.) employing Rstudio 0.98.1091 (RStudio Team n.d.) and the ggplot2 package for generating graphs (Wickham 2009). The MaxQuant output file "evidence.txt" was used to compile protein groups as depicted in Figure S3; the MaxQuant "proteinGroups.txt" output file was used for analysis of the sub-cellular fractionation data presented in Figure S2 (see Table S1). Only those proteins identified in all 3 biological replicates with at least 2 razor+unique peptides were included in our "quantified proteins" dataset (Table S3). As measurement error correlates inversely with intensity, prior to p-value calculations by ANOVA, the dataset was grouped into three bins based on protein abundance (i.e. measured intensity). Control proteins for each ANOVA calculation were randomly sampled from a set of age-invariant proteins (with SILAC pair ratios or $\log_2$ fold changes ≈ 0) in the same abundance bin as the protein in consideration. The random sampling and ANOVA calculation for each protein was looped 100 times and used to estimate the false discovery rate (FDR). GO-term enrichment was performed using DAVID (Huang, Sherman & Lempicki 2009a; Huang, Sherman & Lempicki 2009b) unless otherwise indicated, with the full *C. elegans* proteome supplied by DAVID used as the background list, and plotted to reduce redundancy using ReviGo (Supek et al. 2011); Venn diagrams were generated using BioVenn (Hulsen et al. 2008). PCA analysis was done using Partek Genomics Suite (Partek Inc, St. Louis, MO). GSEA analysis was done using the algorithm from the Broad Institute (Subramanian et al. 2005) using either a user-defined gene set or the KEGG pathway database (Kanehisa et al. 2014; Kanehisa & Goto 2000). Human homologs of *C. elegans* genes were obtained using information from the WormBase FTP site (ftp://ftp.wormbase.org/pub/wormbase/); files containing both best BLAST hits and orthologs were used and merged into a single file using R. Microscopy images were analyzed and quantified using Cell Profiler (Lamprecht et al. 2007).

SUPPLEMENTAL REFERENCES

Cox, J. & Mann, M., 2008. MaxQuant enables high peptide identification rates, individualized p.p.b.-range mass accuracies and proteome-wide protein quantification. *Nature biotechnology*, 26(12), pp.1367–1372.

Dillin, A., Crawford, D.K. & Kenyon, C., 2002. Timing requirements for insulin/IGF-1 signaling in C. elegans. *Science (New York, N.Y.)*, 298(5594), pp.830–834.

Herndon, L.A. et al., 2002. Stochastic and genetic factors influence tissue-specific decline in ageing C. elegans. *Nature*, 419(6909), pp.808–814.

Hsin, H. & Kenyon, C., 1999. Signals from the reproductive system regulate the lifespan of C. elegans. *Nature*, 399(6734), pp.362–366.

Huang, D.W., Sherman, B.T. & Lempicki, R.A., 2009a. Bioinformatics enrichment tools: paths toward the comprehensive functional analysis of large gene lists. *Nucleic acids research*, 37(1), pp.1–13.

Huang, D.W., Sherman, B.T. & Lempicki, R.A., 2009b. Systematic and integrative analysis of large gene lists using DAVID bioinformatics resources. *Nature protocols*, 4(1), pp.44–57.

Hulsen, T., de Vlieg, J. & Alkema, W., 2008. BioVenn – a web application for the comparison and visualization of biological lists using area-proportional Venn diagrams. *BMC Genomics*, 9(1), p.488.

Kamath, R.S. et al., 2001. Effectiveness of specific RNA-mediated interference through ingested double-stranded RNA in Caenorhabditis elegans. *Genome biology*, 2(1), p.RESEARCH0002.

Kanehisa, M. & Goto, S., 2000. KEGG: kyoto encyclopedia of genes and genomes. *Nucleic acids research*, 28(1), pp.27–30.

Kanehisa, M. et al., 2014. Data, information, knowledge and principle: back to metabolism in KEGG. *Nucleic acids research*, 42(Database issue), pp.D199–205.

Lamprecht, M.R., Sabatini, D.M. & Carpenter, A.E., 2007. CellProfiler™: free, versatile software for automated biological image analysis. *Biotechniques*.

Larance, M. et al., 2015. Global proteomics analysis of the response to starvation in C. elegans. *Molecular & cellular proteomics : MCP*, 14(7), pp.mcp.M114.044289–2001.

Larance, M. et al., 2011. Stable-isotope labeling with amino acids in nematodes. *Nature methods*, 8(10), pp.849–851.

Ly, T. et al., 2014. A proteomic chronology of gene expression through the cell cycle in human myeloid leukemia cells J. Pines, ed. *eLife*, 3, p.e01630.

Pourkarimi, E., Greiss, S. & Gartner, A., 2012. Evidence that CED-9|[sol]|Bcl2 and CED-4|[sol]|Apaf-1 localization is not consistent with the current model for C. elegans apoptosis induction. *Cell Death & Differentiation*, 19(3), pp.406–415.

R Core Team, R: A Language and Environment for Statistical Computing. Available at: http://www.R-project.org/.

RStudio Team, RStudio: Integrated Development Environment for R. Available at: http://www.rstudio.com/.

Subramanian, A. et al., 2005. Gene set enrichment analysis: a knowledge-based approach for interpreting genome-wide expression profiles. *Proceedings of the National Academy of Sciences of the United States of America*, 102(43), pp.15545–15550.

Supek, F. et al., 2011. REVIGO summarizes and visualizes long lists of gene ontology terms. C. Gibas, ed. *PloS one*, 6(7), p.e21800.

Wickham, H., 2009. *ggplot2: elegant graphics for data analysis*, New York, NY: Springer New York. Available at: http://had.co.nz/ggplot2/book.

Zhou, B. et al., 2012. Midlife gene expressions identify modulators of aging through dietary interventions. *Proceedings of the National Academy of Sciences of the United States of America*, 109(19), pp.E1201–9.
